# Investigating the pertussis resurgence in England and Wales, and options for future control

**DOI:** 10.1186/s12916-016-0665-8

**Published:** 2016-09-01

**Authors:** Yoon Hong Choi, Helen Campbell, Gayatri Amirthalingam, Albert Jan van Hoek, Elizabeth Miller

**Affiliations:** 1 Immunisation, Hepatitis and Blood Safety Department, National Infection Service, Public Health England, 61, Colindale Avenue, London, NW9 5EQ UK; 2 Department of Infectious Disease Epidemiology, London School of Hygiene and Tropical Medicine, Keppel Street, London, WC1E 7HT UK

**Keywords:** Pertussis, Vaccine, Resurgence, Mathematical model, Transmission, Intervention

## Abstract

**Background:**

In 2012 England and Wales experienced a resurgence of pertussis and an increase in infant deaths. This occurred 8 years after acellular pertussis (aP) vaccine replaced whole cell (wP) primary vaccine despite continued high coverage for the primary series and pre-school aP booster. We developed a mathematical model to describe pertussis transmission dynamics in England and Wales since the 1950s and used it to investigate the cause of the resurgence and the potential impact of additional vaccination strategies.

**Methods:**

An age-structured, compartmental, deterministic model of the pertussis transmission dynamics was fitted to 60 continuous years of age-stratified pertussis notification data in England and Wales. The model incorporated vaccine-induced and natural immunity and differentiated between vaccine-induced protection against clinical disease and infection.

**Results:**

The degree of protection of wP vaccine against infection was estimated to be higher than that of aP vaccine. Furthermore, the duration of protection for natural and wP-induced immunity was likely to be at least 15 years, but for aP vaccine it could be as low as 5 years. Model results indicated that the likely cause of the resurgence was the replacement of wP by less efficacious aP vaccine and that an elevated level of pertussis would continue. The collapse in wP vaccine coverage in the 1970s and resultant outbreaks in the late 1970s and early 1980s could not explain the resurgence. Addition of an adolescent or toddler booster was predicted to have little impact on the disease in infants.

**Conclusions:**

Our findings support the recent recommendation by the World Health Organisation that countries currently using wP vaccine for primary immunisation should not change to aP vaccine unless additional strategies to control infant disease such as maternal immunisation can be assured. Improved pertussis vaccines that provide better protection against infection are needed.

**Electronic supplementary material:**

The online version of this article (doi:10.1186/s12916-016-0665-8) contains supplementary material, which is available to authorized users.

## Background

England and Wales, in common with several other countries, recently experienced a resurgence of pertussis despite sustained high vaccine coverage. A recent review of pertussis epidemiology in countries with and without a resurgence by the World Health Organisation (WHO) Strategic Advisory Group of Experts (SAGE) found that, while there was no evidence of a global resurgence in disease, the countries with clear evidence of a resurgence had all replaced whole cell pertussis (wP) with acellular pertussis (aP) vaccines 6–9 years previously [1].

**Fig. 1:**
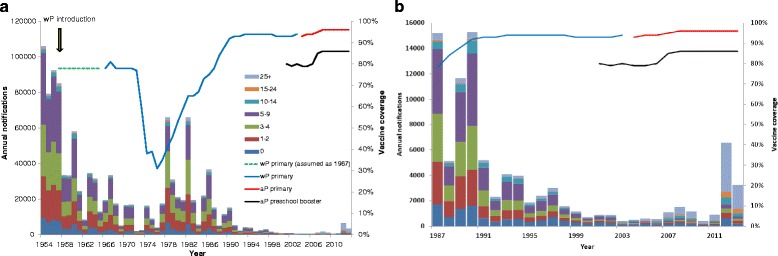
Number of annual notifications (**a**) between 1954 and 2013 and (**b**) between 1986 and 2013 in England and Wales in seven age groups, and the historical vaccine coverage (course completed) for the primary and pre-school booster programmes (*blue line*: wP primary vaccine coverage, *red line*: aP primary vaccine coverage, *black line*: aP pre-school booster vaccine coverage). Coverage data for 1957 to 1966 not collected but assumed to be the same as in 1967 as it was added into an existing DT vaccine (available to download in Additional file 1)

Prior to the introduction of wP vaccine in England and Wales in 1957, around 100,000 cases of pertussis were reported annually. The wP vaccination programme successfully reduced pertussis incidence until vaccine coverage collapsed in the 1970s and 1980s following publications that suggested a possible link between wP vaccine and brain damage (Fig. 1a and Additional file 1). This fall in coverage led to large pertussis outbreaks in the late 1970s and 1980s [2]. Vaccine coverage then increased steadily as public and professional confidence in the vaccine was restored, reaching 92 % in 1992 and remaining high thereafter (Fig. 1b). Despite sustained high coverage from the early 1990s, pertussis among infants younger than 3 months who were too young to be vaccinated still caused around 270 hospitalisations per 100,000 up to year 2000 [3]. In 2001, a booster dose of a three- or five-component aP-containing vaccine given to children between 3 ½ and 5 years of age was introduced in the United Kingdom (UK) to reduce transmission among older children and consequently to infants [2, 4]. No other boosters are currently given in the UK. In 2004, the primary wP vaccine given at 2, 3 and 4 months of age [2] was replaced with aP vaccine, prompted by the decision to change from oral to inactivated polio vaccine (IPV) for which the only available IPV combination vaccine was one containing an aP component [2, 5]. The aP vaccine used exclusively in the UK until June 2014 was a five-component product (Pediacel®, Sanofi Pasteur MSD) containing pertussis toxin, filamentous haemagglutinin, pertactin and fimbrial antigens 2 and 3.

The pertussis resurgence that occurred in England and Wales in 2012 resulted in an increase in deaths and hospitalisations among infants too young to have received their first dose of vaccine under the primary schedule. To provide immediate passive protection to vulnerable infants, a maternal pertussis immunisation programme with an aP-containing vaccine was introduced in 2012 as a temporary outbreak control measure. The programme successfully reduced pertussis morbidity and mortality among infants in England [4], but its long-term continuation will depend on whether the elevated levels of pertussis seen in the resurgence are likely to continue. We developed mathematical models to describe the pertussis transmission dynamics in England and Wales and to explore the following: the likely cause of the resurgence, in particular whether the change from wP to aP vaccine or the decline in vaccine coverage in the 1970s and 1980s contributed to the resurgence; whether the higher incidence of disease in infants can be expected to continue; and the impact of the addition of a toddler or adolescent booster to the existing programme.

## Methods

We developed an age-structured, compartmental deterministic model to describe pertussis transmission dynamics and the impact of vaccination. We distinguish between natural and vaccine-induced immunity and between protection against clinical disease and infection. As suggested by the baboon challenge studies of Warfel et al. [6, 7], we assumed that natural and vaccine-induced immunity completely protect against clinically typical (i.e. notifiable) pertussis symptoms, but that vaccination only offers partial protection against acquiring and transmitting infection, the degree potentially varying between wP and aP vaccines. We also assumed that both natural and vaccine-induced immunity wane with time and that, if re-infected, an individual has a lower probability of developing notifiable disease. The waning parameters for natural, wP and aP protection against disease, the degree of protection from aP and wP vaccines against infection and the probability of being notified with a first compared with a second infection were estimated by fitting the model to the age-specific notification data for England and Wales shown in Fig. 1.

### Model structure

The assumptions for unvaccinated individuals are as follows: all newborn infants are susceptible (S1); individuals in S1 can become infected and enter the latent (non-infectious) phase (L1) according to the age-group-dependent force of infection (FOI, λ). The FOI for the *i*th age group, ${\lambda}_i$, is calculated as follows:

$$ {\lambda}_i(t)={\displaystyle {\sum}_{j=1}^9{I}_j(t)\times {C}_{ij}\times {q}_j,} $$

where ${I}_j(t)$ is the sum of infectious individuals in the *j*th age group at time *t*, ${C}_{ij}$ is the contact rate of the *i*th and *j*th age groups and ${q}_j$ is the transmission probability per contact in the *j*th age group.

After the latent period (1/σ), individuals become infectious (I1) with a proportion (α1) of them developing notifiable symptoms. At the end of the infectious period (1/ γ), individuals gain natural immunity (R) against disease and infection; this immunity then wanes (waning rate, ω) with individuals becoming secondary susceptibles (S2). An individual in S2 can become infected again according to the same FOI, λ, as an individual in S1, but the proportion developing notifiable symptoms, α2, is less than α1. The durations of the latent (L2) and infectious periods (I2) in a secondary infection are the same as for L1 and I1 and were assumed to be 7 and 15 days respectively [8–10]. After clearing infection, individuals in I2 gain natural immunity (R).

The assumptions for vaccinated individuals are as follows: on vaccination, individuals in S1 move to the corresponding acellular (aP) or whole cell (wP) protected compartments depending on which vaccine they receive. Vaccine-protected individuals can still be susceptible to infection, though with a lesser FOI than individuals in S1; therefore, these compartments are denoted as wPS or aPS. This reduced FOI reflects some degree of protection against infection (π) and can differ between aP and wP vaccines (π_ap_ and π_wp_ respectively). The FOI acting on individuals in the aPS and wPS compartments is therefore λ(1− π). After clearing the infection, vaccine-protected individuals acquire natural immunity and enter the R compartment. The vaccine-induced protection against notifiable symptoms and the partial protection against infection wane in parallel with each other, with the duration potentially differing between aP and wP vaccinated individuals (1/*aPω* and 1/*wPω* respectively). Individuals in whom vaccine-induced immunity has waned enter the S2 compartment. An aP booster, whether given in the second year of life, at pre-school age or in adolescence, has an effect on individuals in S1 and S2, and moves them to aPS, where it was assumed that the degree of protection against infection (π) and the waning function (*aPω*) were the same as for a primary aP vaccinee and independent of whether aP or wP vaccine was given for priming. The third dose in the three-dose primary series is assumed to be given at 4 months, at which age vaccinated infants enter the wPS or aPS compartments.

**Fig. 2:**
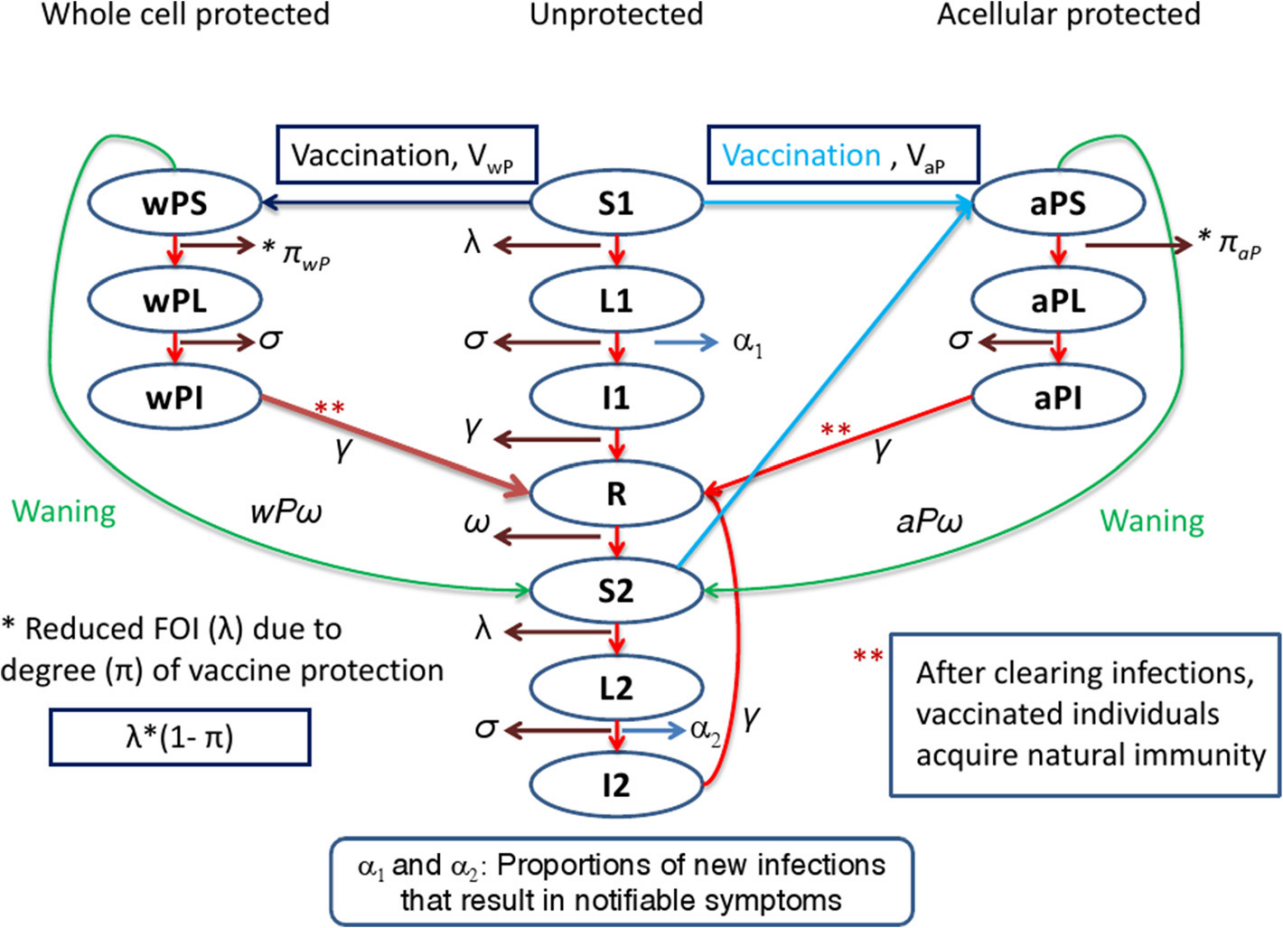
Flowchart of the pertussis transmission dynamic model with acellular and whole cell vaccine-protected groups. (The difference equations describing the flows between compartments are given in Appendix 1 in Additional file 2)

The flowchart in Fig. 2 shows the transitions between model compartments for vaccine-protected and unprotected individuals.

### Data sources

Notifications of clinically suspected cases of pertussis are the only data source that pre-dates the introduction of wP vaccination and are still recorded. From 1950 onwards, notification data were available in the seven age groups (or finer ones) used in the model and were published in the Annual Reviews from the Registrar General [11] and from 1972 to 1981 by the Office of Population Censuses and Surveys [12] and thereafter as part of the Notifications of Infectious Diseases (NOIDs) data set [13]. The notification incidence in 1956 was assumed to be the pre-vaccination equilibrium year, as it was close to the average of the three-year pre-vaccination period 1954–1956.

Vaccine coverage data for the primary series were only available from 1966, so the assumption was made that the coverage between 1957 and 1965 was the same as in 1966 (Fig. 1a), as the wP component was included in the existing DT (diphtheria vaccine introduced in 1942 and tetanus component added in 1950) primary vaccine. Coverage for the pre-school booster introduced in 2001 was generally about 10 % lower than the primary schedule [4, 14–16].

The mixing patterns between and within age groups are critical determinants of the age-dependent FOIs. The only available data on mixing patterns across all age groups were collected via the POLYMOD contact survey carried out in England in 2006 [17]. The POLYMOD contact information for infants was sparse and so was supplemented by data from a recent study in England that documented the number and type of contacts experienced by infants younger than 1 year of age [18]. The annual population changes and POLYMOD contact patterns adjusted for changes in population structure between 1956 and 2030 are shown in Appendix 2 (Movies A.1 and A.2) in Additional file 2.

### Model fitting

As little is known about the transmission of pertussis, it was decided to model pertussis with as few assumptions as possible. Only the FOIs and α1 were fitted in order to match the model output with the observed age-stratified notification data from 1956. Other parameters, i.e. α2, duration of natural immunity, the duration of wP- and aP-induced immunity and the degree of wP and aP protection against infection were explored by running the model with the given assumptions in Table 1. For this, α2 was assumed to be lower than α1; hence scenarios for α2 are expressed as a percentage of α1. Furthermore, it was assumed that the duration of protection induced by wP could not be longer than the duration of natural protection, and the duration of protection induced by aP could not be longer than that of wP. The minimum average duration of 5 years for aP protection was based on epidemiological data from England that showed no decline in aP vaccine effectiveness in the period between completion of the primary series and receipt of the pre-school booster [4]. Exploration of the possible disease scenarios was divided hierarchically in three steps: first the pre-vaccination era (1956), the wP era (1957–2000) and subsequently the aP era (2001–2013, including the aP booster introduced in 2001 and the replacement of the wP primary vaccine with aP vaccine in 2004).

**Table 1:** Model parameters varied in the fitting and simulation procedures to cover their uncertainty and the values varied for each parameter

Parameter	Values varied
Average duration of natural immunity (ω)	10, 15, 20, 25, 30, 35, 40, 45 and 50 years
Scale parameter for the proportion of second + infections that are notified compared to proportion of first infections in unvaccinated individuals (α2/α1)	10 % (1/10), 5 % (1/20), 3.3 % (1/30), 2.5 % (1/40) and 2 % (1/50)
Degree of wP and aP vaccine protection against infection (π_wp_ and π_ap_)	10, 20, 30, 40, 50, 60, 70, 80 and 90 %
Average duration of wP and aP vaccine protection against infection and disease (wPω and aPω)	5, 7.5, 10, 12.5, 15, 17.5, 20, 22.5, 25, 27.5 and 30 years

For the pre-vaccination period, the FOIs by age (nine age groups) and α1 were fitted, using a static model, given each of the combinations for the duration of natural infection (9 values from 10 to 50 years [19, 20]) and α2/α1 (5 values, from 2–10 %). This resulted in 45 parameterised models which each reproduced the 1956 age-stratified notification data. For clarity, the FOIs were used to set the age-specific transmission probabilities per contact (9 values) in combination with the POLYMOD contact matrix as adjusted to the 1956 population (Appendix 2 in Additional file 2).

These 45 obtained scenarios were subsequently applied to the model in the wP era. Each of the 45 scenarios was combined with the historical vaccine uptake and with all possible combinations of degree (9 values) and duration of protection of wP (11 values) with the restriction that the duration of protection by wP could not be longer than the duration of the natural immunity. The best 5 % fitting scenarios (178 scenarios) among 3555 simulated scenarios were selected based on the Poisson deviance of the model outputs to the historical notification data (1956–2000).

For the aP era the obtained 178 scenarios were combined with the uptake of aP and possible combinations of degree (9 values) and duration of protection (11 values). This time the duration of aP vaccine protection could not be longer than that of wP [19–21]. Again, the 5 % best fitting (658) scenarios were selected among 13,158 simulated scenarios based on the Poisson deviance of the model outputs to the historical notification data (2001–2013). The obtained set of 658 scenarios was used in projecting the future incidence.

### Sensitivity analysis of the contact matrix

There are no separate sensitivity analyses of individual model parameters apart from the mixing matrix, as model outputs were generated with combinations of possible parameter values within the potential ranges defined in Table 1.

The POLYMOD mixing matrices were derived from population surveys conducted in 2006 [17] and 2012 [18]; therefore, the data may not be appropriate to reflect the contact patterns up to 50 years earlier, even after adjusting the mixing matrix with annual population changes. Hence, we also ran the model using a classical assortative mixing approach based on two extreme mixing matrices, one that assumes mixing is exclusively within the same age group (fully assortative) and one that assumes equal mixing within and between age groups (fully proportionate). The mixing parameter between the two extreme mixing matrices was estimated by fitting to the post-vaccination data. By comparing the model outputs using the classic assortative mixing approach, the robustness of the results generated with the POLYMOD mixing approach was assessed (Appendix 3 in Additional file 2).

## Results

### Fitting

**Fig. 3:**
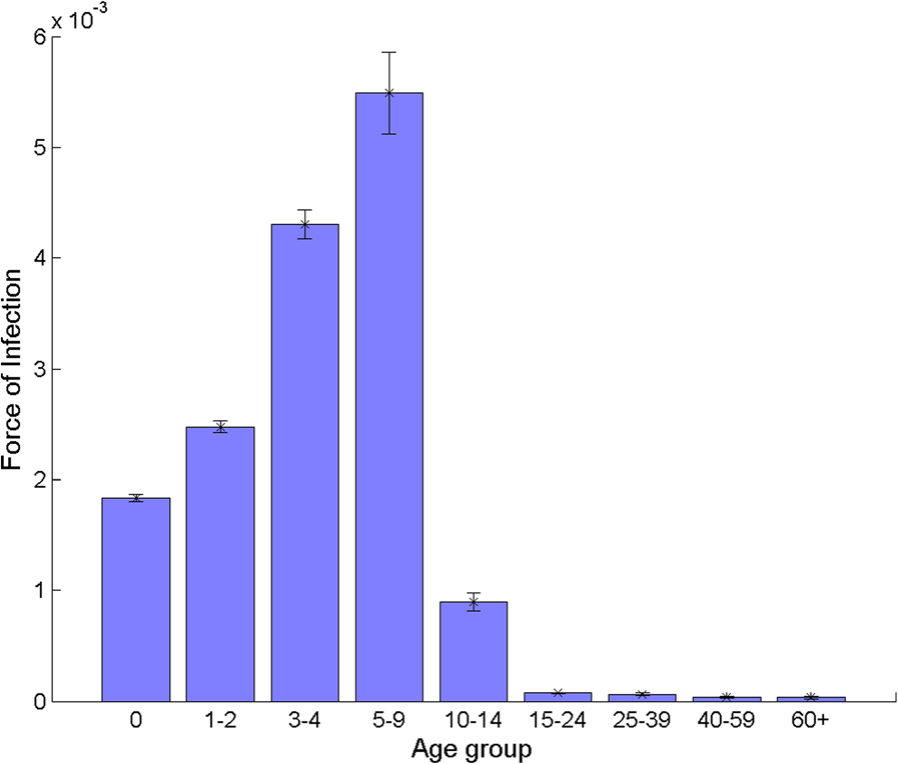
Mean, maximum and minimum values of the force of infection by nine age groups derived from the 1956 pre-vaccination equilibrium in England and Wales (among 45 combined scenarios with 9 different durations of natural immunity and 5 different values of α2/α1)

The proportion of first infections that are notified (α1) from these selected scenarios was estimated to be between 13.6–14.7 %. Figure 3 presents the fitted mean, maximum and minimum values of FOIs by age group at the 1956 equilibrium year. The lower forces of infection in 15+ age groups led to lower transmission probabilities per contact, as shown in Appendix 2 in Additional file 2.

**Fig. 4:**
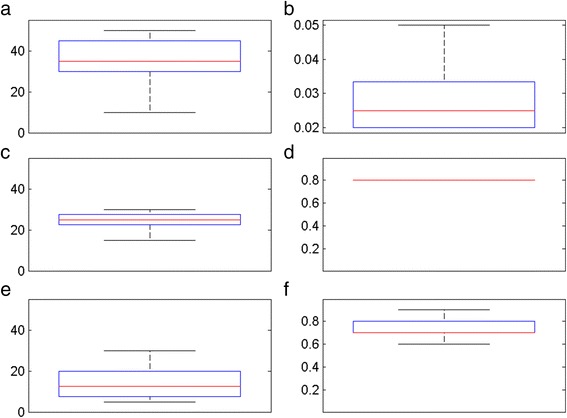
Box plots of parameter estimates from the 2000 best fitting aP scenarios to historical notification data between 1956 and 2013 in England and Wales. **a** Duration of natural immunity; **b** α2/α1; **c** duration of wP vaccine protection; **d** degree of whole cell vaccine protection against infection; **e** duration of aP vaccine protection; **f** degree of acellular vaccine protection against infection. *Red line* for median, *blue box* for lower and upper quartiles and *black bars* for minimum and maximum

The parameter values for α2/α1, average duration of natural and vaccine-induced protection (wP and aP) and degree of protection (wP and aP) against infections for the selected best fitting 658 scenarios (5 % of all simulated scenarios) are shown in Fig. 4. The value of α2/α1 was estimated to be 5 % (1/20) or lower, implying that secondary infections have much less chance of developing notifiable symptoms than first infections in an unvaccinated individual. Primary infections are therefore the main driver of pertussis notification rates.

For wP vaccine, only scenarios with 80 % degree of protection against infection were among the 5 % best fitting scenarios, implying that wP vaccine has produced a strong herd immunity during the wP post-vaccination era. On the other hand, the aP vaccine scenarios selected had a lower degree of protection against infection (and thus a lesser herd protection) than wP, between 40–90 %, with the values between the lower (second) and upper (third) quartiles ranging from 50–80 %. For the duration of wP protection against infection, scenarios with a period between 20 and 27.5 years were selected between the lower and upper quartiles, while the aP produced between 7.5 and 17.5 years for the same quartiles (Fig. 4). Fitting results indicated a strong inverse correlation between the duration and degree of aP vaccine protection against infection (high degree paired to a low duration and vice versa). For wP vaccine, such a correlation could not be demonstrated, due to the single value for the degree of protection against infection (80 %).

**Fig. 5:**
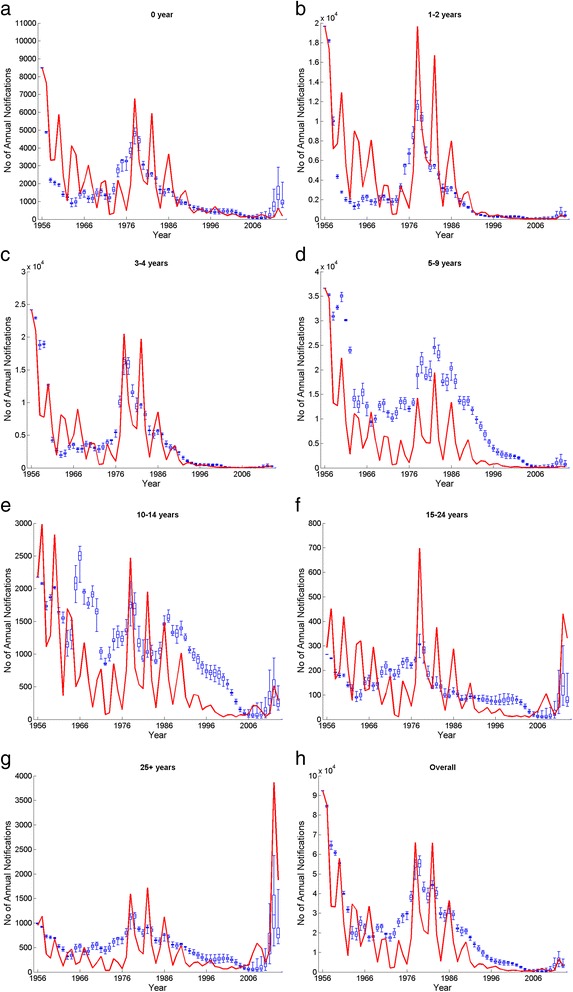
Notification data (*red line*) between 1956 and 2013 in England and Wales and corresponding model outputs from best fitted scenarios (*blue box plots* minimum, lower quartile, median, upper quartile and maximum) by seven age groups (**a**–**g**) and the overall population (**h**)

The model outputs by age group using the best fitting scenarios and under the current vaccination programme are shown in Fig. 5. Even though the fluctuations shown in the data points were not precisely produced in the model outputs, overall trends in number of notifications were generally well reflected within age groups, particularly during the resurgences in the 1970s and 1980s and in 2012. Similar parameter estimation results from the assortative mixing approach were obtained and are presented in Appendix 3 in Additional file 2.

### Simulation results for alternative vaccination strategies

**Fig. 6:**
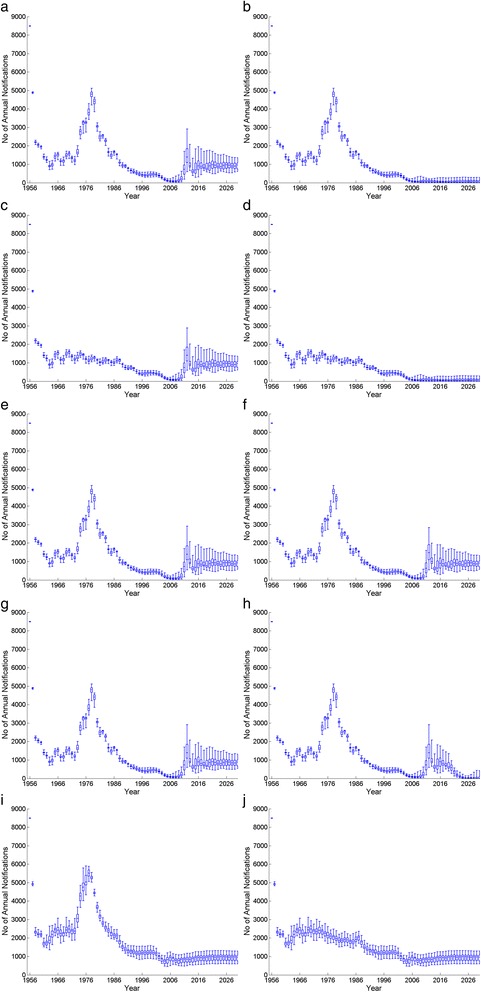
Model outputs of the annual pertussis notification among infants less than a year old between 1956 and 2030 in England and Wales with the ten different vaccination programme options as shown in Table 2 (excludes 6B, as it shows similar results to those of 6A) and *blue box plots* showing minimum, lower quartile, median, upper quartile and maximum of the annual pertussis notifications from the best fitting scenarios)

Using the best fit parameter estimates, we investigated the impact of ten vaccination programme options as listed in Table 2 on the number of pertussis notifications in infants less than 1 year of age for the period 1956 to 2030 (Fig. 6); the potential impact of continuing the maternal pertussis vaccination was not included in these simulations.

**Table 2:** List of ten different vaccination programme options for the long-term simulations between 1956 and 2030 in England and Wales

Option	Long-term vaccination programme
1	Continuation of the current strategy: wP primary from 1957 + aP pre-school booster in 2001 + change to aP primary in 2004 but without the maternal immunisation programme that began in October 2012
2	wP primary used throughout with aP pre-school booster in 2001
3	Opt. 1 + without vaccine coverage drop in 1970s and 1980s
4	Opt. 2 + without vaccine coverage drop in 1970s and 1980s
5	Opt. 1 + aP booster at the age of 13 years in 2015
6	Opt. 1 + aP booster at 12 (6A) or 18 months (6B) in 2015
7	Opt. 1 + aP booster at 12 months and at 13 years in 2015
8	Opt. 1 + aP vaccine replacement with wP-like vaccine in 2015
9	aP primary vaccine from 1957 + aP pre-school booster in 2001
10	Opt. 9 without the vaccine coverage drop in 1970s and 1980s

These ten vaccination programme options were chosen to investigate the potential causes of the resurgence, and to explore the effect of alternative vaccination strategies. Options 1 and 2 were simulated to investigate the impact of replacing wP with aP in 2004. Options 3 and 4 were simulated to investigate whether the collapse in vaccine coverage in the 1970s and 1980s led to the resurgence by assuming that coverage remained at 78 % between 1970 and 1986. Options 5 and 6 assessed respectively the impact of an adolescent aP booster at 13 years old (5) and/or a toddler aP booster at 12 months (Option 6A) or 18 months (Option 6B) of age added to the current strategy in 2015. In particular, Option 7 was used to investigate the impact of introducing both toddler (at 12 months) and adolescent (at 13 years old) boosters in the same year (2015). Option 8 investigated the impact of introducing a vaccine with the same degree and duration of protection against infection as wP in 2015. Options 9 and 10 investigated the likely long-term impact if aP vaccine had been introduced as the primary pertussis vaccine from 1957 with or without the vaccine coverage collapse in the 1970s and 1980s.

If wP had been retained for primary vaccination, with addition of the aP pre-school booster in 2001, the model predicts that there would be no resurgence, irrespective of the drop in vaccine coverage in the 1970s and 1980s (Options 2 and 4 compared with Options 1 and 3). Introduction of an adolescent and/or a toddler booster at 12 months in 2015 was predicted to have little impact on infant disease (Options 5, 6A and 7). Option 8, introducing a new vaccine with a higher degree or duration of protection than aP vaccine, could in the longer term lower the incidence of infant pertussis to the level before the change to aP vaccine in 2004. If aP vaccine had been used since 1956, the model predicts that, with or without the drop in vaccine coverage in the 1970s and 1980s, there would not have been a resurgence in 2012, though the incidence in infants would have equilibrated at a level higher than observed throughout the period (Options 9 and 10).

Simulation results from the assortative mixing approach were almost identical to those of the POLYMOD mixing approach as presented in Appendix 3 in Additional file 2. The impact of the ten simulated programme options in Table 2 using both POLYMOD and assortative mixing approaches for the remaining six age groups and the overall population can be seen in the Graphical User Interface programme (available to download in Additional file 3).

## Discussion

Our simulation results indicate that the likely cause of the resurgence in pertussis in England and Wales in 2012 was the change in 2004 to an aP vaccine that provided a lesser degree and/or shorter duration of protection against infection than the previously used wP vaccine. Although there was a collapse in vaccine coverage in the 1970s and 1980s, simulation results did not suggest that this was a causal factor in the resurgence. Our results also indicate that a continued higher incidence of pertussis in infants and older age groups can be expected in the future and suggest that the resurgence that began in 2012 reflects the resetting of incidence at a higher level when moving from the post-wP vaccine equilibrium to the post-aP vaccine equilibrium.

Our results apply to the five-component aP vaccine, which was used exclusively in the UK until 2014, but other aP vaccines with fewer components are unlikely to provide higher or more durable protection against infection. The wP vaccines used in the UK have varied over the years, but all have shown consistently high protection in post-licensure studies after the potency criteria were standardised in 1968 [2]. However, even with high coverage using a good wP vaccine, and with an aP pre-school booster, the average annual admission rate in infants younger than 3 months in England and Wales immediately before the change to aP vaccine was still high at 156 per 100,000 and with an annual mortality rate of 7.2 per million maternities [4]. This indicates the need for improved pertussis vaccines that could more effectively control pertussis transmission by inducing durable sterilizing immunity.

There is considerable uncertainty about the epidemiology of pertussis, posing problems for the building and fitting of realistic mathematical models of pertussis transmission dynamics. Atypical or mild infections can occur in older age groups, probably associated with waning immunity. There is however little data on their frequency or role in transmission, though there are well-documented instances of adults with a history of vaccination who developed a mild cough subsequently confirmed as pertussis transmitting to infants [22], and parents with an undiagnosed cough illness have been shown to be the source of infection in around half of infant cases [23, 24]. In our model, we took account of this by assuming that those who had been infected or vaccinated had less chance of developing notifiable pertussis symptoms if re-infected but could still transmit. Furthermore, we observed a low force of infection in those aged 15+, suggesting a relatively small contribution of these age groups to the wider transmission. We also differentiated between protection against infection and clinical disease. This was based on the baboon challenge model [7], which showed that while both aP and wP vaccines and natural immunity provided complete protection against clinical pertussis, aP vaccinated baboons were colonised for up to 35 days and still infectious though asymptomatic; wP vaccinated baboons were infected for shorter periods than aP vaccinees, whereas convalescent baboons developed sterilizing immunity on re-challenge [7]. In our simulations, the degree of protection of wP against infection was estimated to be at least 80 %, which accords with the estimate of 85 % from a household contact study in Senegal for one of the wP vaccines that has been used in the national programme in the UK [25]. In contrast, the degree of protection for aP could be as low as 60 % (Fig. 4). For duration of protection, the best fitting wP scenarios were mostly with durations of at least 15 years, similar to that of natural immunity, and are consistent with estimates from epidemiological studies [19]. Due to the relatively short time aP vaccines have been used in England and Wales, our model could not distinguish well between different values for the duration of aP protection, although, unlike wP vaccine, durations as short as 5 years were plausible (Fig. 4). The lower degree and/or shorter duration of aP vaccine protection against infection would result in reduced herd immunity compared with wP vaccine and would increase transmission in the population, resulting in a resurgence. Epidemiological studies in Australia and the USA — aP-using countries where pertussis resurgences have also occurred — suggest a shorter duration of protection after priming with aP than wP vaccines [26, 27] and rapid waning of protection after a pre-school or adolescent booster dose [28–32].

Others have developed models to investigate the likely cause of the recent resurgences of pertussis. Gambhir et al. [21] developed a model fitted to 20 years of age-stratified notified incidence data in the USA that allowed for waning immunity and re-infection and different durations of natural, wP- and aP-induced immunity. They concluded that the US resurgence was most likely caused by lower efficacy and duration of protection from aP than wP vaccine. Campbell et al. [33] developed an Australian model based on serological data and the ability to reproduce broad epidemiological trends such as the inter-epidemic period. The latter required an intermediate stage of waned immunity, when boosting without infectiousness occurs, before transition to a low state, when boosting results in infectiousness. The duration of this intermediate state was estimated to be considerably longer after natural than vaccine-induced immunity, though with little difference between aP and wP vaccines. However, the model did not distinguish much difference between aP and wP vaccines, and it was concluded that the replacement of the aP toddler with an adolescent booster dose in 2004 contributed to the subsequent resurgence in 2008. Althouse and Scarpino [34] used models to analyse pertussis notification and genetic data from the USA and England and Wales and concluded that asymptomatic transmission in the acellular vaccine era was the most parsimonious explanation of the recent resurgences. However, their compartment of asymptomatic infection is operationally similar to our assumption that only a proportion of infections is notified (i.e. the parameters α1 and α2) and is not necessarily an alternative explanation to waning protection in models that distinguish between protection against notifiable disease and protection against infection.

Use of notifications data for model fitting is hampered by possible changes in surveillance sensitivity over time, as reporting of clinical cases may become more complete when new diagnostic methods are developed. Without independent information on the likely magnitude of temporal changes in surveillance sensitivity within and between age groups, it is difficult to accommodate this in the model fitting. For example, in older age groups the introduction of serological testing in 2001 in England and Wales is likely to have improved reporting of cases in adults; however, this likely increase in completeness of notifications was not taken into account. Furthermore, we did not include maternal immunisation, introduced in 2012, in our model. However, due to the timing of introduction, this would only have had an effect on the disparity between observed and predicted notifications in 2013.

## Conclusions

Despite the inevitable limitations in developing and parameterising a realistic pertussis dynamic transmission model based on a long historical data set, our simulations provide valuable insights into the likely cause of the resurgence in England and Wales and of the relative merits of different control strategies. With the elevated incidence of infant disease predicted to continue in the absence of a maternal immunisation programme, there will be a continuing need for this programme to protect vulnerable infants. Our model predicts that introduction of an adolescent booster would have little impact on infant disease, despite our favourable and probably unrealistic assumption that it provides a similar duration of protection as achieved with the three-dose primary infant course [25–29]. However, an adolescent booster may be justified in terms of the direct protection afforded to this age group who, while at very low risk of hospital admission and death, can have a distressing and prolonged cough that interferes with their sleep and daily activity [35]. Our model provides a quantitative basis for conducting cost-effectiveness analyses of adding pertussis vaccine to the combined tetanus/low-dose diphtheria/inactivated polio booster vaccine currently offered to this age group in England and Wales. Our findings support the recent recommendation by the World Health Organisation that countries currently using wP vaccine for primary immunisation should continue to use wP vaccines, and a switch from wP to aP should only be considered when additional strategies to control infant disease such as maternal immunisation can be assured [36].

## Abbreviations

aP, acellular pertussis vaccine; DT, diphtheria and tetanus vaccine; FOI, force Of infection; IPV, inactivated polio vaccine; NOIDs, Notifications of Infectious Diseases; SAGE, Strategic Advisory Group of Experts; UK, United Kingdom; WHO, World Health Organisation; wP, whole cell pertussis vaccine

## Additional files

Additional file 1: Pertussis notification data and pertussis vaccination coverage between 1954 and 2013 in England and Wales in an EXCEL file. (XLSX 12 kb) Additional file 2: Appendix. (DOCX 8095 kb) Additional file 3: Graphical User Interface porgramme to present pertussis simulation model results. (ZIP 8235 kb)
