# Supplementary material for: Investigating the pertussis resurgence in England and Wales, and options for future control
Source: BMC Med. 2016 Sep 1;14(1):121. doi: 10.1186/s12916-016-0665-8 (PMC5007864; doi:10.1186/s12916-016-0665-8)
Supplement: Additional file 2: — Appendix. (DOCX 8095 kb) [file 12916_2016_665_MOESM2_ESM.docx]

**Appendix**

1. **Model equations**

$${S1}_{i}\left( t \right)=\left( 1-\lambda_{i}\left( t-1 \right)-V_{wPi}\left( t \right)-V_{aPi}\left( t \right) \right){S1}_{i}\left( t-1 \right),$$

$${L1}_{i}\left( t \right)=\left( 1-\sigma\right){L1}_{i}\left( t-1 \right)+\lambda_{i}{\left( t-1 \right)S1}_{i}\left( t-1 \right),$$

$${I1}_{i}\left( t \right)=\left( 1-\gamma\right){I1}_{i}\left( t-1 \right)+\sigma{L1}_{i}\left( t-1 \right),$$

$$R_{i}\left( t \right)=\left( 1-\omega\right)R_{i}\left( t-1 \right)+\gamma\left( {I1}_{i}\left( t-1 \right)+{I2}_{i}\left( t-1 \right)+{wPI}_{i}\left( t-1 \right)+{aPI}_{i}\left( t-1 \right), \right)$$

$${S2}_{i}\left( t \right)=\left( 1-\lambda_{i}\left( t-1 \right)-V_{aPi}(t) \right){S2}_{i}\left( t-1 \right)+\omega R_{i}\left( t-1 \right)+wP\omega\times{wPS}_{i}\left( t-1 \right)+aP\omega\times{aPS}_{i}\left( t-1 \right),$$

$${L2}_{i}\left( t \right)=\left( 1-\sigma\right){L2}_{i}\left( t-1 \right)+\lambda_{i}{\left( t-1 \right)S2}_{i}\left( t-1 \right),$$

$${I2}_{i}\left( t \right)=\left( 1-\gamma\right){I2}_{i}\left( t-1 \right)+\sigma{L2}_{i}\left( t-1 \right),$$

$$wPS_{i}\left( t \right)=\left( 1-{\left( 1-\pi_{wP} \right)\lambda}_{i}\left( t-1 \right)-wP\omega\right){wPS}_{i}\left( t-1 \right)+V_{wPi}\left( t \right){S1}_{i}\left( t-1 \right),$$

$${wPL}_{i}\left( t \right)=\left( 1-\sigma\right)wPL_{i}\left( t-1 \right)+{\left( 1-\pi_{wP} \right)\lambda}_{i}\left( t-1 \right)wPS_{i}\left( t-1 \right),$$

$${wPI}_{i}\left( t \right)=\left( 1-\gamma\right)wPI_{i}\left( t-1 \right)+\sigma{wPL}_{i}\left( t-1 \right),$$

$$aPS_{i}\left( t \right)=\left( 1-{\left( 1-\pi_{aP} \right)\lambda}_{i}\left( t-1 \right)-aP\omega\right){aPS}_{i}\left( t-1 \right)+V_{aPi}\left( t \right)\{{S1}_{i}\left( t-1 \right)+{S2}_{i}\left( t-1 \right)\},$$

$${aPL}_{i}\left( t \right)=\left( 1-\sigma\right)aPL_{i}\left( t-1 \right)+\left( 1-\pi_{aP} \right)\lambda_{i}\left( t-1 \right)aPS_{i}\left( t-1 \right),$$

$${aPI}_{i}\left( t \right)=\left( 1-\gamma\right)aPI_{i}\left( t-1 \right)+\sigma{aPL}_{i}\left( t-1 \right),$$

where $i=1,2,\ldots,5200$ for each weekly age cohort, $\lambda_{i}$ is the age-dependent force of infection, 1/σ is the latent period, $1/\gamma$ is the average duration of the infectious period, $1/\omega$ is the average duration of natural immunity, $\mathrm{wP}\omega$ and $\mathrm{aP}\omega$ are the vaccine waning rates for wP and aP vaccines respectively, and $\pi_{\mathrm{wP}}$ and $\pi_{\mathrm{aP}}$ are respectively the degree of wP and aP vaccine protection against infection.

The number of notifications at each time step is calculated as follows:

$Notifications\left( t \right)=\sum_{i=1}^{5200} \left( \alpha_{1}\left( \lambda_{i}{\left( t-1 \right)S1}_{i}\left( t-1 \right) \right)+\alpha_{2}\left( \lambda_{i}{\left( t-1 \right)S2}_{i}\left( t-1 \right) \right) \right)$.

Figure A.1. Differential equations of the pertussis transmission dynamics described in Figure 2 in the main paper

1. **Annual population structures and POLYMOD contact mixing matrices**

The annual population changes in England and Wales between 1956 and 2030 are presented as a video clip in Movie A.1.

Movie A.1. Movie clip for the population sizes of nine age groups between 1956 and 2030 in England and Wales (The population information between 2014 and 2030 are the predictions estimated from the Office of National Statistics (Office for National Statistics, n.d.)).

The annual adjusted POLYMOD contact matrices using the population changes in England and Wales between 1956 and 2030 are presented as a video clip in Movie A.2.

Movie A.2. Movie clip for the annual POLYMOD contact patterns between age groups adjusted with the population data between 1956 and 2030 in England and Wales (The population information between 2014 and 2030 are the predictions estimated from the Office of National Statistics.) (the contour presents the contact probability per person per day between age groups).

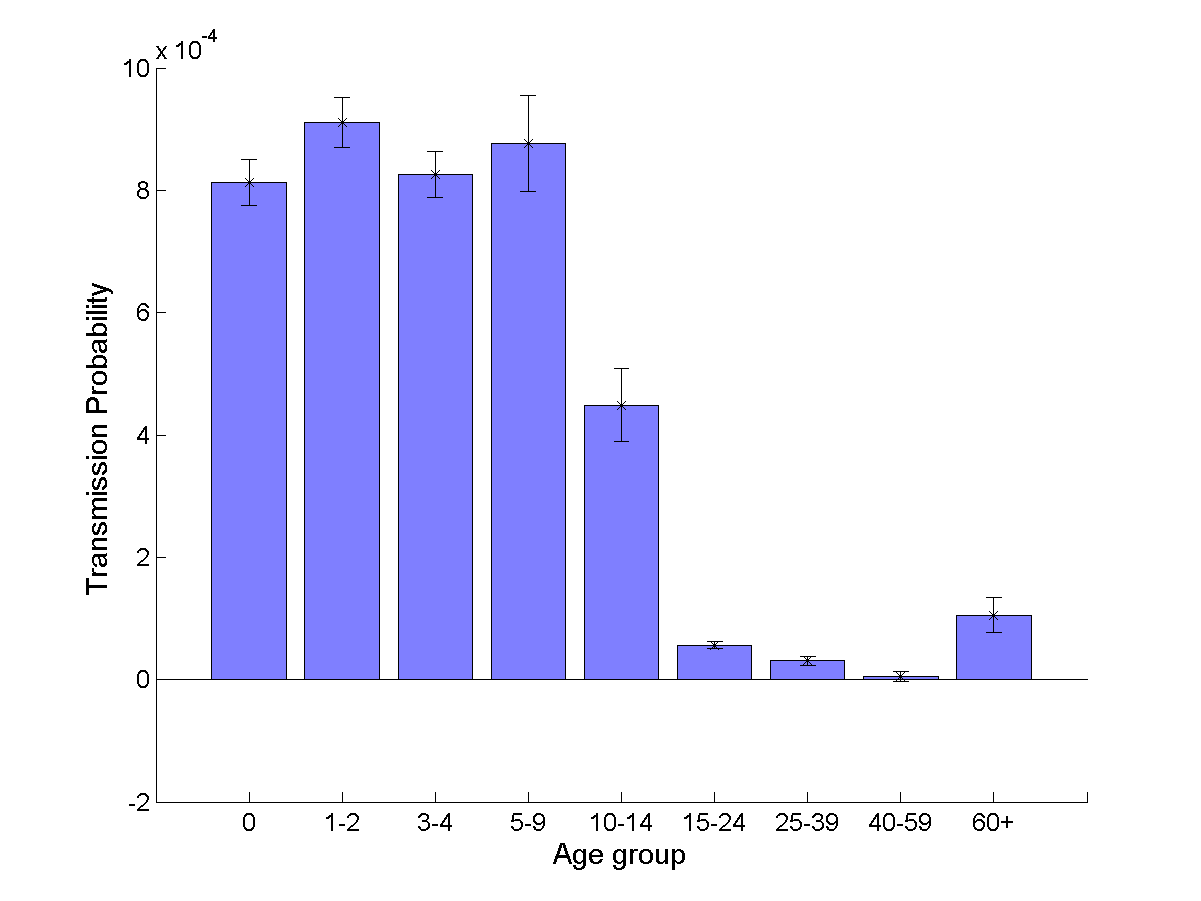


Figure A.2. Pertussis transmission probabilities per contact by age groups estimated at 1956 in England and Wales.


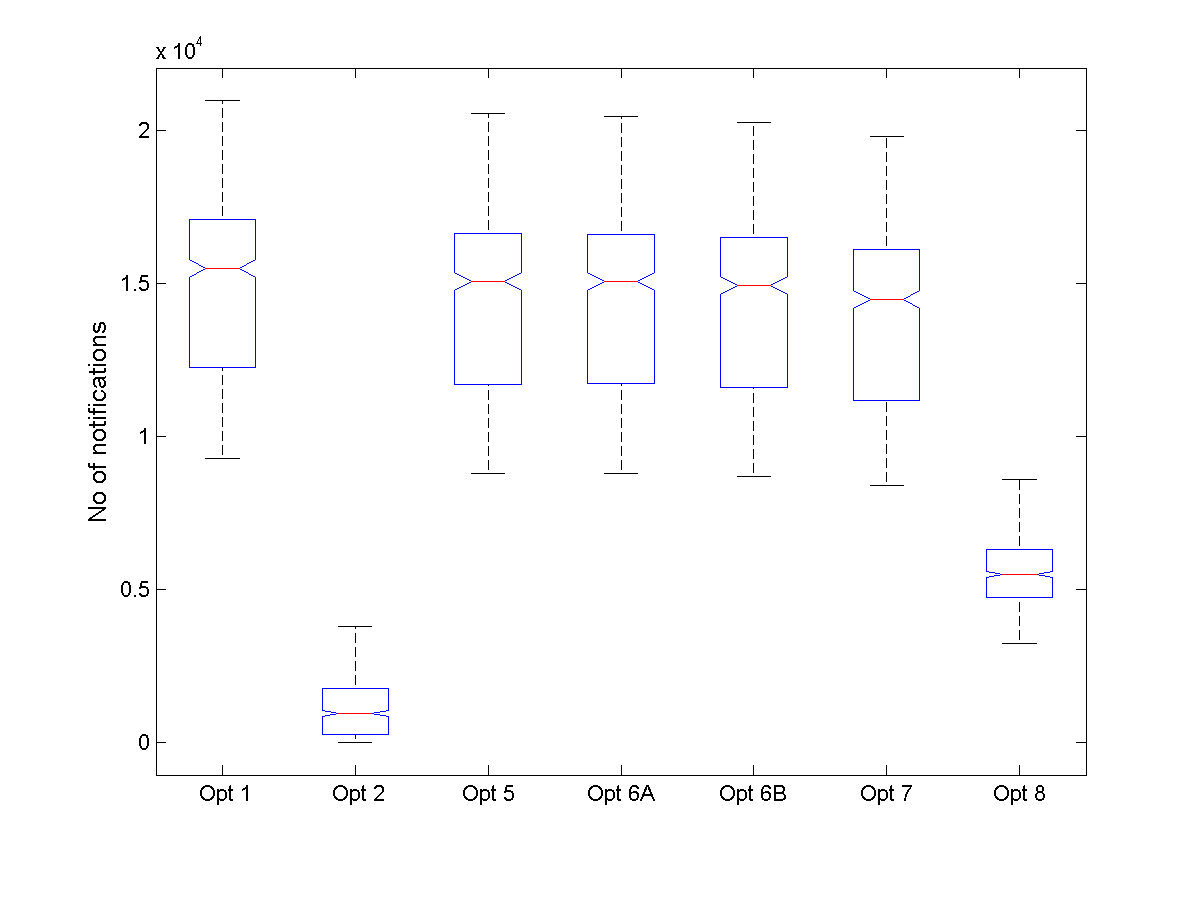


Figure A.3. Box-plots of the model outputs of the cumulative number of pertussis notifications among infants under a year between 2016 and 2030 in England and Wales with the eight different vaccination programme options (1, 2, 5, 6A, 6B, 7 and 8) as shown in Table 2 (red line for median, blue box for 25% and 75% quartiles and black bars for minimum and maximum).

1. **Results from Assortative mixing approach**

For the sensitivity analysis of the mixing matrix, the classical assortative (or proportionate) mixing approach was implemented in the same way as the POLYMOD matrix. At the pre-vaccination equilibrium, the fitted Force of Infections (Figure 3 in main text) could provide two extreme mixing matrices (fully assortative and fully proportionate) for each combined scenario of varied duration of natural immunity and α2/α1 in Table 1 in main text as performed with the POLYMOD contact matrix. A mixing parameter, ε, weighting between these two extreme matrices is determined by how much herd protection is spread among unvaccinated age groups using the post-vaccination data: the mixing matrix is calculated as follows;

Mixing Matrix = ε × Fully Assortative + (1 – ε) × Fully Proportionate.

After running simulations for the post wP vaccination period between 1956 and 2000, 178 scenarios representing the top 5% were selected using the Poisson Deviance of the model outputs to the historical notification data during the corresponding period between 1956 and 2000 as conducted with the POLYMOD mixing approach described in the main paper. Based on the selected wP scenarios, the post aP vaccination model was run further to 2013, and 658 simulated aP scenarios were selected also using the Poisson Deviance of the model outputs to the notification data between 2001 and 2013 and provide the final sets of model parameters to run for the long-term simulation options investigating different vaccination programme scenarios.

The best fitting scenarios suggest very assortative age group mixing as the higher the mixing parameter is more assortative than proportionate in Figure A.4.d.


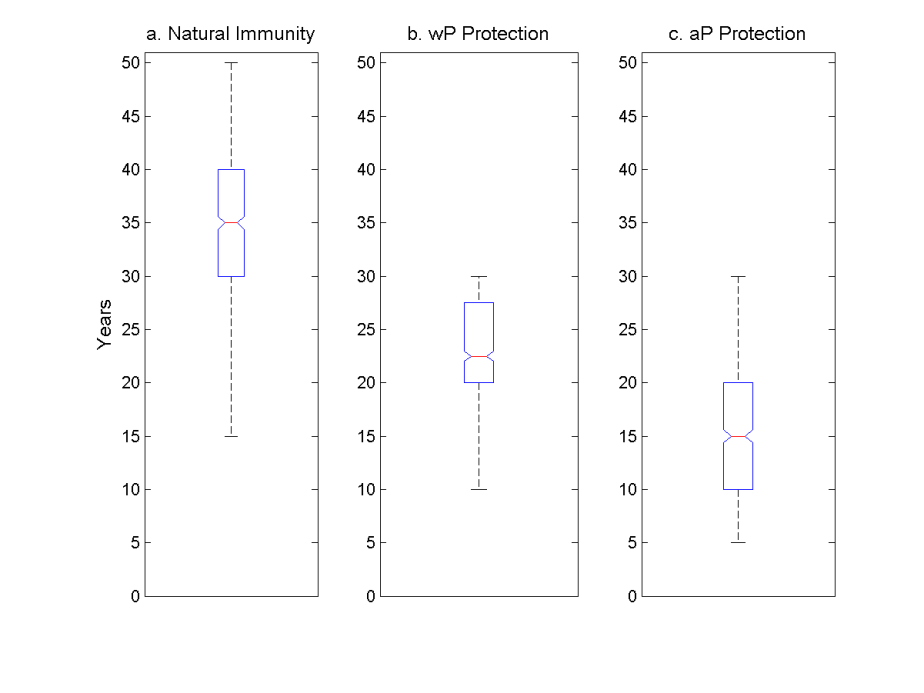

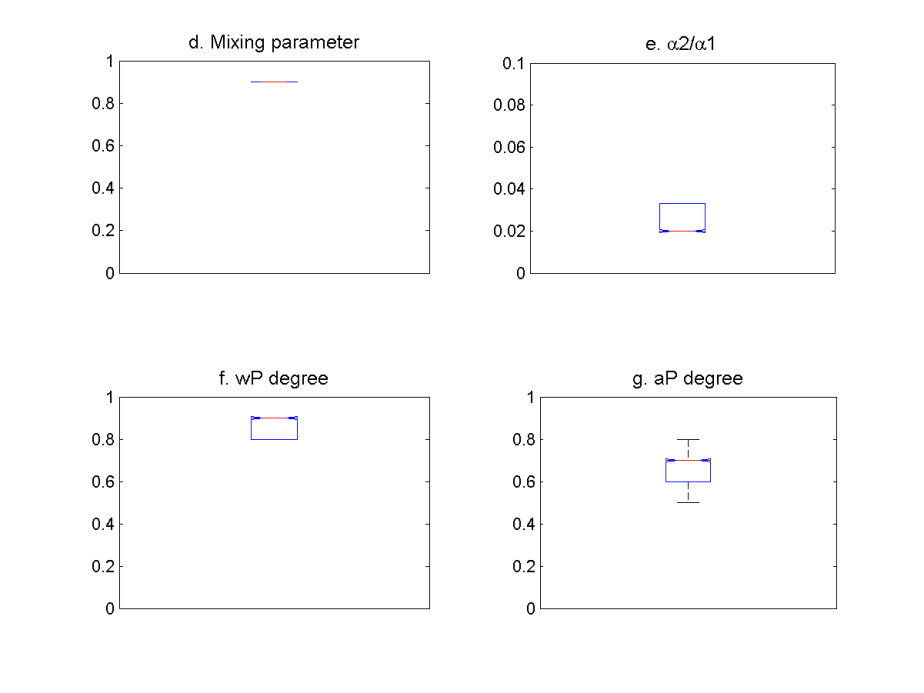


Figure A.4. Box-plots of seven model parameters from the best fitted scenarios when using the assortative matrix instead of the POLYMOD mixing matrix in England and Wales between 1956 and 2013.

Model parameters estimated using the assortative mixing matrix (Figure A.4) were similar to those obtained using the POLYMOD contact mixing approach in Figure 4 in the main text.

The best fitted scenarios with the assortative matrix approach were also simulated to observe the impact of different vaccination options as in Table 2 in the main text. The accumulated notification incidences during the 15 year period between 2016 and 2030 according to different vaccination options (the same options chosen to present in Figure 6 in the main paper) are presented in Figure A.5.


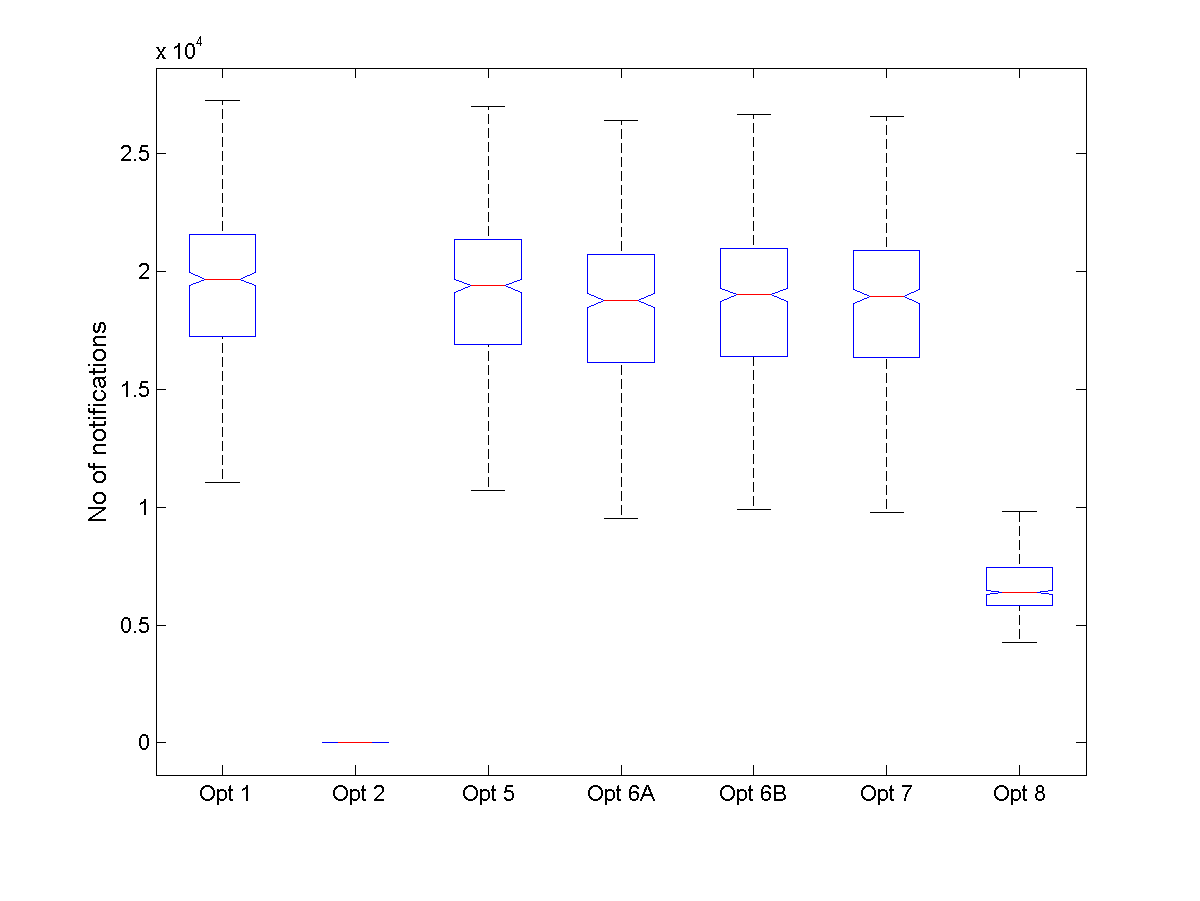


Figure A.5. Box-plots of the model outputs of the cumulative number of pertussis notifications among infants under a year between 2016 and 2030 in England and Wales with the seven different vaccination programme options as shown in Figure 6 in the main paper with the assortative mixing approach and excluding maternal immunisation (red line for median, blue box for 25% and 75% quartiles and black bars for minimum and maximum).

1. **Guideline for installation and operation of the Graphical User Interface (GUI) of the Pertussis model simulation results**
2. Installation
3. You need to verify the MATLAB Compiler Runtime (MCR) is installed and ensure you have installed version 8.2 (R2013b).
4. If the MCR is not installed on your computer, you need to install it.
   1. Go to Mathworks website and download the MATLAB runtime on <http://uk.mathworks.com/products/compiler/mcr/>.
   2. The version you need to download is R2013b and you can choose either 32bit or 64bit depending on your computer.
5. Once you execute the downloaded programme on you machine, you are ready to run the GUI programme.
6. Operation
7. You need to extract files from the file, “Pertussis_GUI.zip”.
8. After extracting the zip file where you wanted to save the files, you need to run “Pertussis_GUI.exe”.
9. After starting the “Pertussis_GUI.exe”, the main programme will be opened (it takes a couple of minutes to open). This GUI enables users to choose various options on three categories: 1. Mixing pattern (POLYMOD or Assortative mixing), 2. Vaccination programme options, and 3. Age groups.
10. By changing the options, corresponding results will be presented accordingly.
11. Graphs presented on the screen are available as “tif” files using the mixing matrix approach, vaccination options, and age groups in the extracted files.
    1. For the POLYMOD outputs, each file is specified as follows:

VacNo = Vaccination option number,

AgeNo = Age Group number, and

File Name = POLYMOD_GUI_VacNo_AgeNo.tif.

- 1. For the WAIFW outputs, each file is specified as follows:

File Name = WAIFW_GUI_VacNo_AgeNo.tif.
