# Supplementary figures and images for: Investigating the pertussis resurgence in England and Wales, and options for future control (part 1 of 2)
Source: BMC Med. 2016 Sep 1;14(1):121. doi: 10.1186/s12916-016-0665-8 (PMC5007864; doi:10.1186/s12916-016-0665-8)

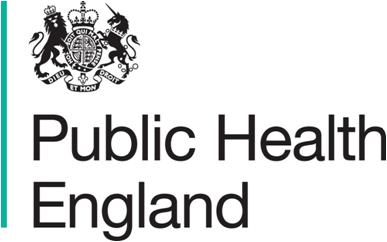

Supplement: Additional file 3: — Graphical User Interface porgramme to present pertussis simulation model results. (ZIP 8235 kb) [file 12916_2016_665_MOESM3_ESM.zip › PHE_Logo.TIF]

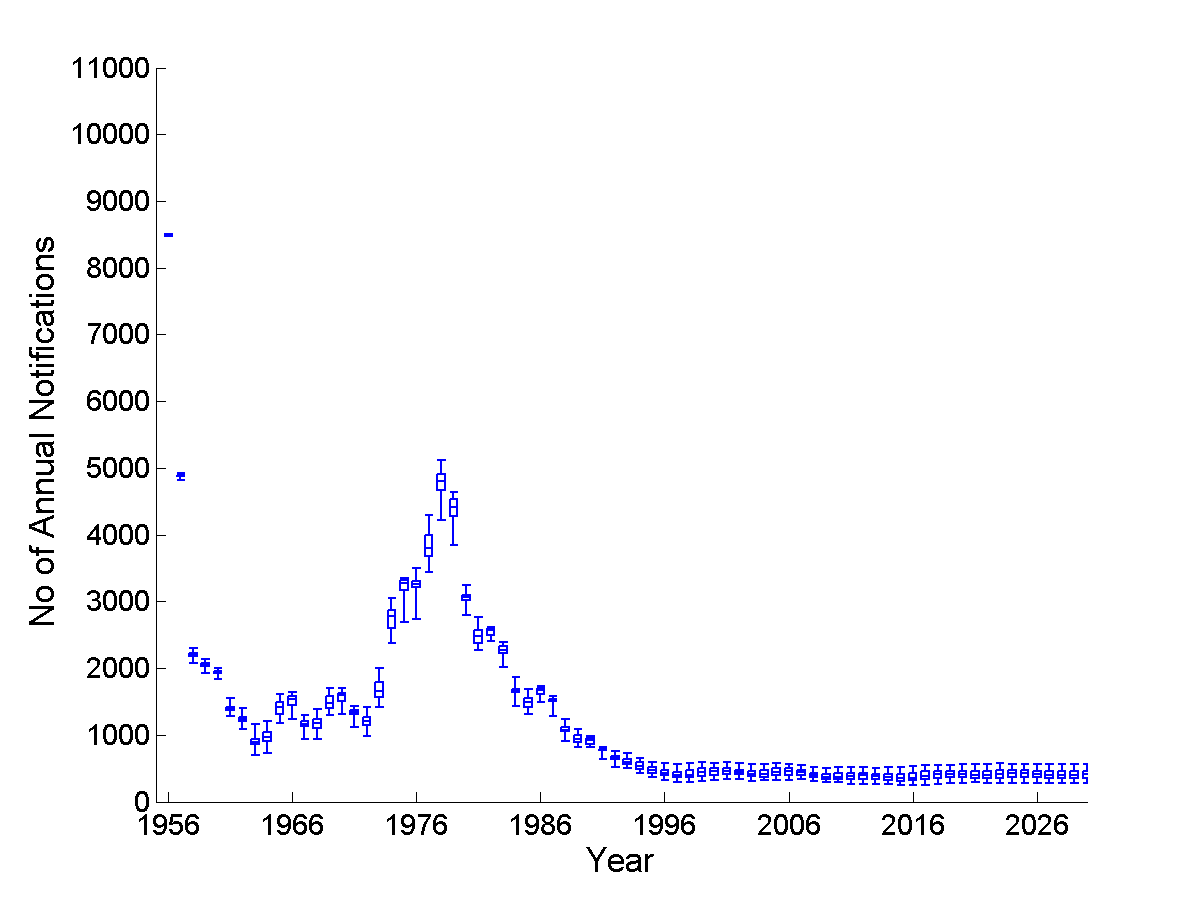

Supplement: Additional file 3: — Graphical User Interface porgramme to present pertussis simulation model results. (ZIP 8235 kb) [file 12916_2016_665_MOESM3_ESM.zip › POLYMOD_GUI_0_1.tif]

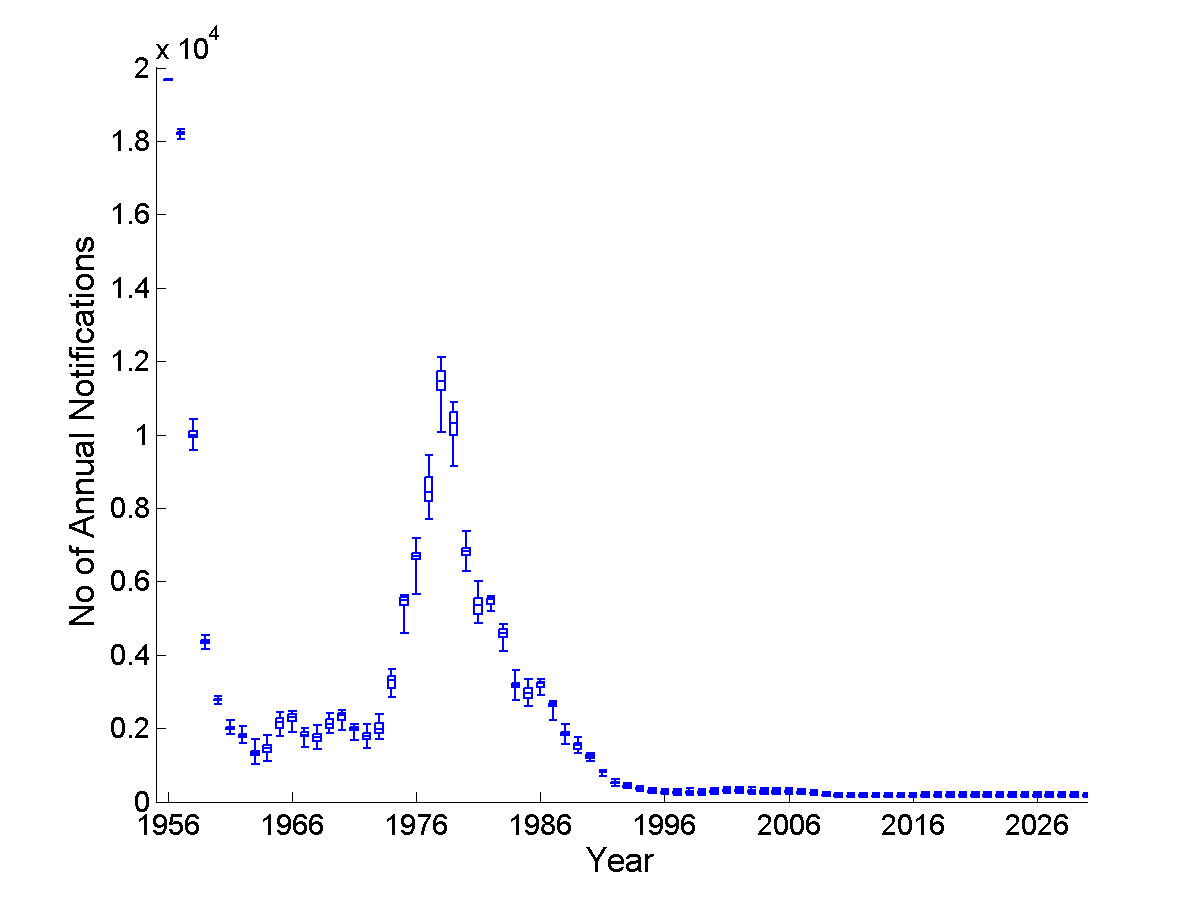

Supplement: Additional file 3: — Graphical User Interface porgramme to present pertussis simulation model results. (ZIP 8235 kb) [file 12916_2016_665_MOESM3_ESM.zip › POLYMOD_GUI_0_2.tif]

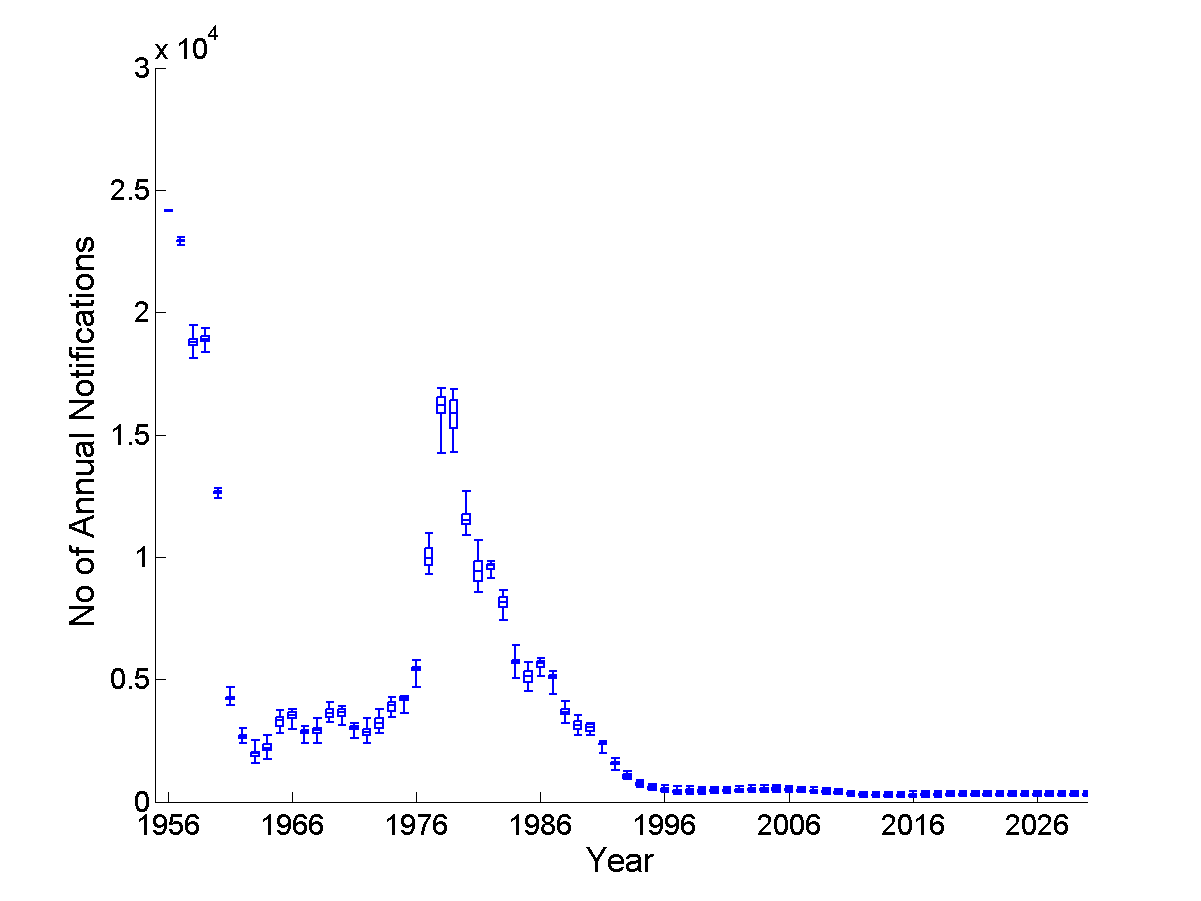

Supplement: Additional file 3: — Graphical User Interface porgramme to present pertussis simulation model results. (ZIP 8235 kb) [file 12916_2016_665_MOESM3_ESM.zip › POLYMOD_GUI_0_3.tif]

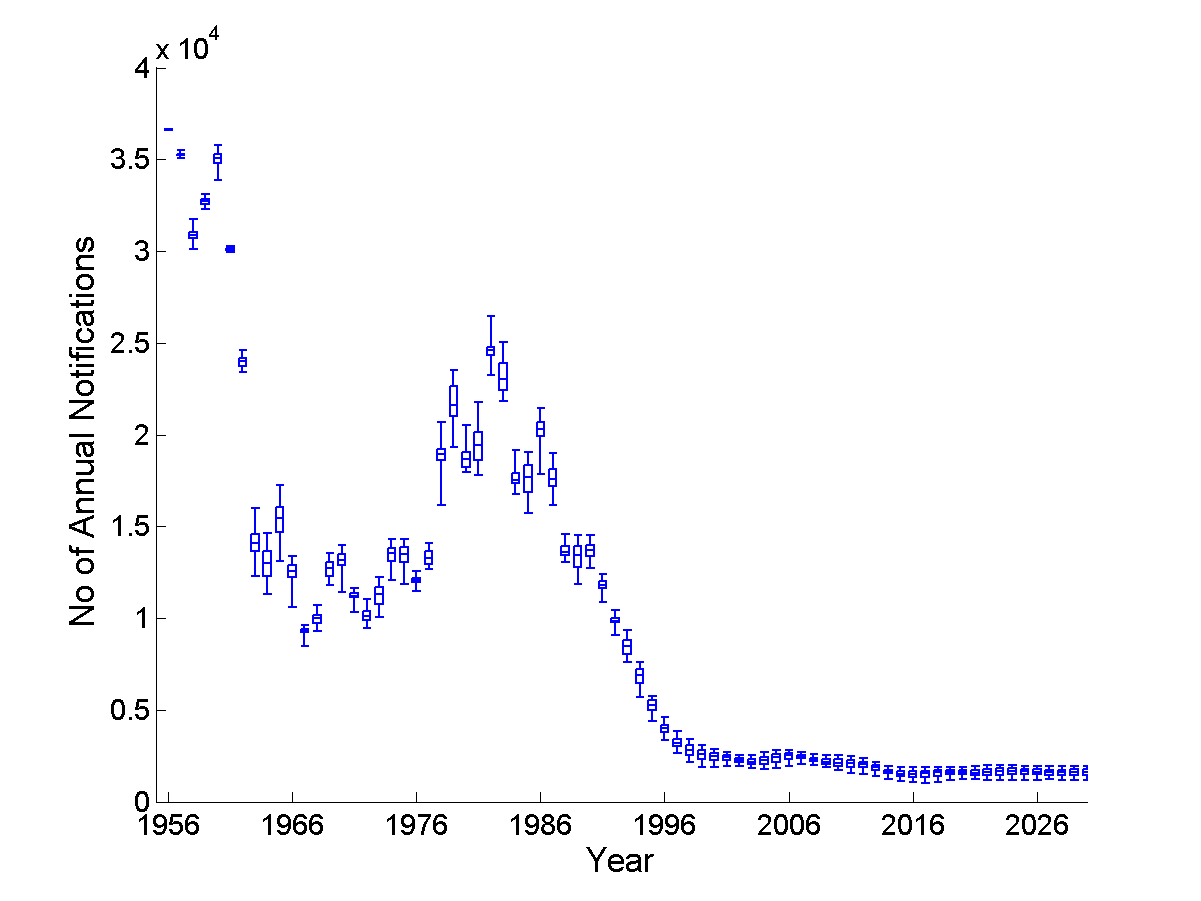

Supplement: Additional file 3: — Graphical User Interface porgramme to present pertussis simulation model results. (ZIP 8235 kb) [file 12916_2016_665_MOESM3_ESM.zip › POLYMOD_GUI_0_4.tif]

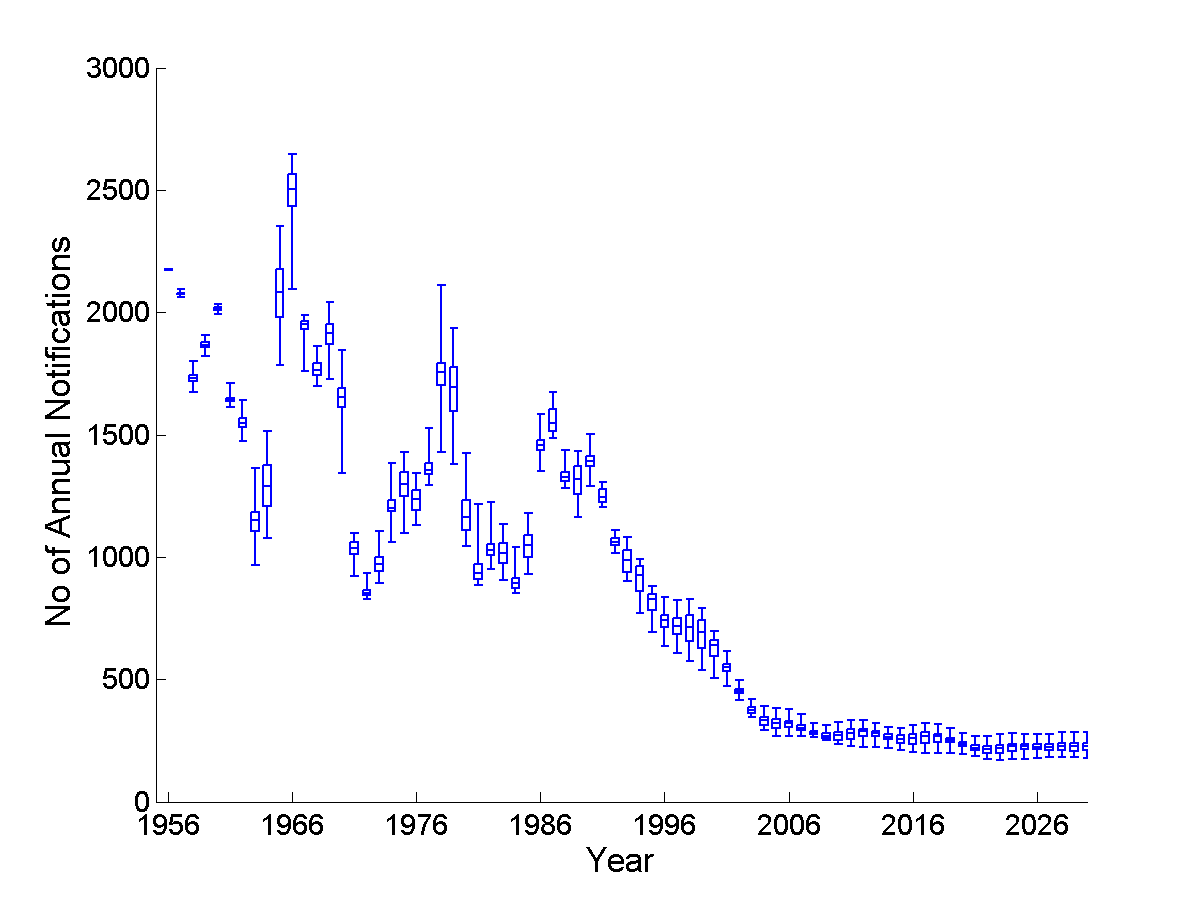

Supplement: Additional file 3: — Graphical User Interface porgramme to present pertussis simulation model results. (ZIP 8235 kb) [file 12916_2016_665_MOESM3_ESM.zip › POLYMOD_GUI_0_5.tif]

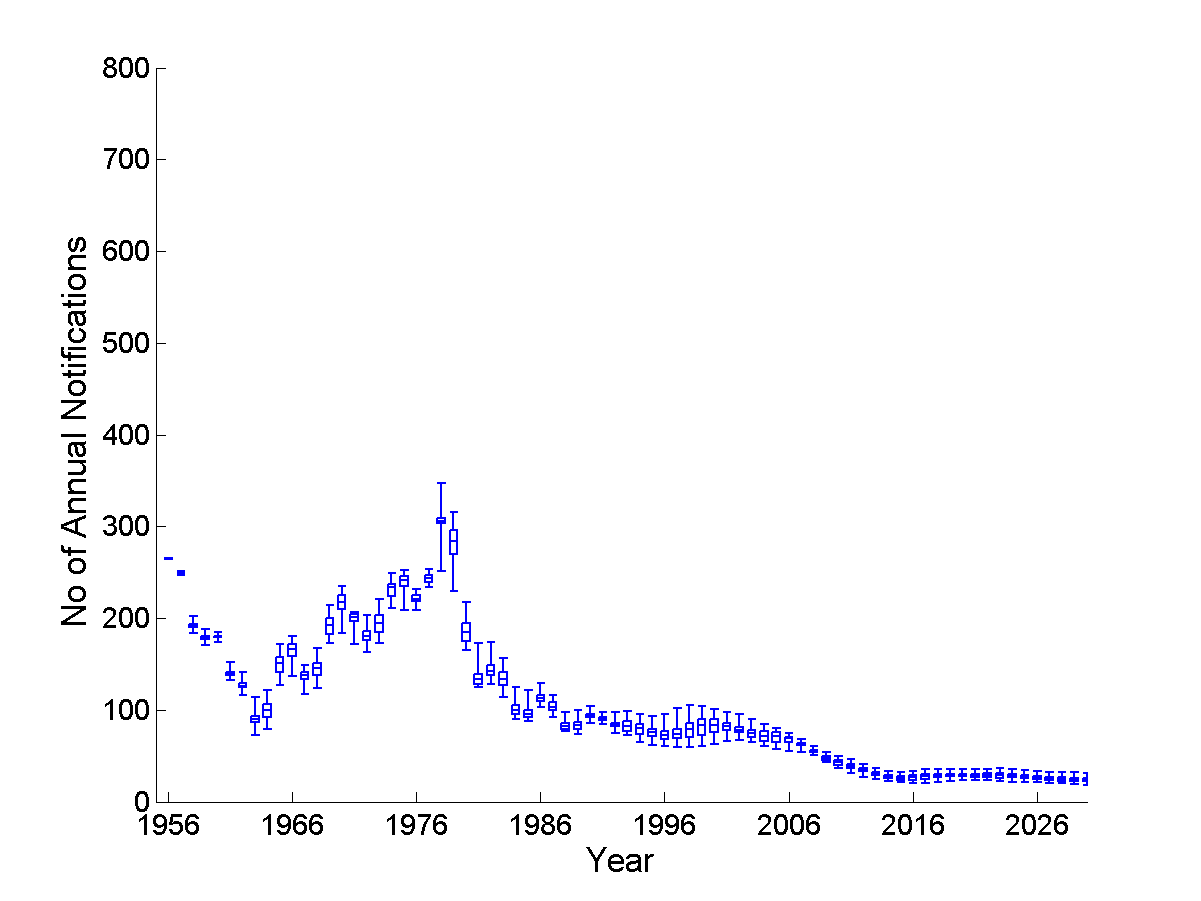

Supplement: Additional file 3: — Graphical User Interface porgramme to present pertussis simulation model results. (ZIP 8235 kb) [file 12916_2016_665_MOESM3_ESM.zip › POLYMOD_GUI_0_6.tif]

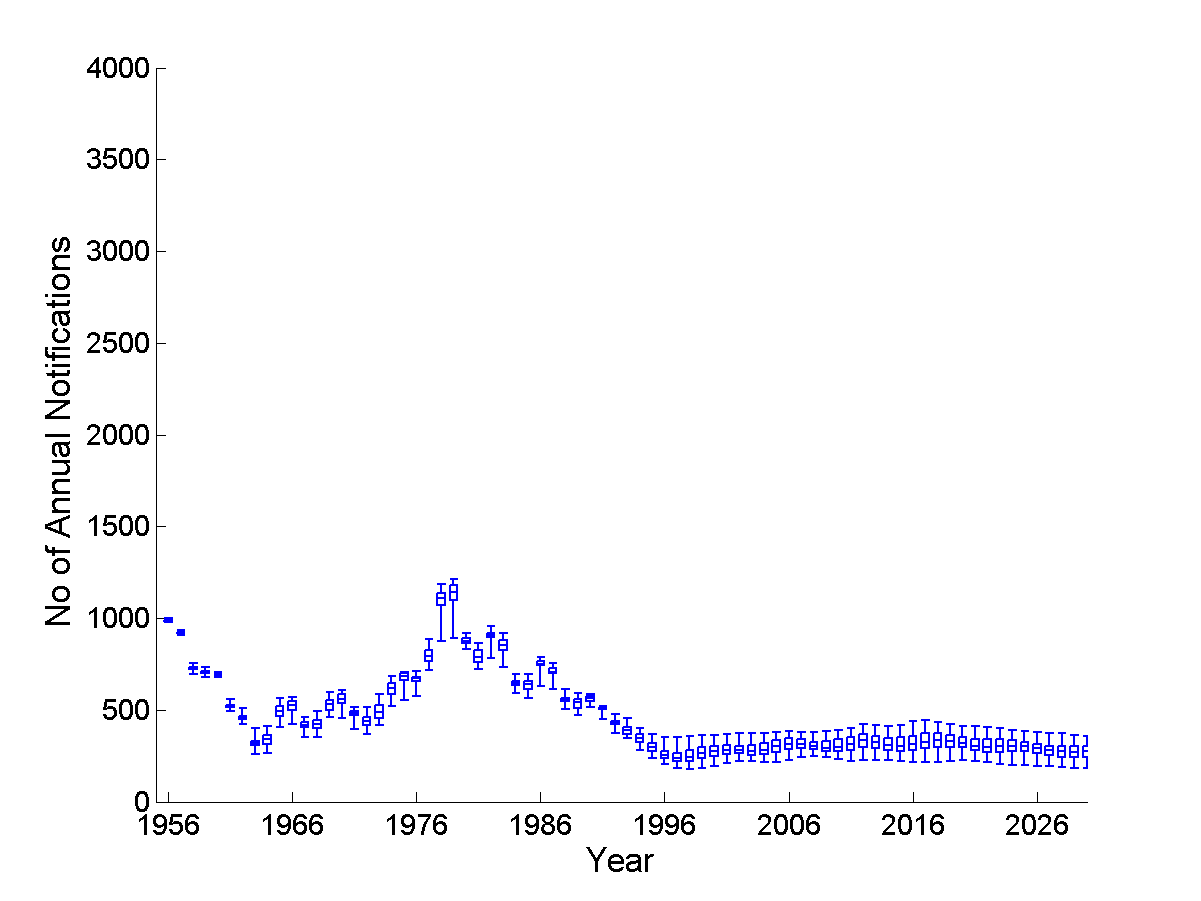

Supplement: Additional file 3: — Graphical User Interface porgramme to present pertussis simulation model results. (ZIP 8235 kb) [file 12916_2016_665_MOESM3_ESM.zip › POLYMOD_GUI_0_7.tif]

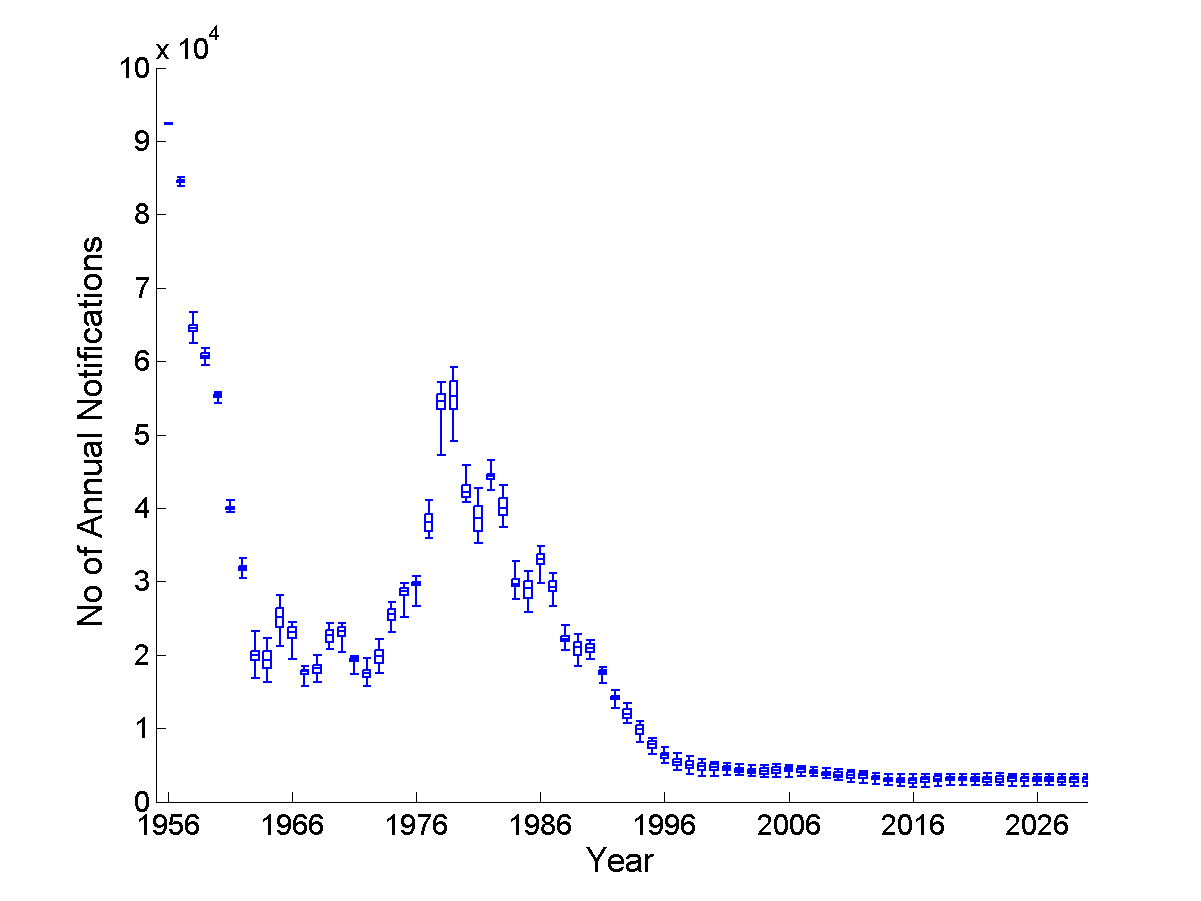

Supplement: Additional file 3: — Graphical User Interface porgramme to present pertussis simulation model results. (ZIP 8235 kb) [file 12916_2016_665_MOESM3_ESM.zip › POLYMOD_GUI_0_8.tif]

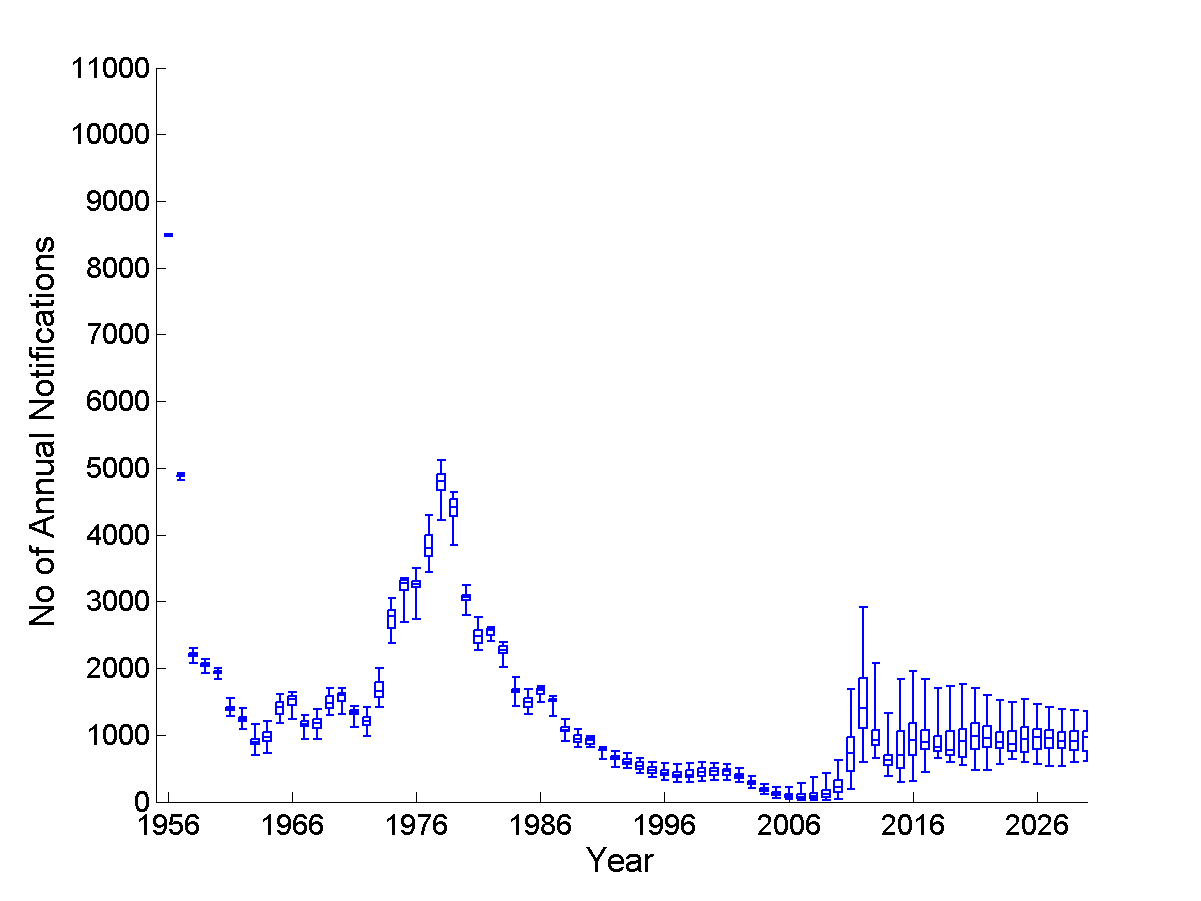

Supplement: Additional file 3: — Graphical User Interface porgramme to present pertussis simulation model results. (ZIP 8235 kb) [file 12916_2016_665_MOESM3_ESM.zip › POLYMOD_GUI_1_1.tif]

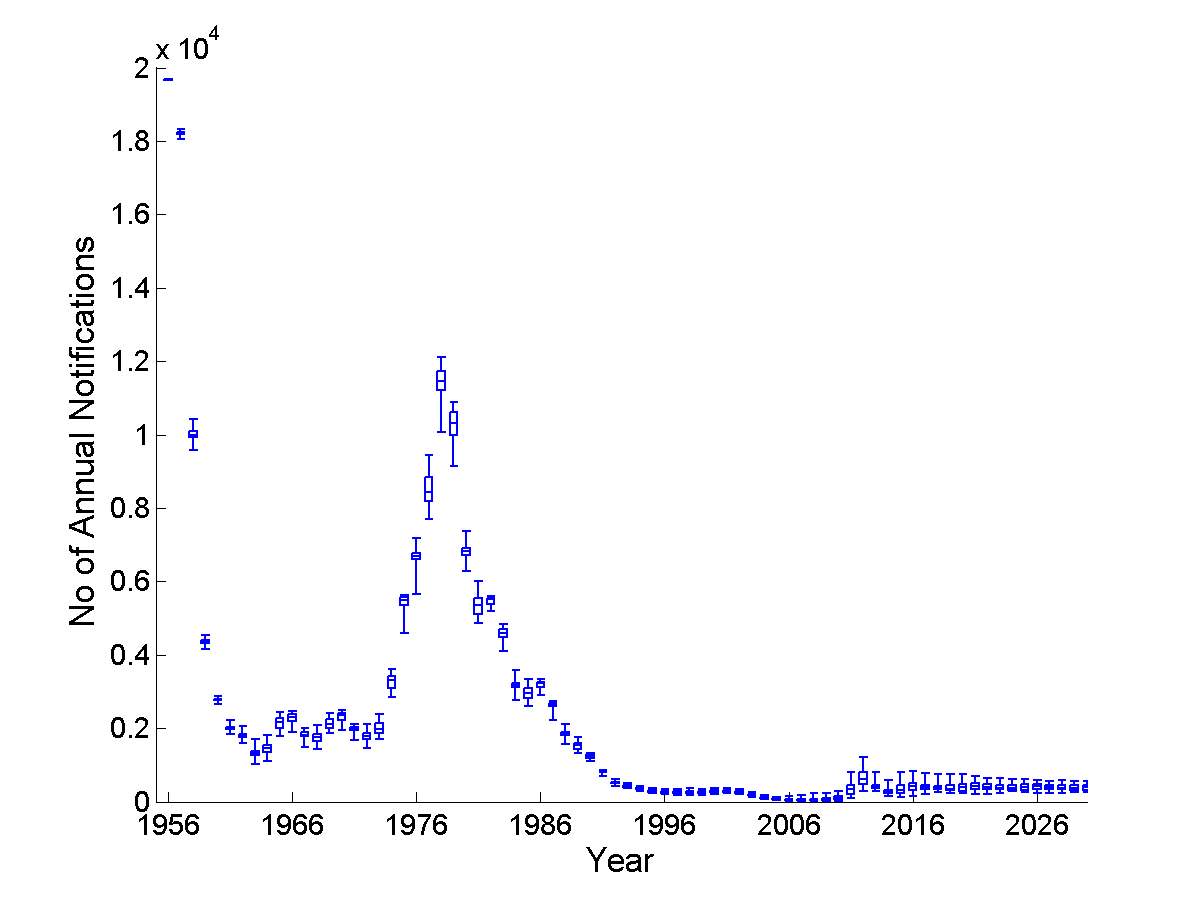

Supplement: Additional file 3: — Graphical User Interface porgramme to present pertussis simulation model results. (ZIP 8235 kb) [file 12916_2016_665_MOESM3_ESM.zip › POLYMOD_GUI_1_2.tif]

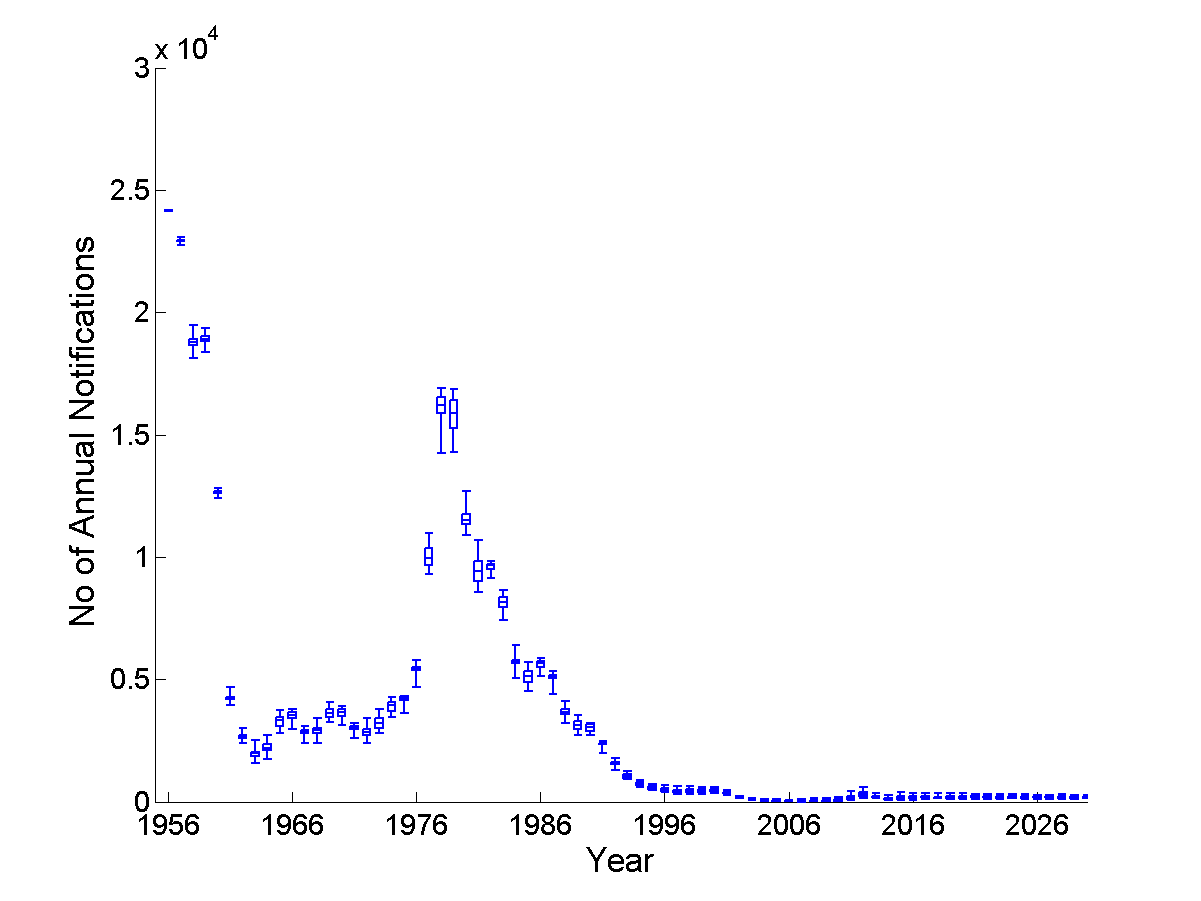

Supplement: Additional file 3: — Graphical User Interface porgramme to present pertussis simulation model results. (ZIP 8235 kb) [file 12916_2016_665_MOESM3_ESM.zip › POLYMOD_GUI_1_3.tif]

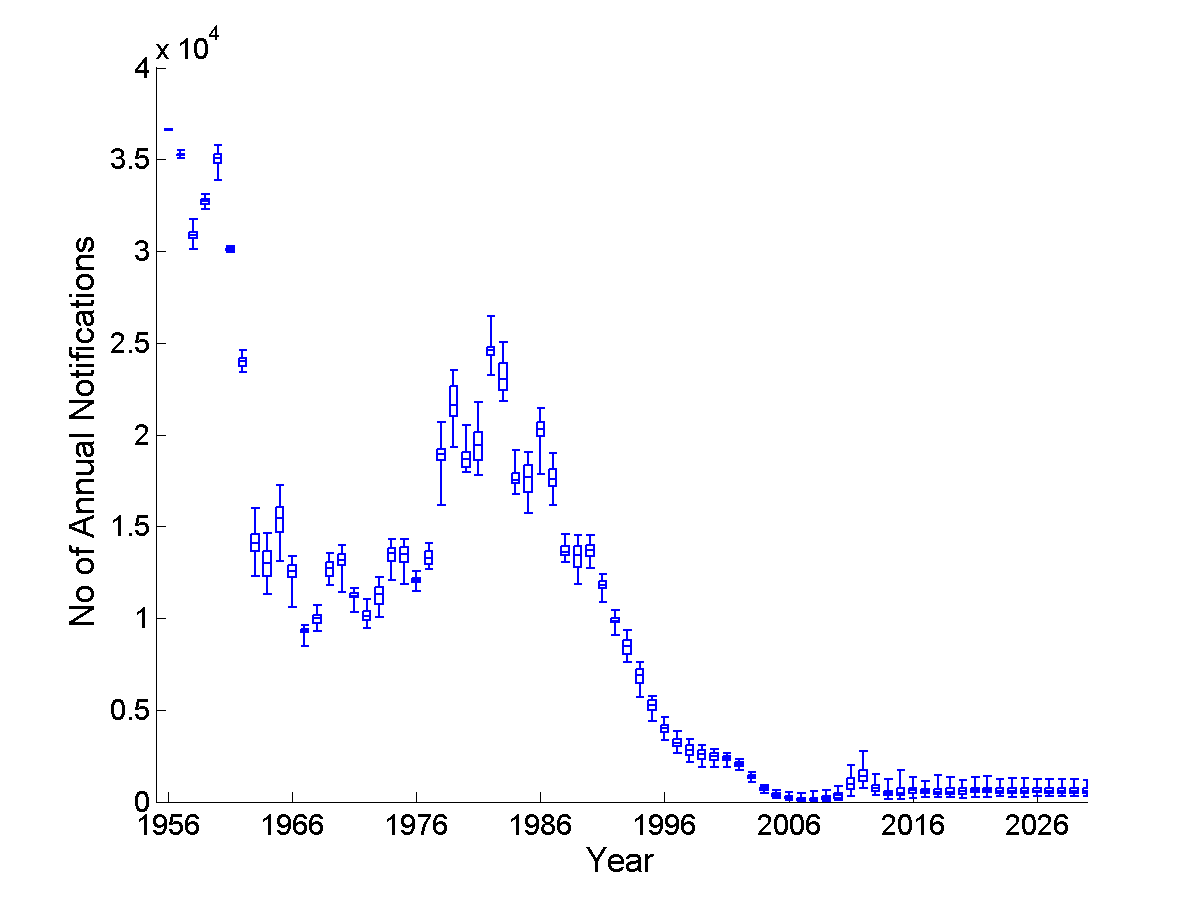

Supplement: Additional file 3: — Graphical User Interface porgramme to present pertussis simulation model results. (ZIP 8235 kb) [file 12916_2016_665_MOESM3_ESM.zip › POLYMOD_GUI_1_4.tif]

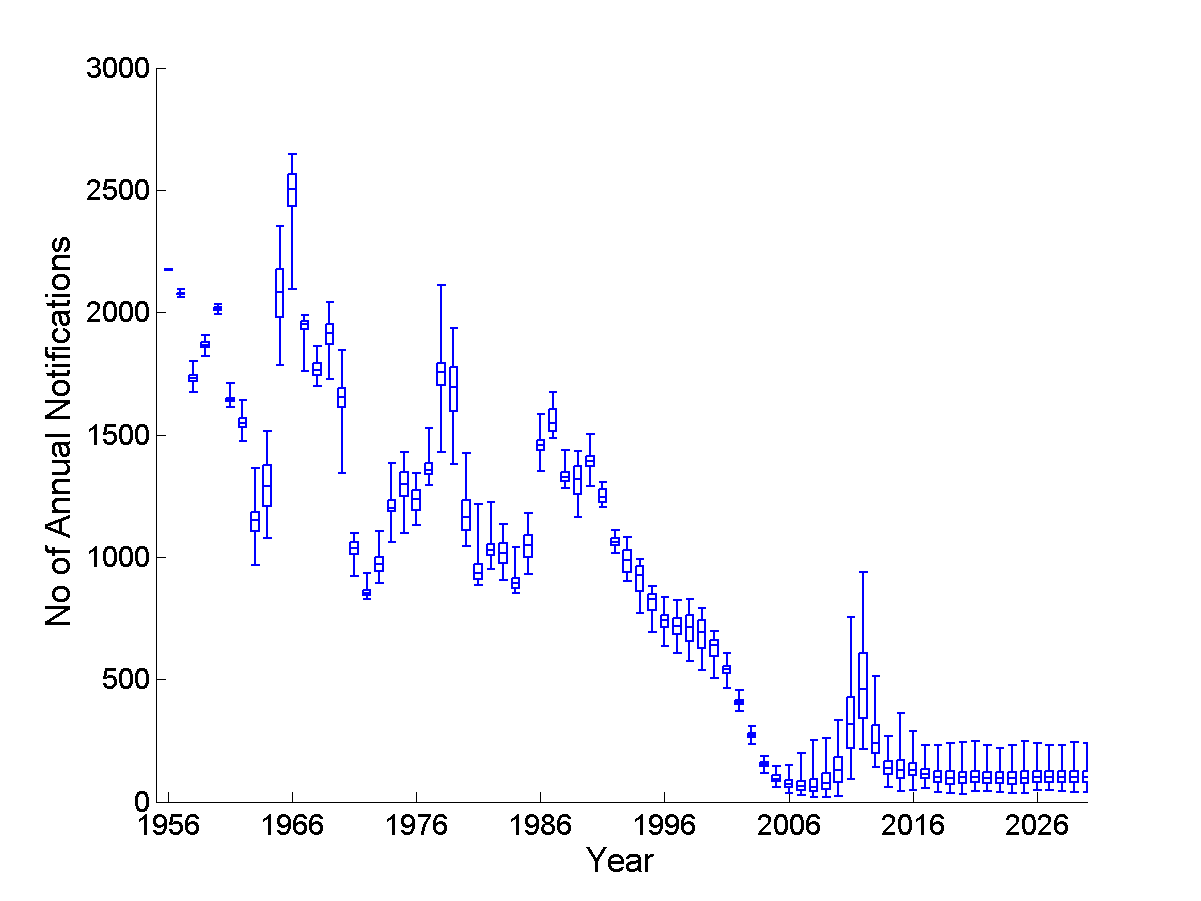

Supplement: Additional file 3: — Graphical User Interface porgramme to present pertussis simulation model results. (ZIP 8235 kb) [file 12916_2016_665_MOESM3_ESM.zip › POLYMOD_GUI_1_5.tif]

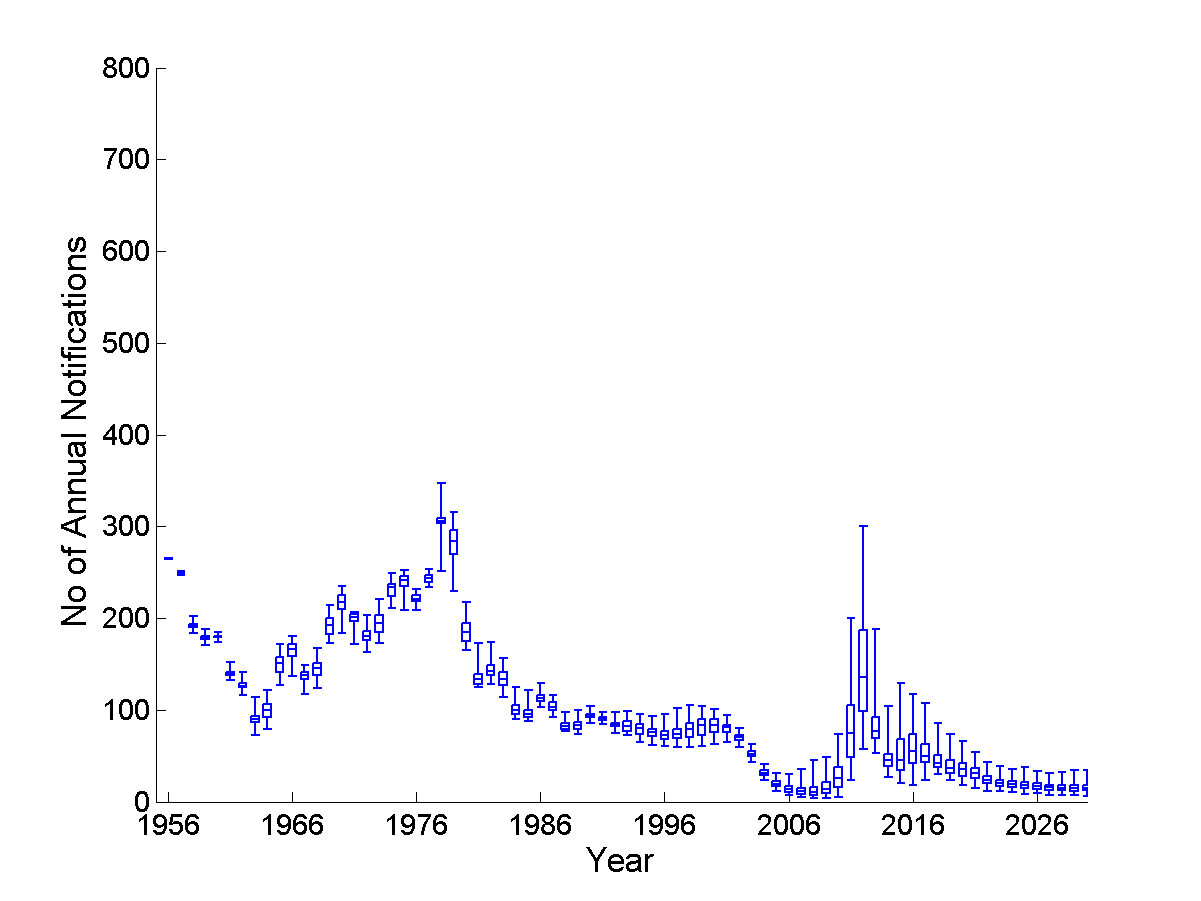

Supplement: Additional file 3: — Graphical User Interface porgramme to present pertussis simulation model results. (ZIP 8235 kb) [file 12916_2016_665_MOESM3_ESM.zip › POLYMOD_GUI_1_6.tif]

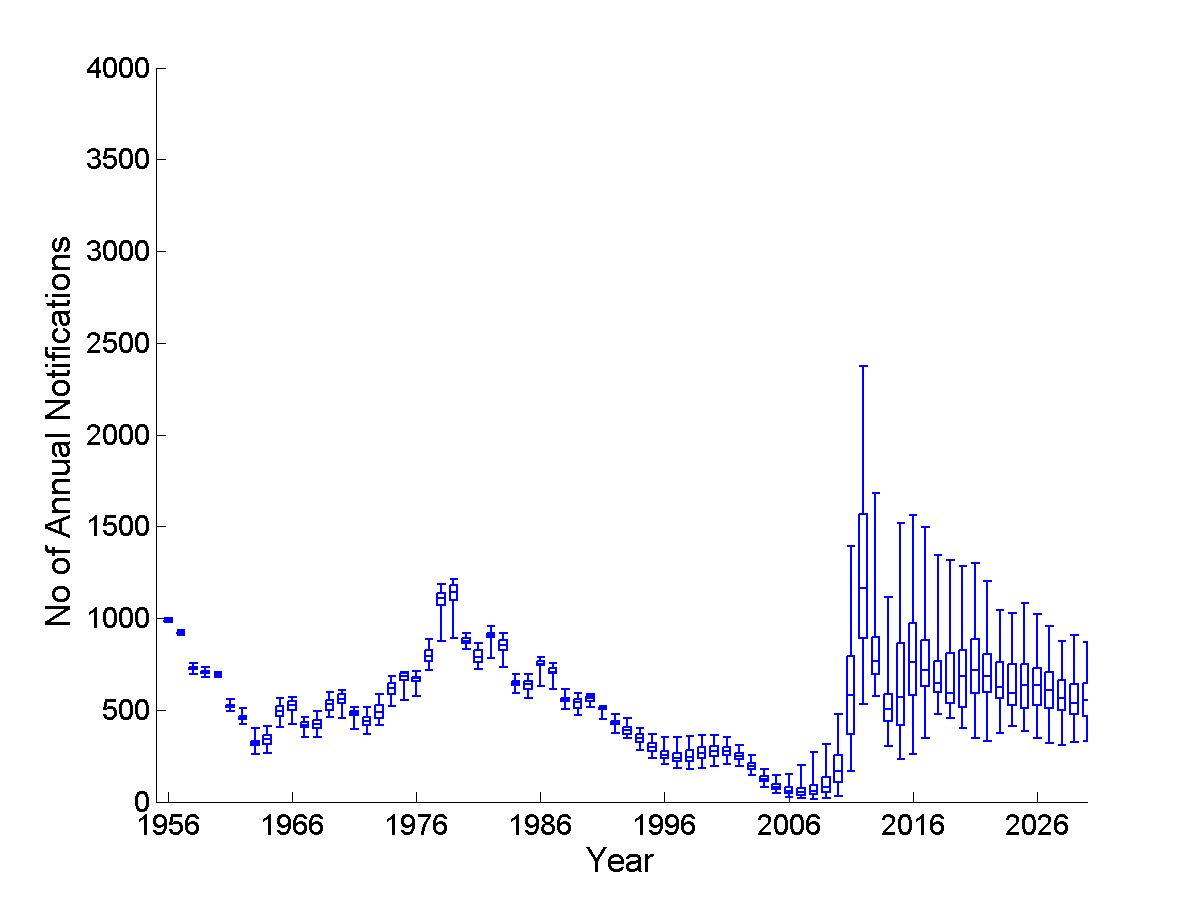

Supplement: Additional file 3: — Graphical User Interface porgramme to present pertussis simulation model results. (ZIP 8235 kb) [file 12916_2016_665_MOESM3_ESM.zip › POLYMOD_GUI_1_7.tif]

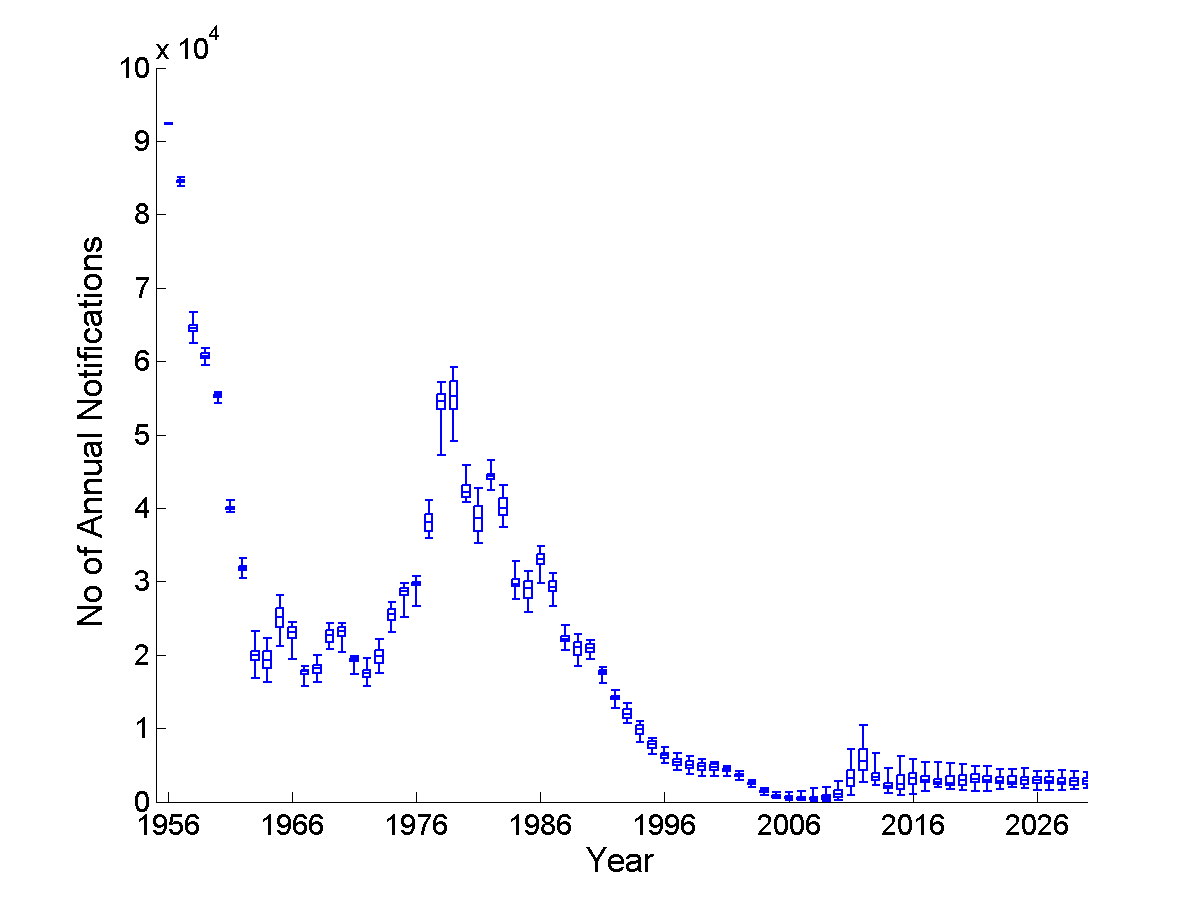

Supplement: Additional file 3: — Graphical User Interface porgramme to present pertussis simulation model results. (ZIP 8235 kb) [file 12916_2016_665_MOESM3_ESM.zip › POLYMOD_GUI_1_8.tif]

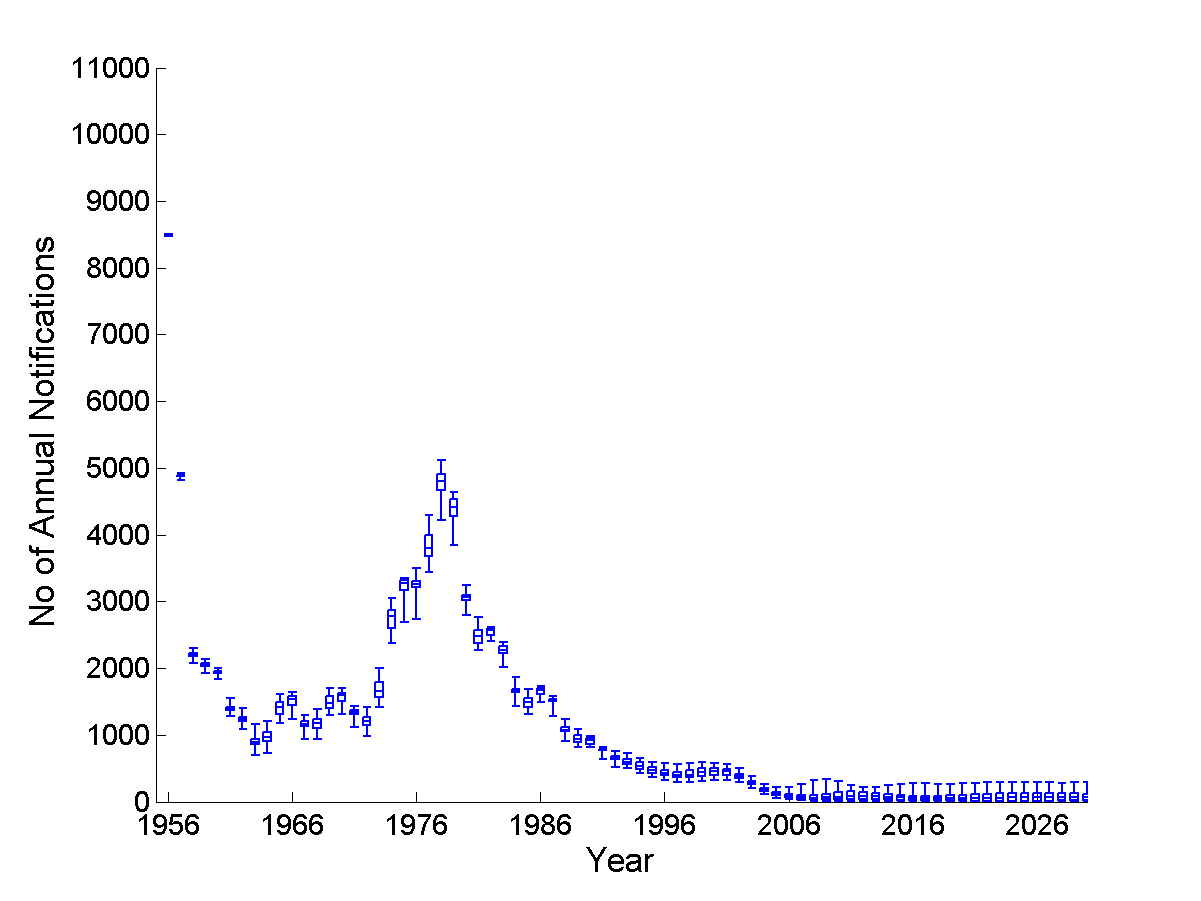

Supplement: Additional file 3: — Graphical User Interface porgramme to present pertussis simulation model results. (ZIP 8235 kb) [file 12916_2016_665_MOESM3_ESM.zip › POLYMOD_GUI_2_1.tif]

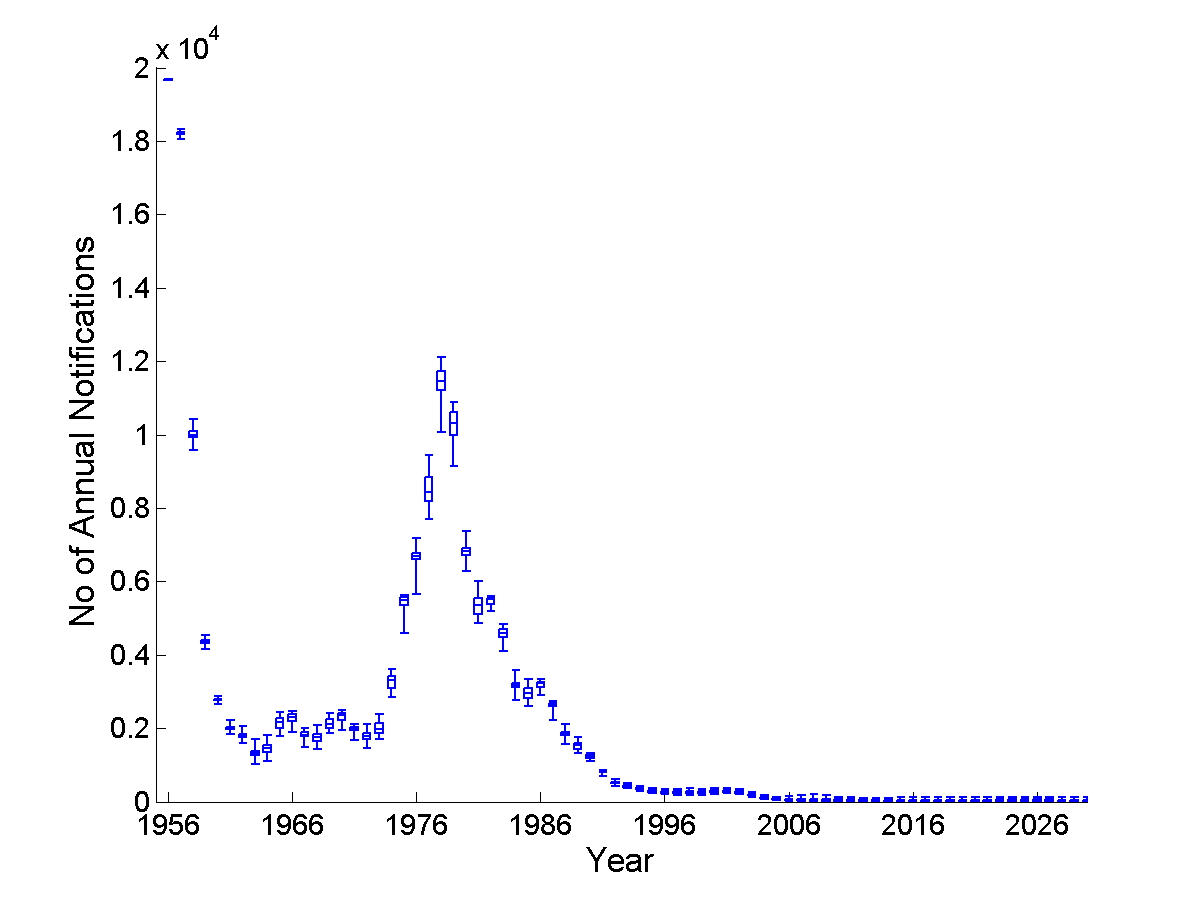

Supplement: Additional file 3: — Graphical User Interface porgramme to present pertussis simulation model results. (ZIP 8235 kb) [file 12916_2016_665_MOESM3_ESM.zip › POLYMOD_GUI_2_2.tif]

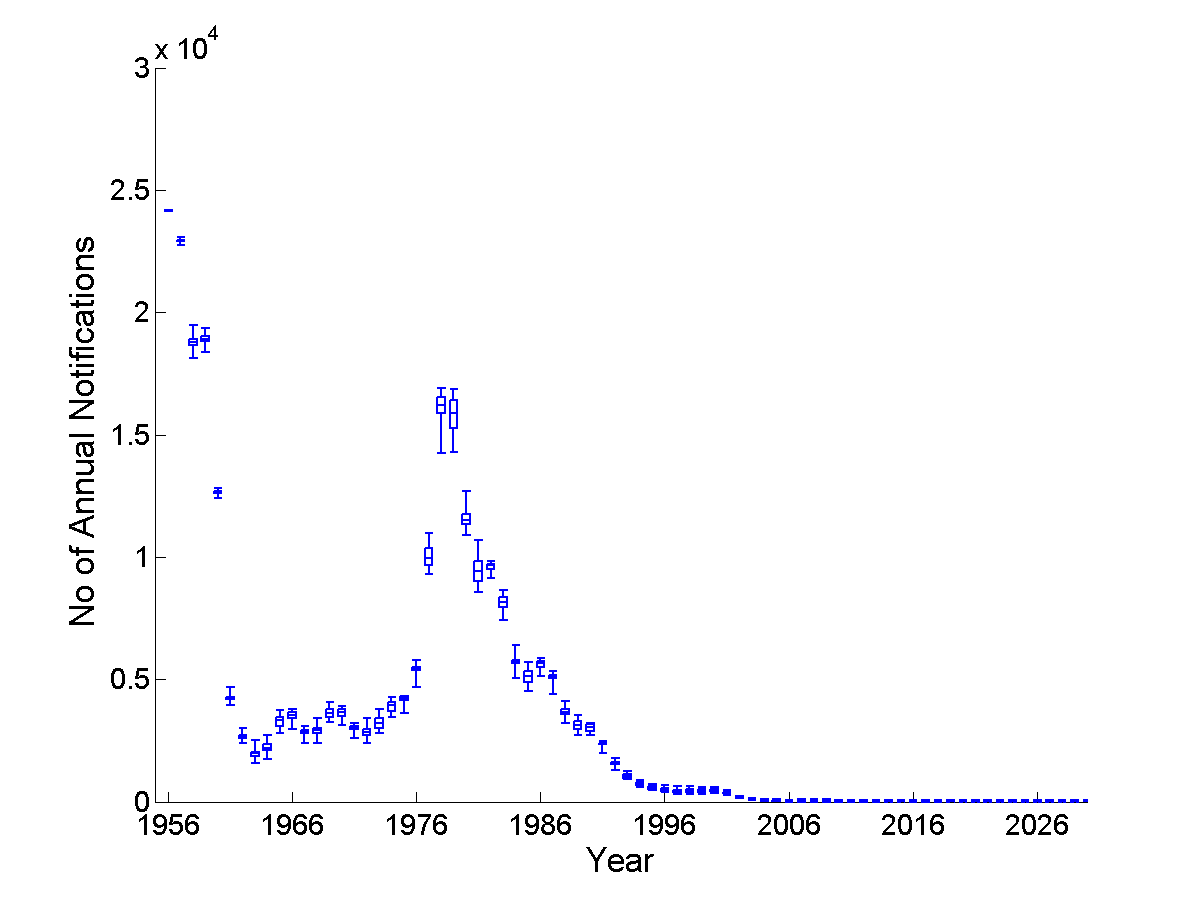

Supplement: Additional file 3: — Graphical User Interface porgramme to present pertussis simulation model results. (ZIP 8235 kb) [file 12916_2016_665_MOESM3_ESM.zip › POLYMOD_GUI_2_3.tif]

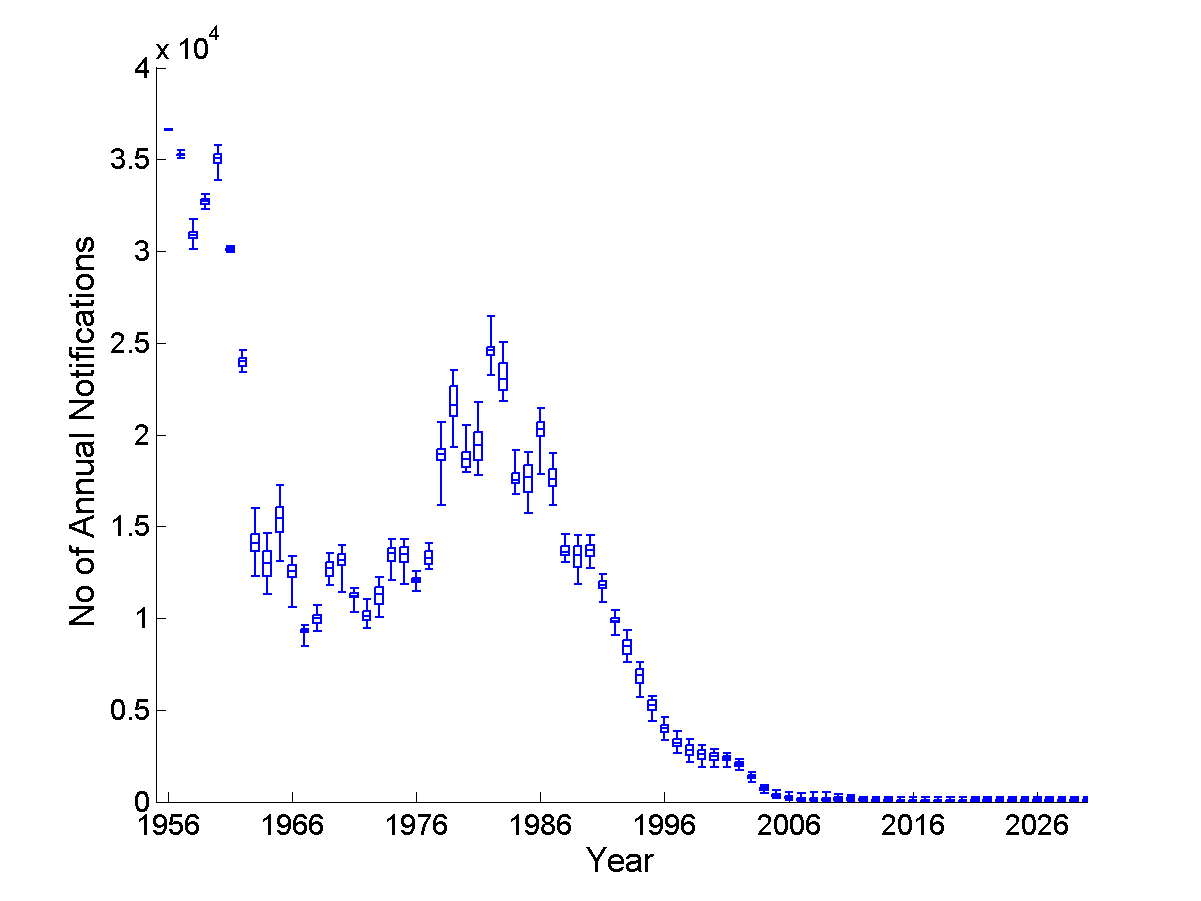

Supplement: Additional file 3: — Graphical User Interface porgramme to present pertussis simulation model results. (ZIP 8235 kb) [file 12916_2016_665_MOESM3_ESM.zip › POLYMOD_GUI_2_4.tif]

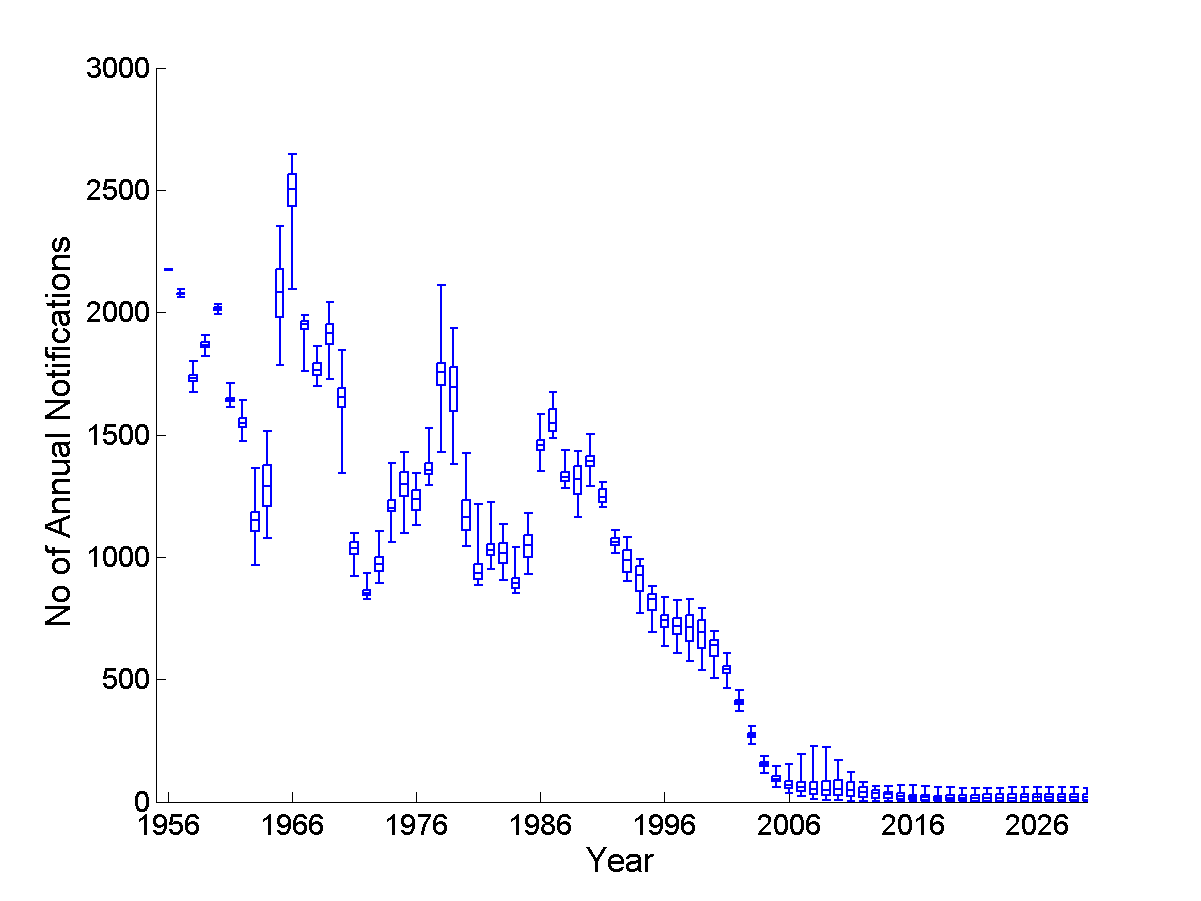

Supplement: Additional file 3: — Graphical User Interface porgramme to present pertussis simulation model results. (ZIP 8235 kb) [file 12916_2016_665_MOESM3_ESM.zip › POLYMOD_GUI_2_5.tif]

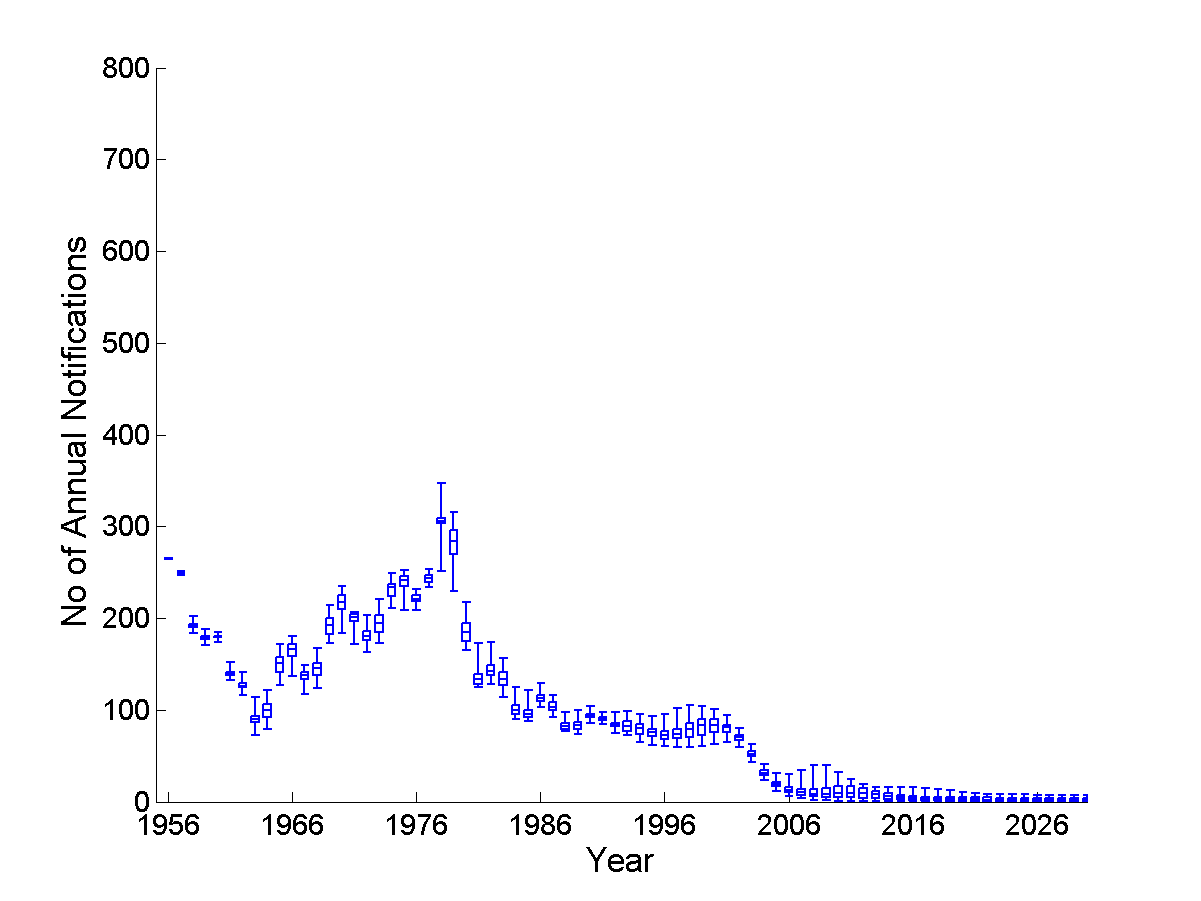

Supplement: Additional file 3: — Graphical User Interface porgramme to present pertussis simulation model results. (ZIP 8235 kb) [file 12916_2016_665_MOESM3_ESM.zip › POLYMOD_GUI_2_6.tif]

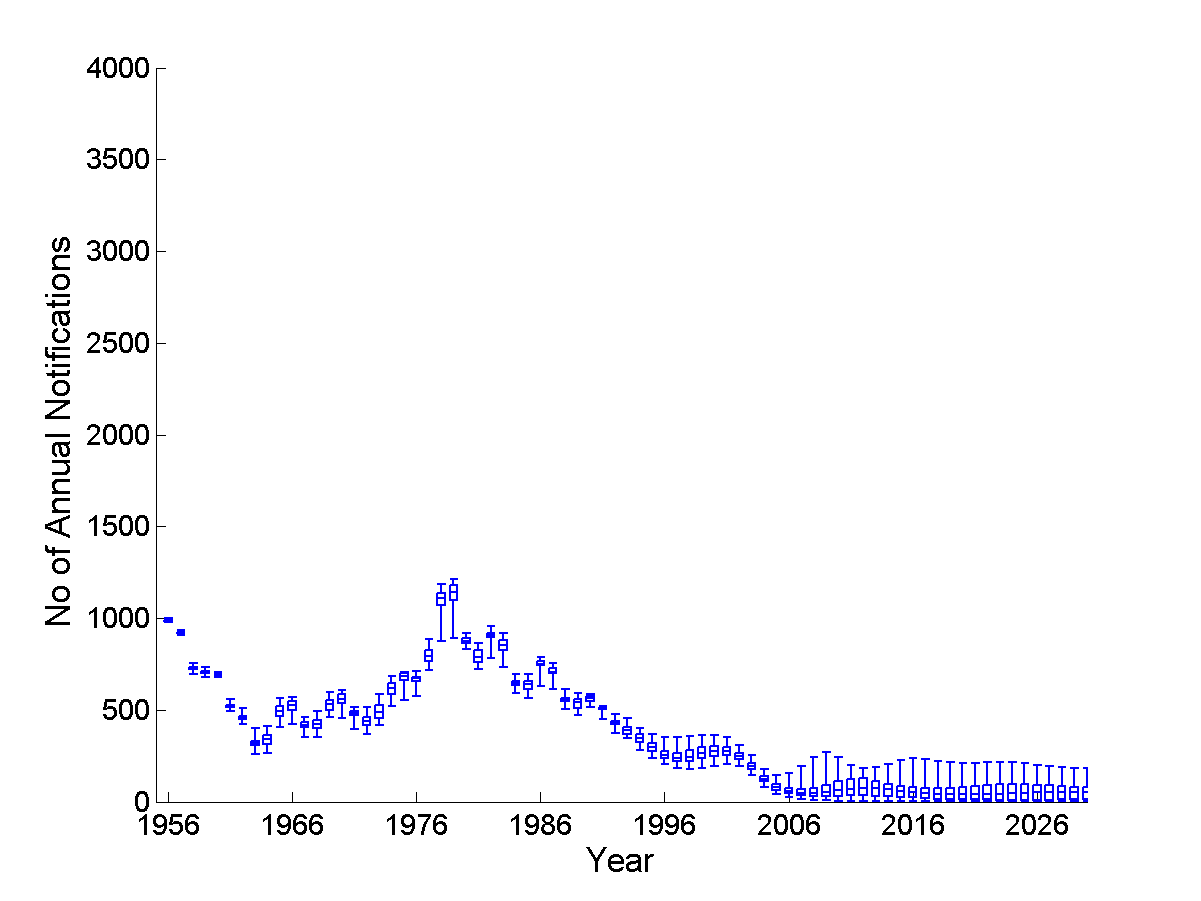

Supplement: Additional file 3: — Graphical User Interface porgramme to present pertussis simulation model results. (ZIP 8235 kb) [file 12916_2016_665_MOESM3_ESM.zip › POLYMOD_GUI_2_7.tif]

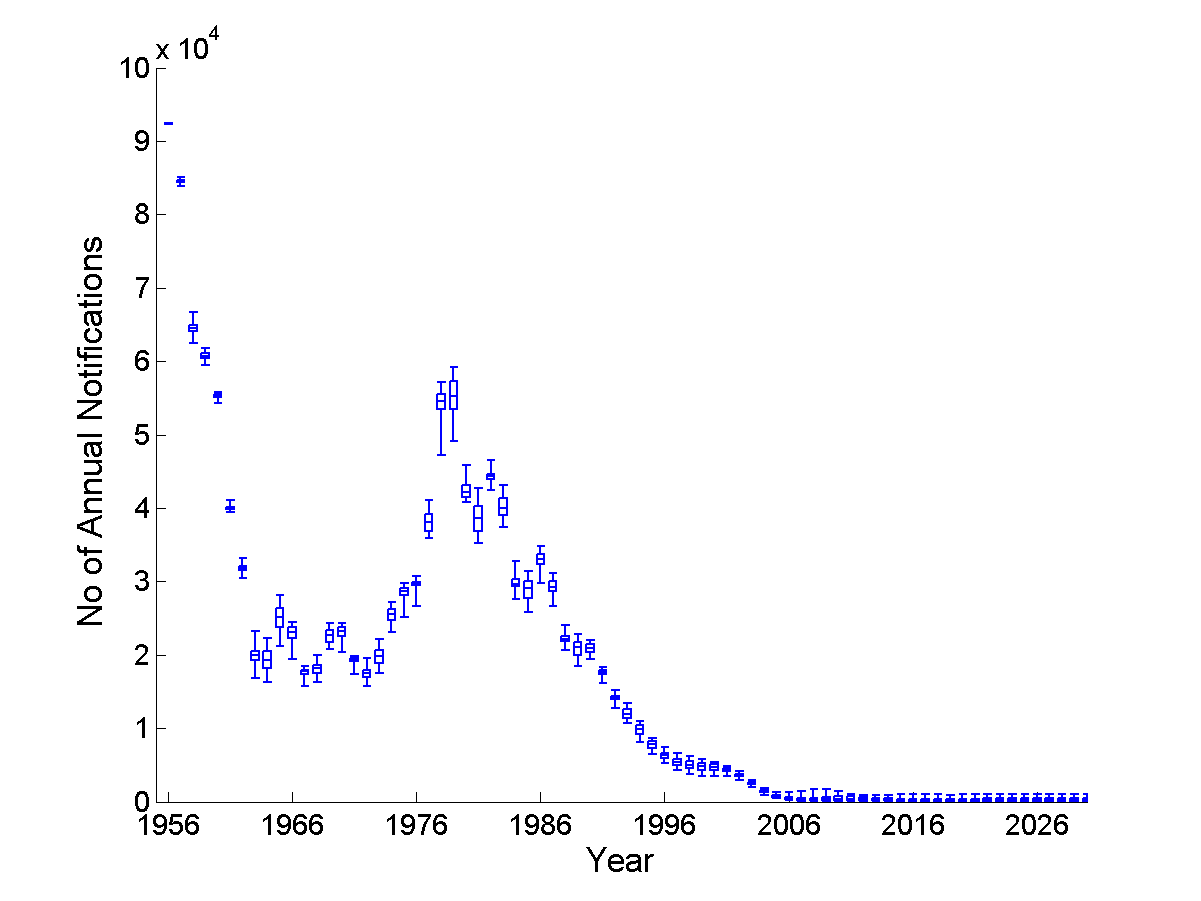

Supplement: Additional file 3: — Graphical User Interface porgramme to present pertussis simulation model results. (ZIP 8235 kb) [file 12916_2016_665_MOESM3_ESM.zip › POLYMOD_GUI_2_8.tif]

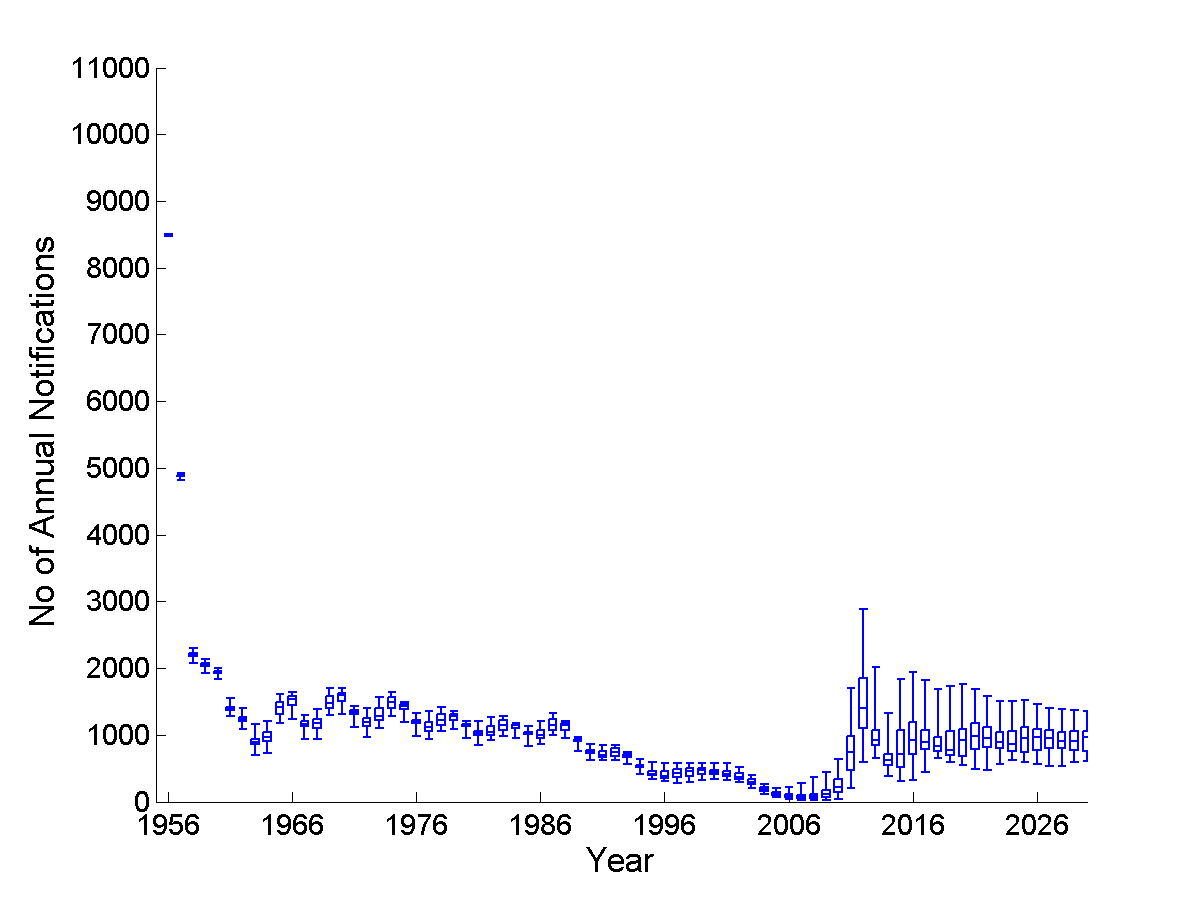

Supplement: Additional file 3: — Graphical User Interface porgramme to present pertussis simulation model results. (ZIP 8235 kb) [file 12916_2016_665_MOESM3_ESM.zip › POLYMOD_GUI_3_1.tif]

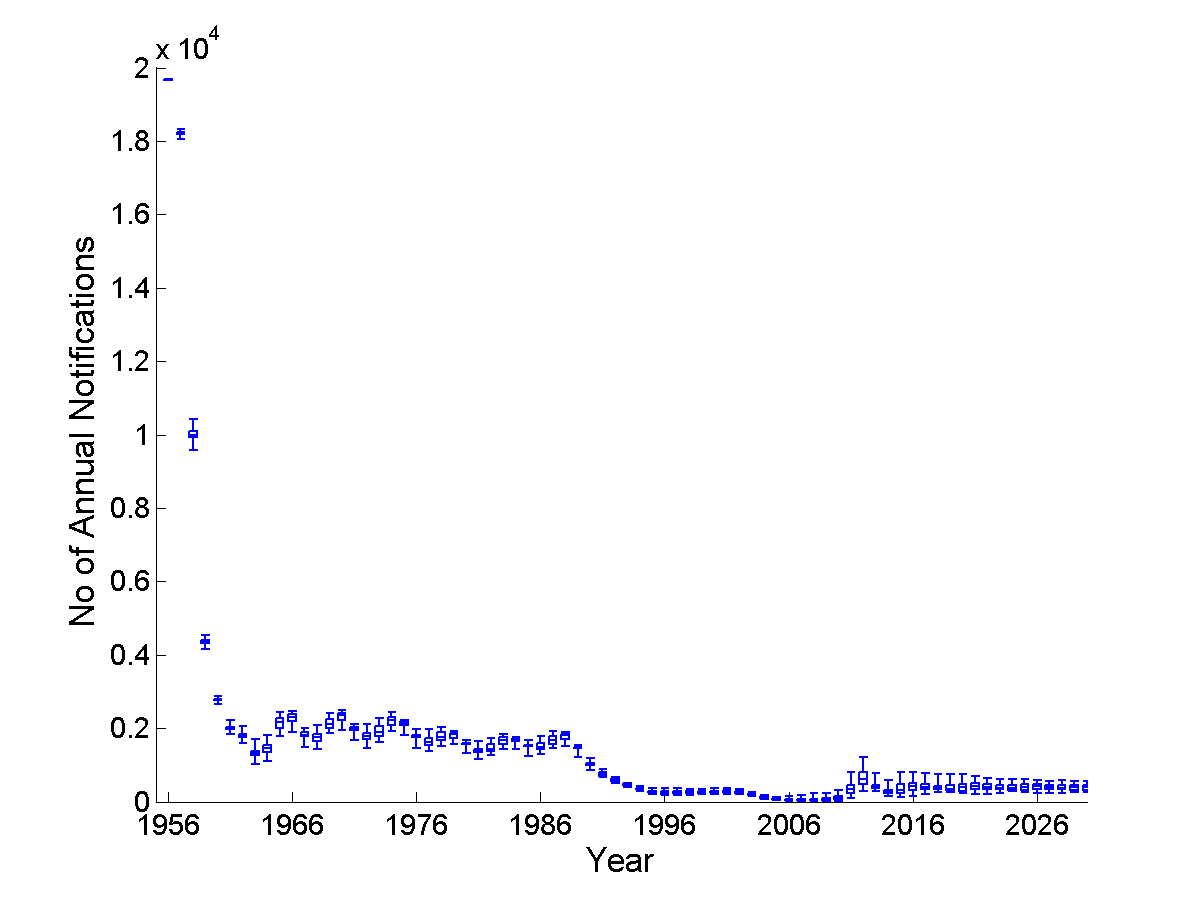

Supplement: Additional file 3: — Graphical User Interface porgramme to present pertussis simulation model results. (ZIP 8235 kb) [file 12916_2016_665_MOESM3_ESM.zip › POLYMOD_GUI_3_2.tif]

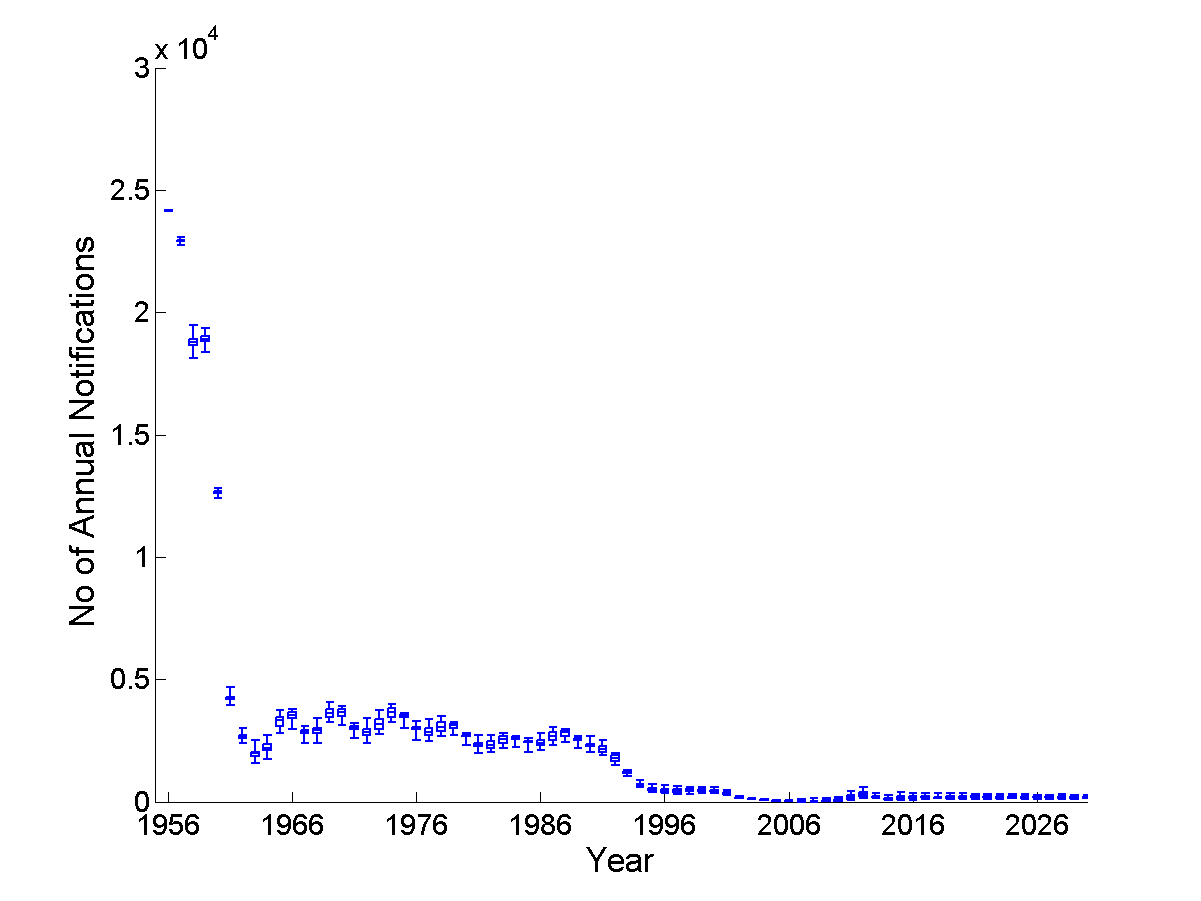

Supplement: Additional file 3: — Graphical User Interface porgramme to present pertussis simulation model results. (ZIP 8235 kb) [file 12916_2016_665_MOESM3_ESM.zip › POLYMOD_GUI_3_3.tif]

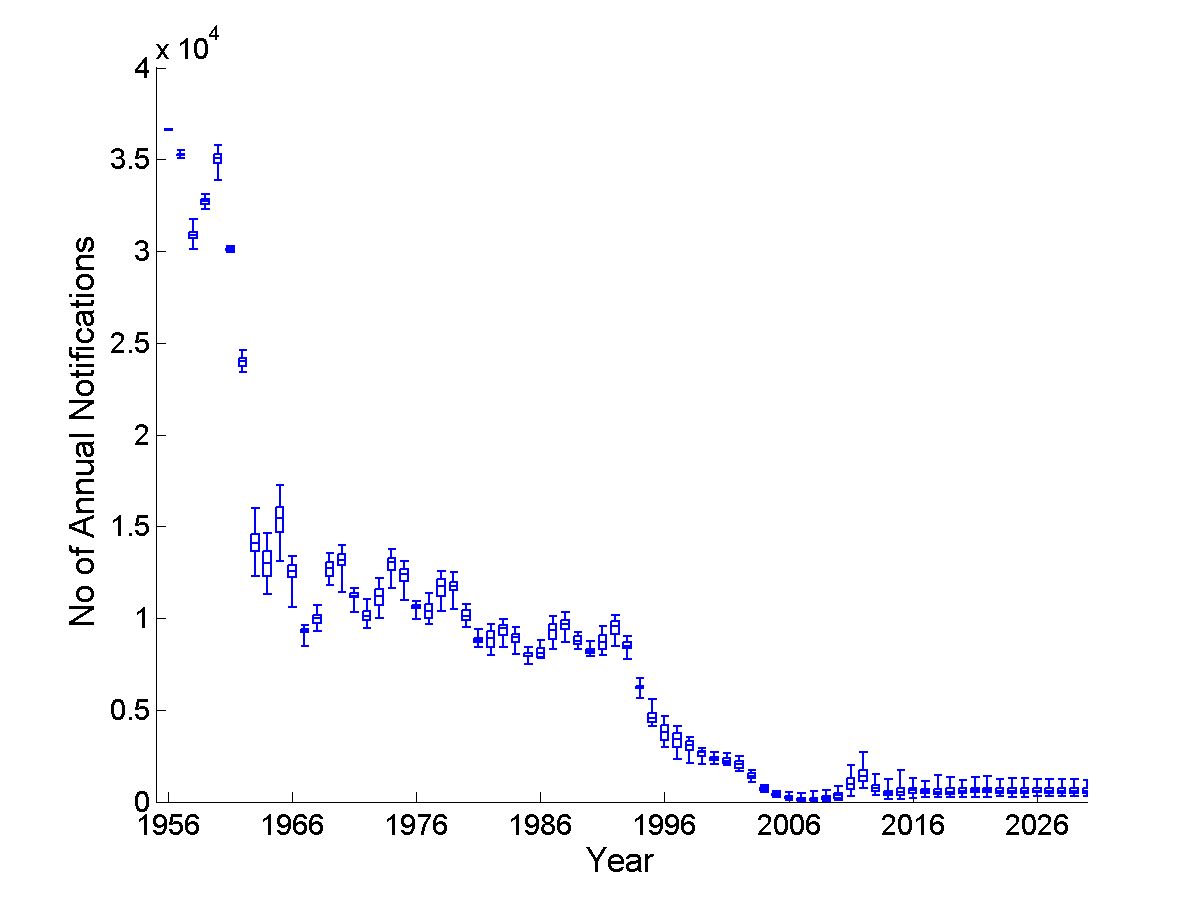

Supplement: Additional file 3: — Graphical User Interface porgramme to present pertussis simulation model results. (ZIP 8235 kb) [file 12916_2016_665_MOESM3_ESM.zip › POLYMOD_GUI_3_4.tif]

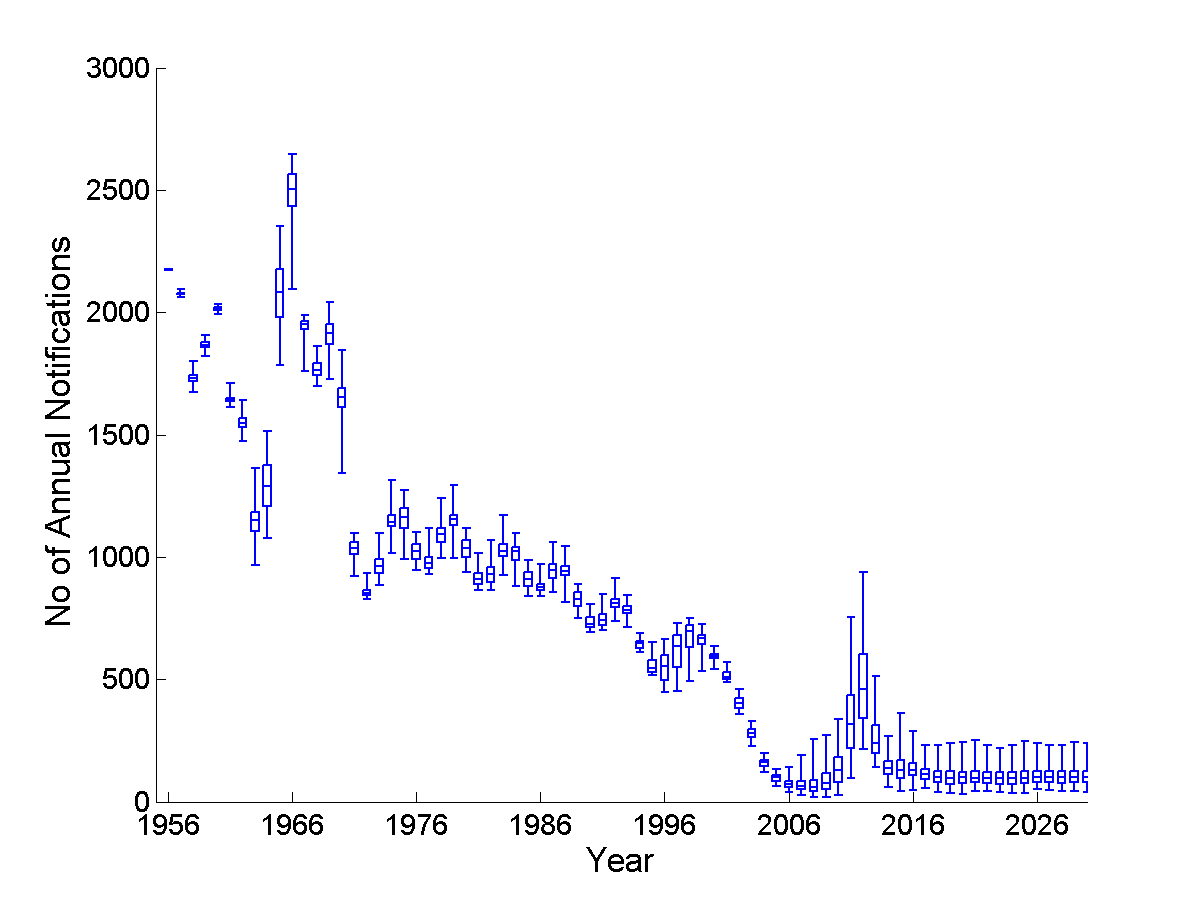

Supplement: Additional file 3: — Graphical User Interface porgramme to present pertussis simulation model results. (ZIP 8235 kb) [file 12916_2016_665_MOESM3_ESM.zip › POLYMOD_GUI_3_5.tif]

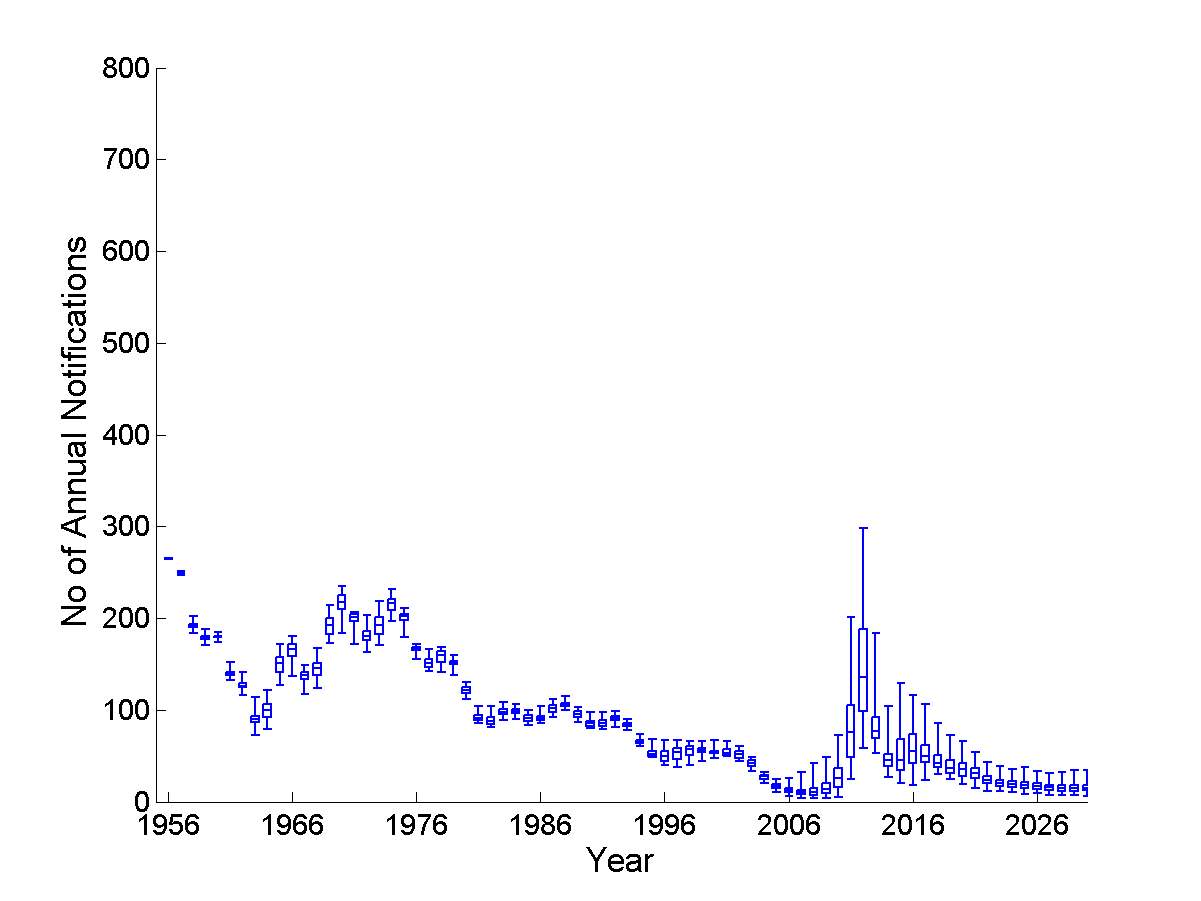

Supplement: Additional file 3: — Graphical User Interface porgramme to present pertussis simulation model results. (ZIP 8235 kb) [file 12916_2016_665_MOESM3_ESM.zip › POLYMOD_GUI_3_6.tif]

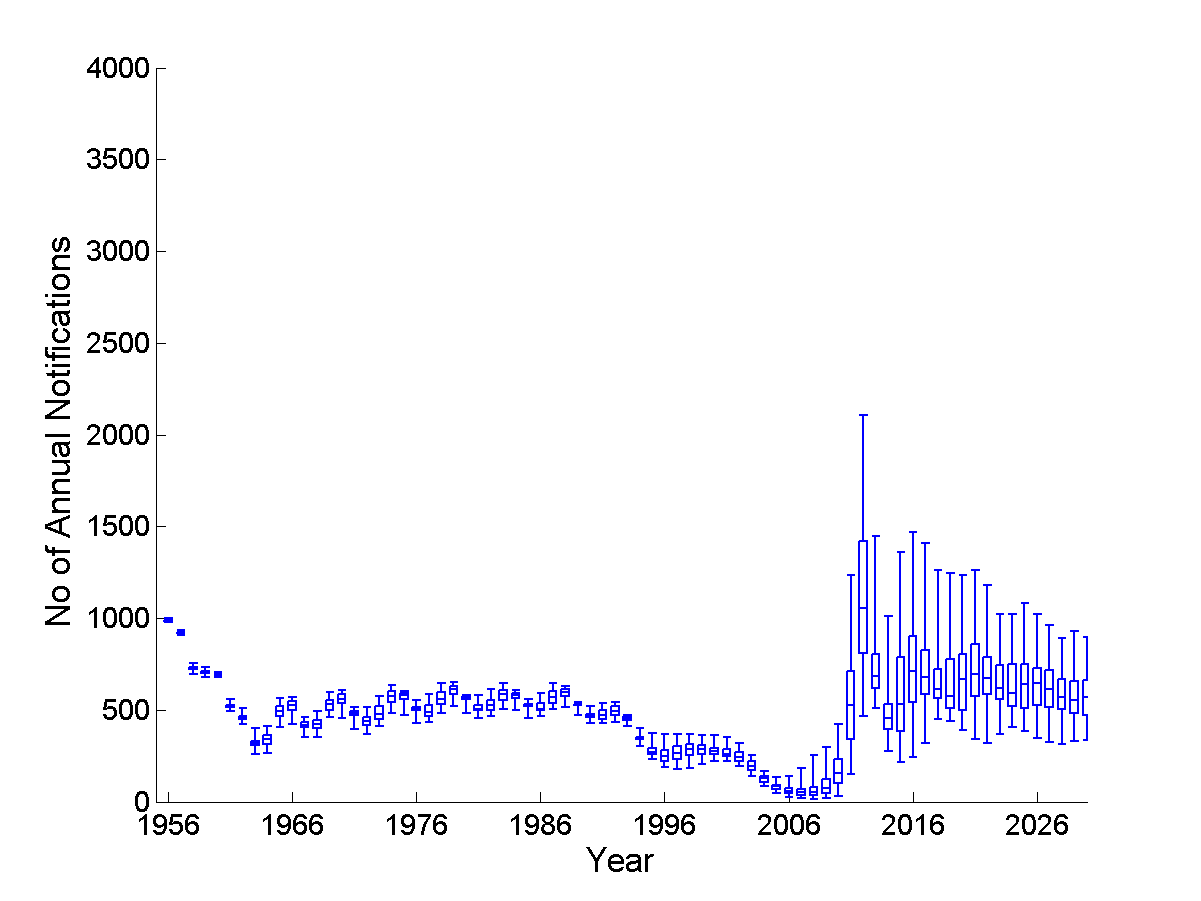

Supplement: Additional file 3: — Graphical User Interface porgramme to present pertussis simulation model results. (ZIP 8235 kb) [file 12916_2016_665_MOESM3_ESM.zip › POLYMOD_GUI_3_7.tif]

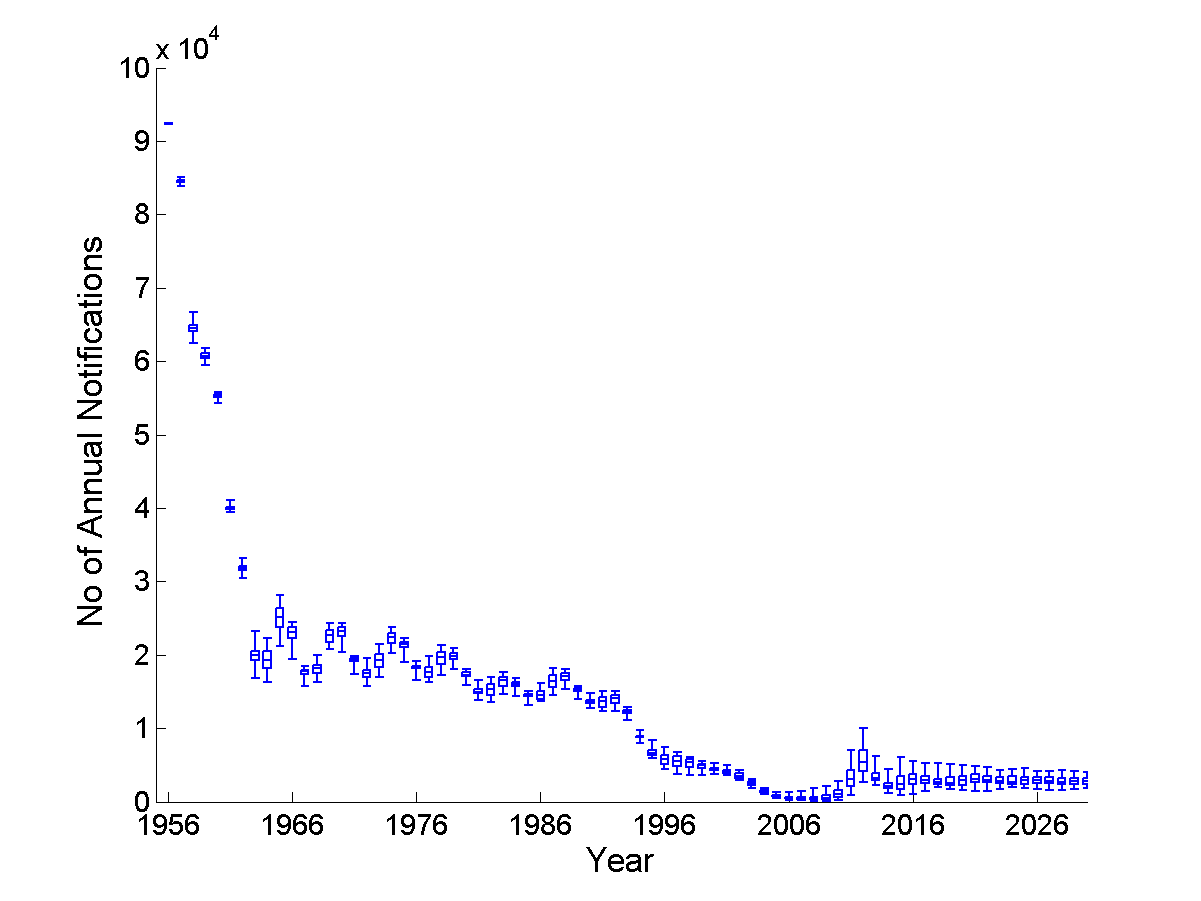

Supplement: Additional file 3: — Graphical User Interface porgramme to present pertussis simulation model results. (ZIP 8235 kb) [file 12916_2016_665_MOESM3_ESM.zip › POLYMOD_GUI_3_8.tif]

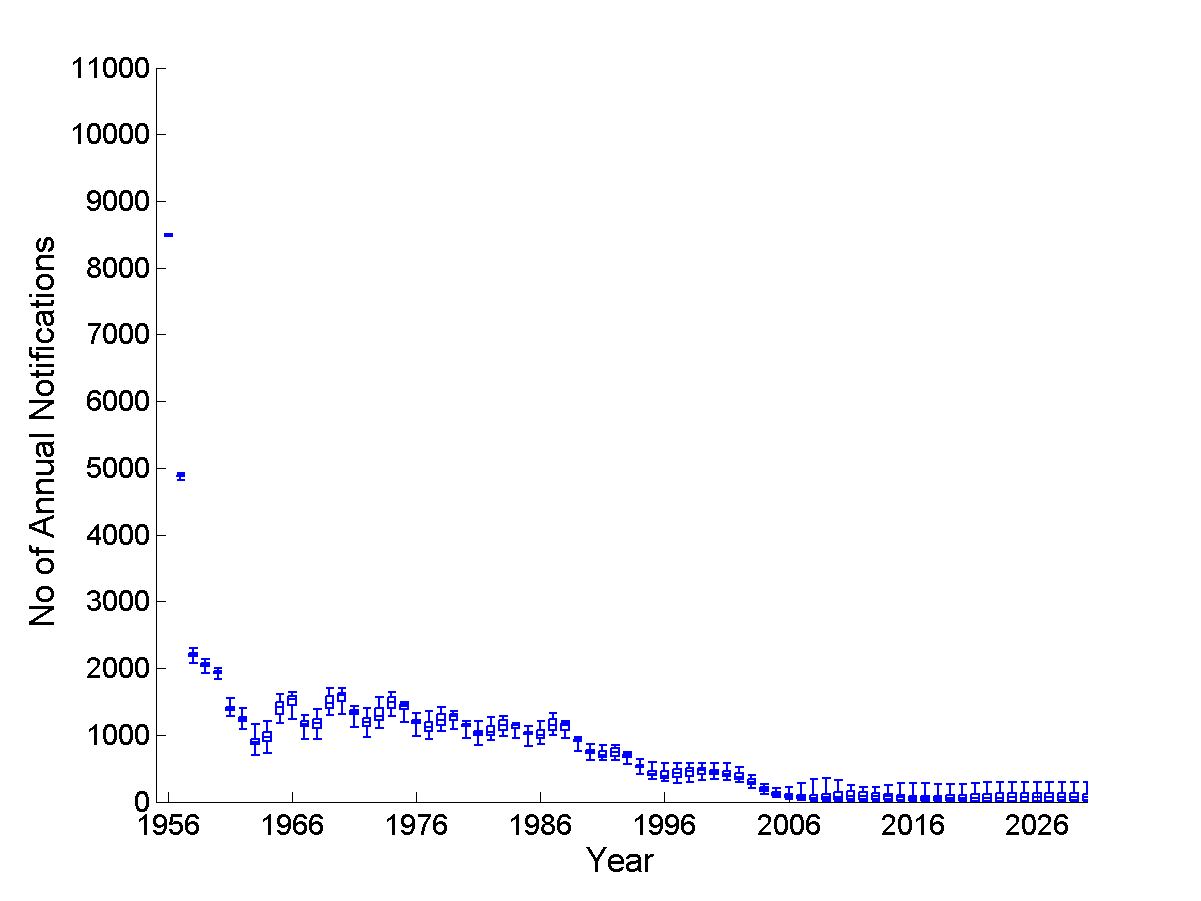

Supplement: Additional file 3: — Graphical User Interface porgramme to present pertussis simulation model results. (ZIP 8235 kb) [file 12916_2016_665_MOESM3_ESM.zip › POLYMOD_GUI_4_1.tif]

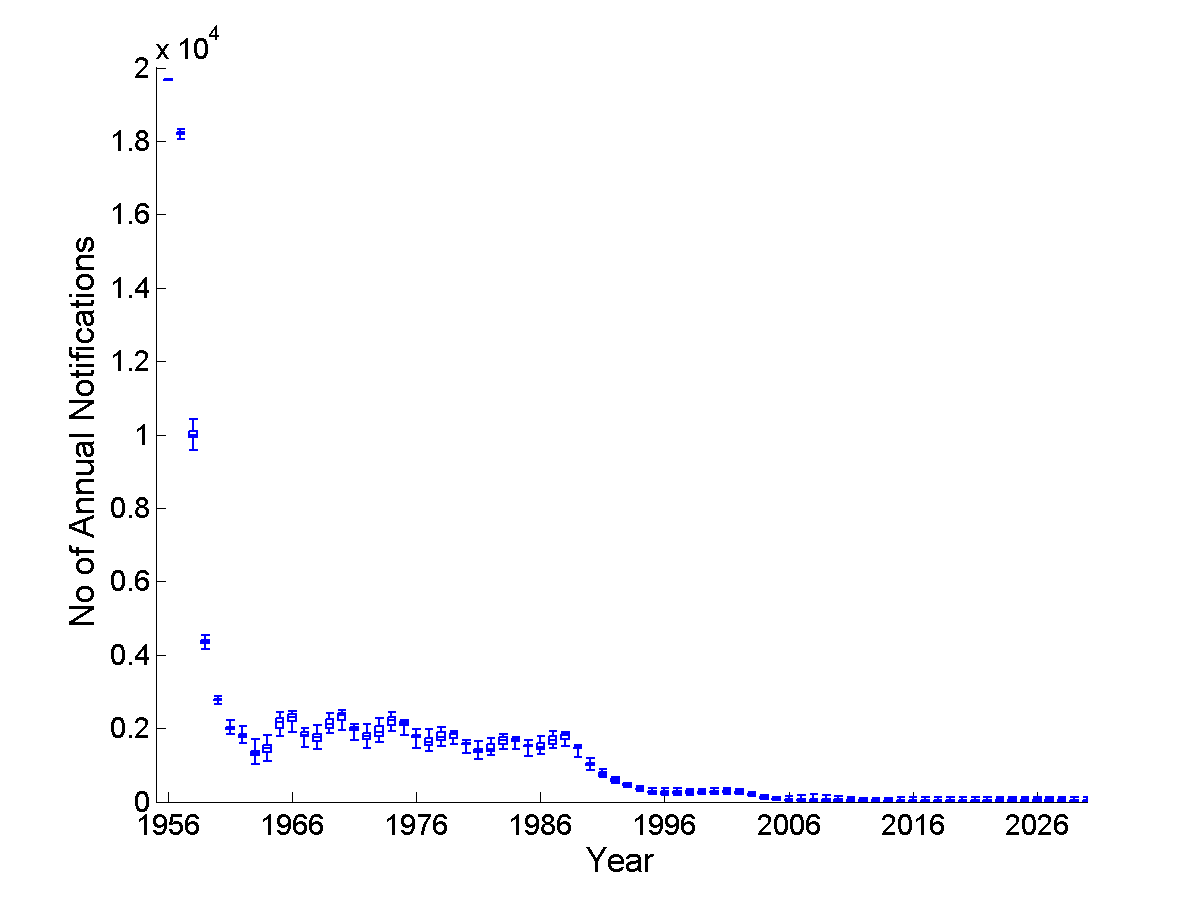

Supplement: Additional file 3: — Graphical User Interface porgramme to present pertussis simulation model results. (ZIP 8235 kb) [file 12916_2016_665_MOESM3_ESM.zip › POLYMOD_GUI_4_2.tif]

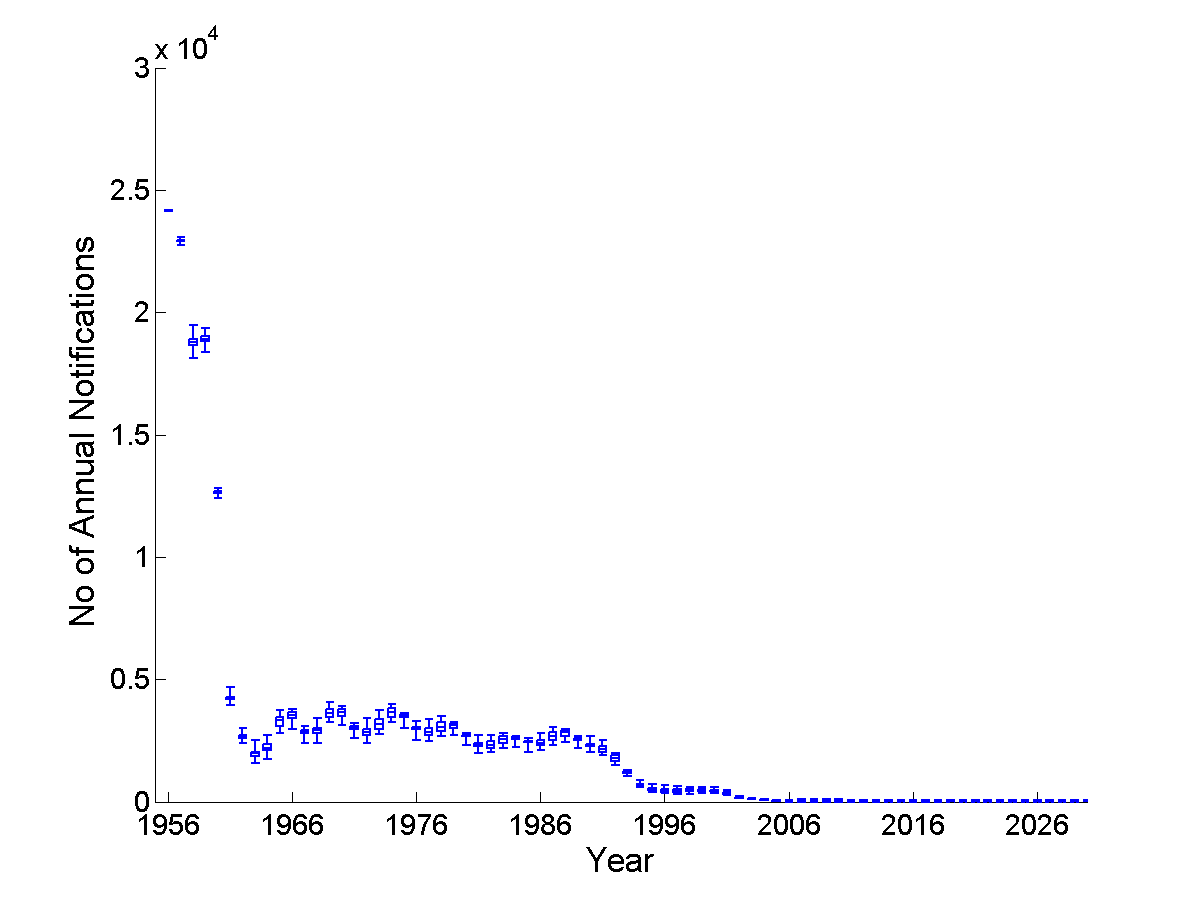

Supplement: Additional file 3: — Graphical User Interface porgramme to present pertussis simulation model results. (ZIP 8235 kb) [file 12916_2016_665_MOESM3_ESM.zip › POLYMOD_GUI_4_3.tif]

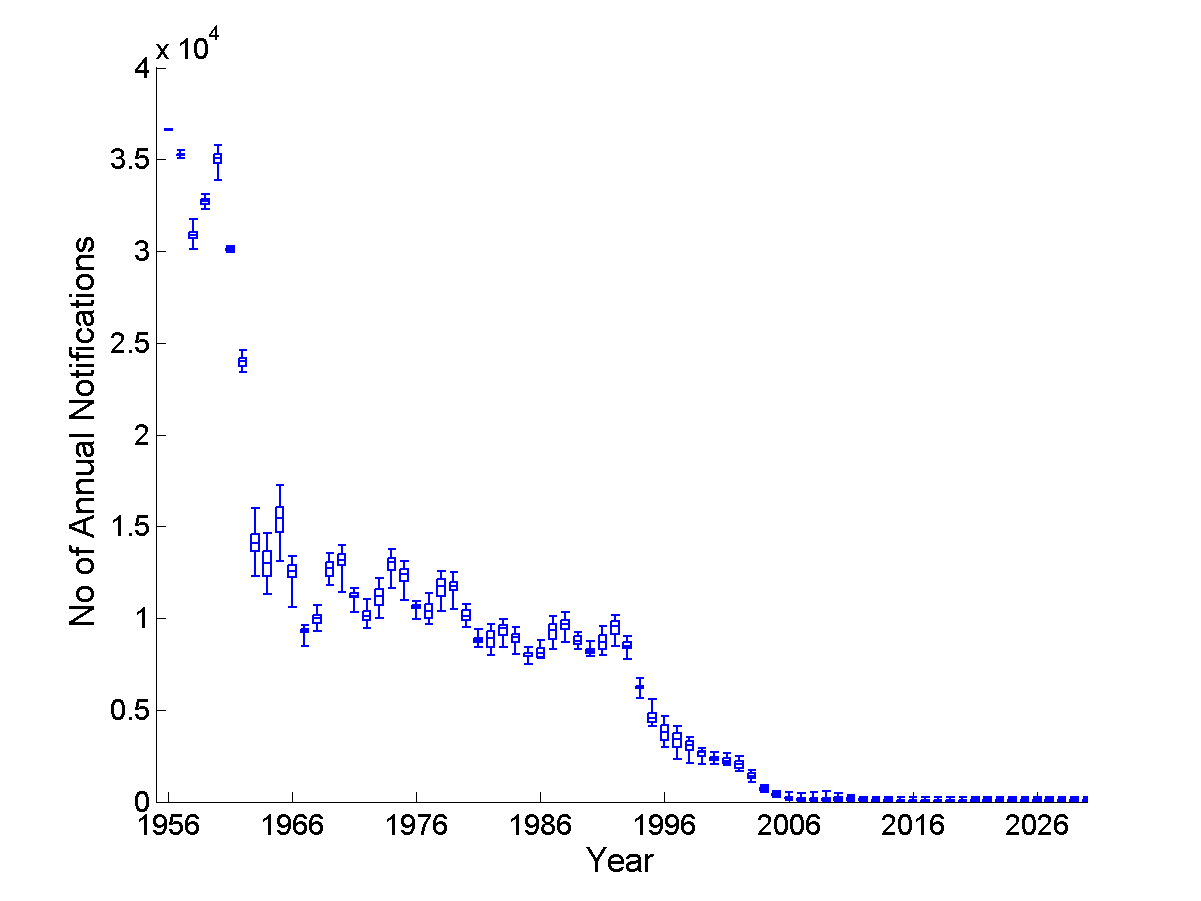

Supplement: Additional file 3: — Graphical User Interface porgramme to present pertussis simulation model results. (ZIP 8235 kb) [file 12916_2016_665_MOESM3_ESM.zip › POLYMOD_GUI_4_4.tif]

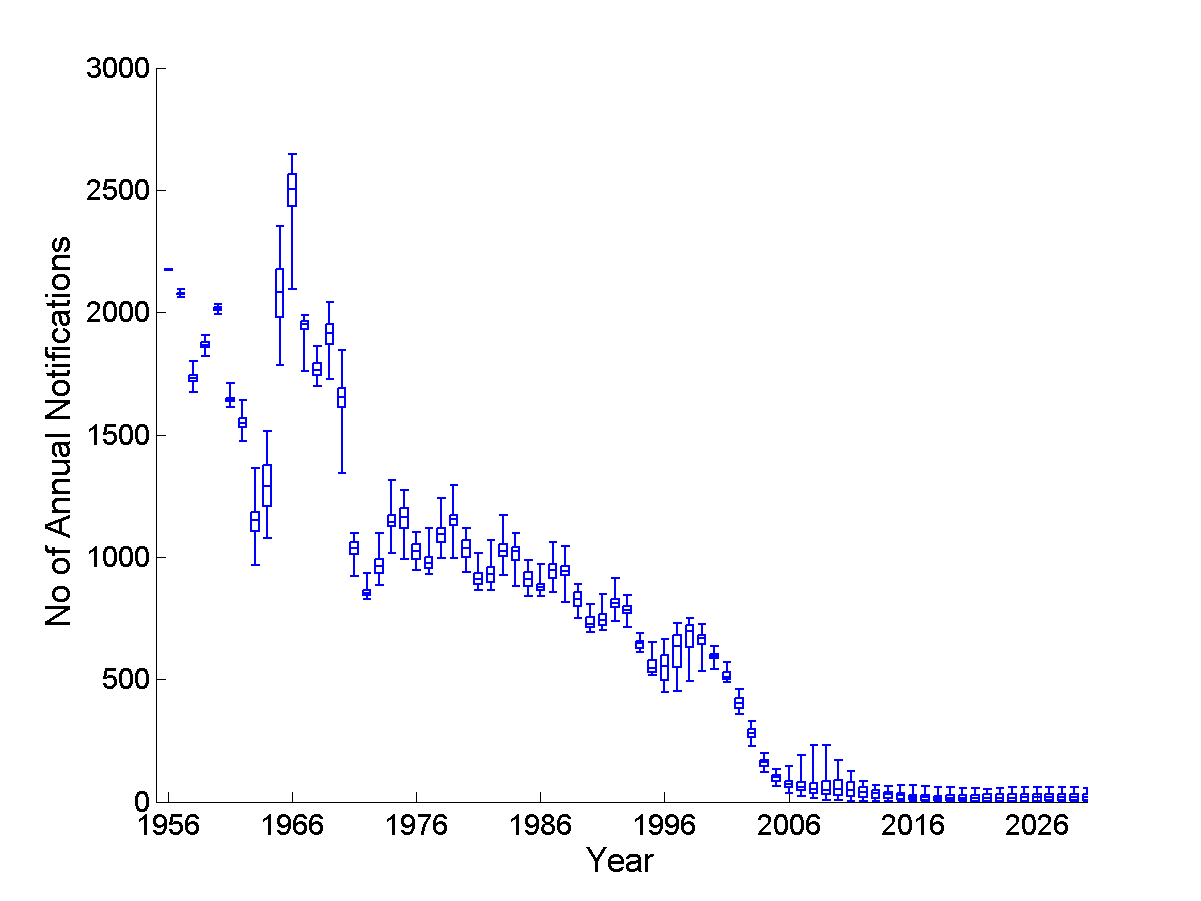

Supplement: Additional file 3: — Graphical User Interface porgramme to present pertussis simulation model results. (ZIP 8235 kb) [file 12916_2016_665_MOESM3_ESM.zip › POLYMOD_GUI_4_5.tif]

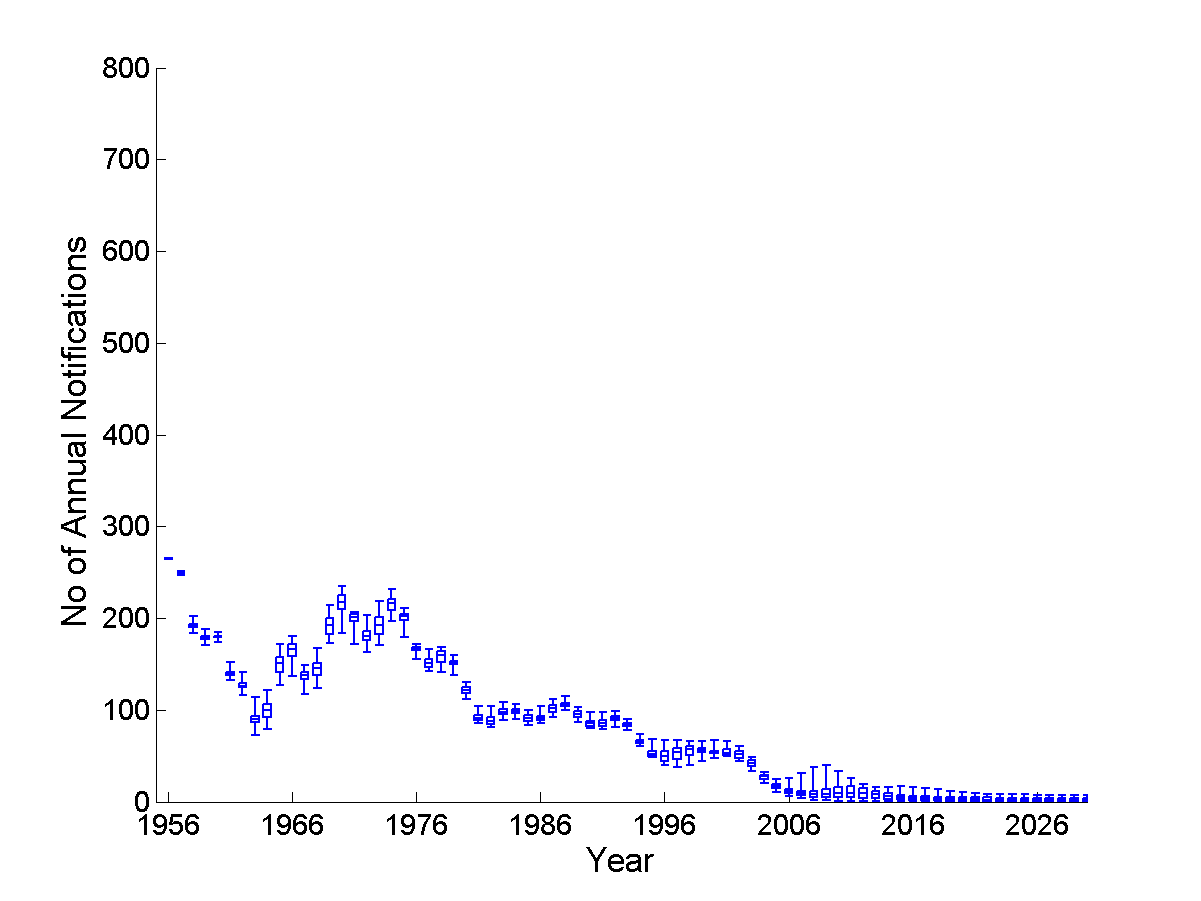

Supplement: Additional file 3: — Graphical User Interface porgramme to present pertussis simulation model results. (ZIP 8235 kb) [file 12916_2016_665_MOESM3_ESM.zip › POLYMOD_GUI_4_6.tif]

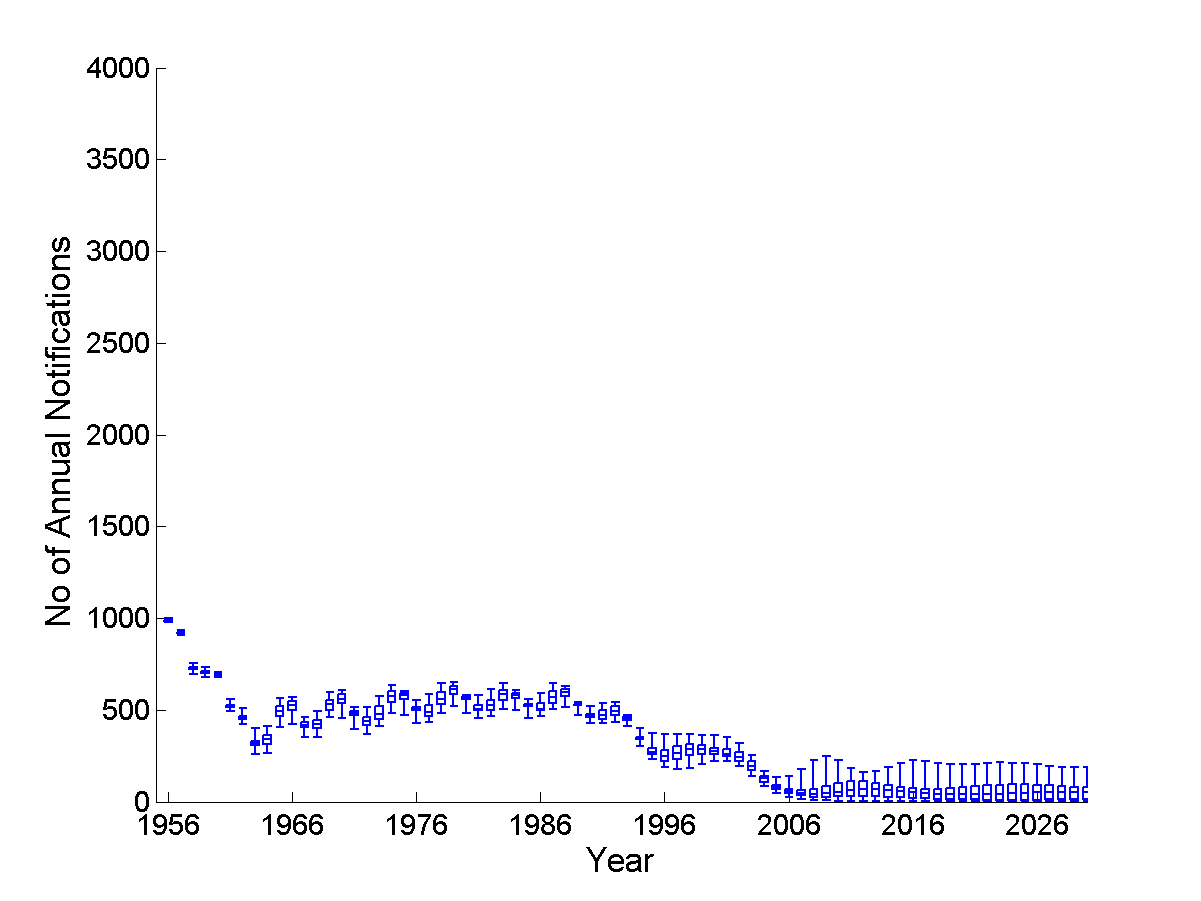

Supplement: Additional file 3: — Graphical User Interface porgramme to present pertussis simulation model results. (ZIP 8235 kb) [file 12916_2016_665_MOESM3_ESM.zip › POLYMOD_GUI_4_7.tif]

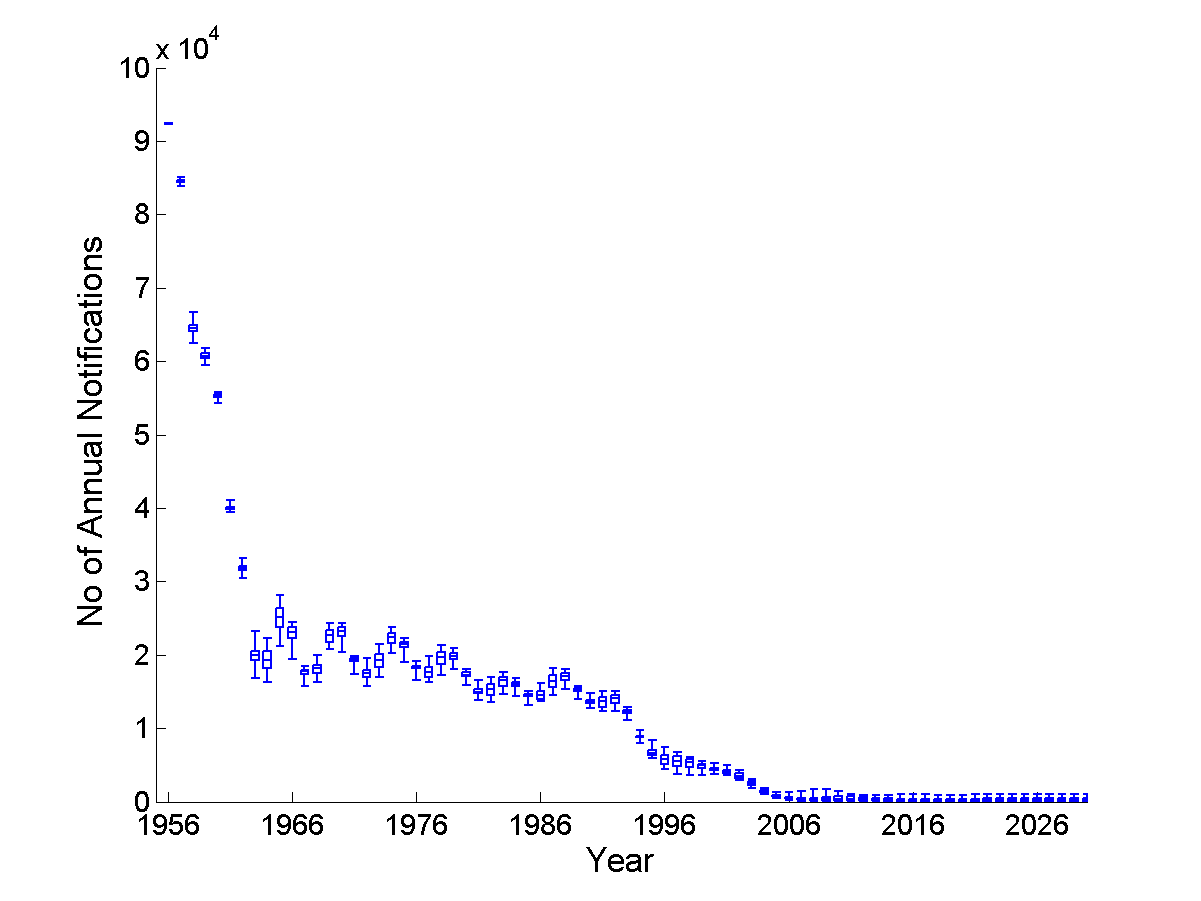

Supplement: Additional file 3: — Graphical User Interface porgramme to present pertussis simulation model results. (ZIP 8235 kb) [file 12916_2016_665_MOESM3_ESM.zip › POLYMOD_GUI_4_8.tif]

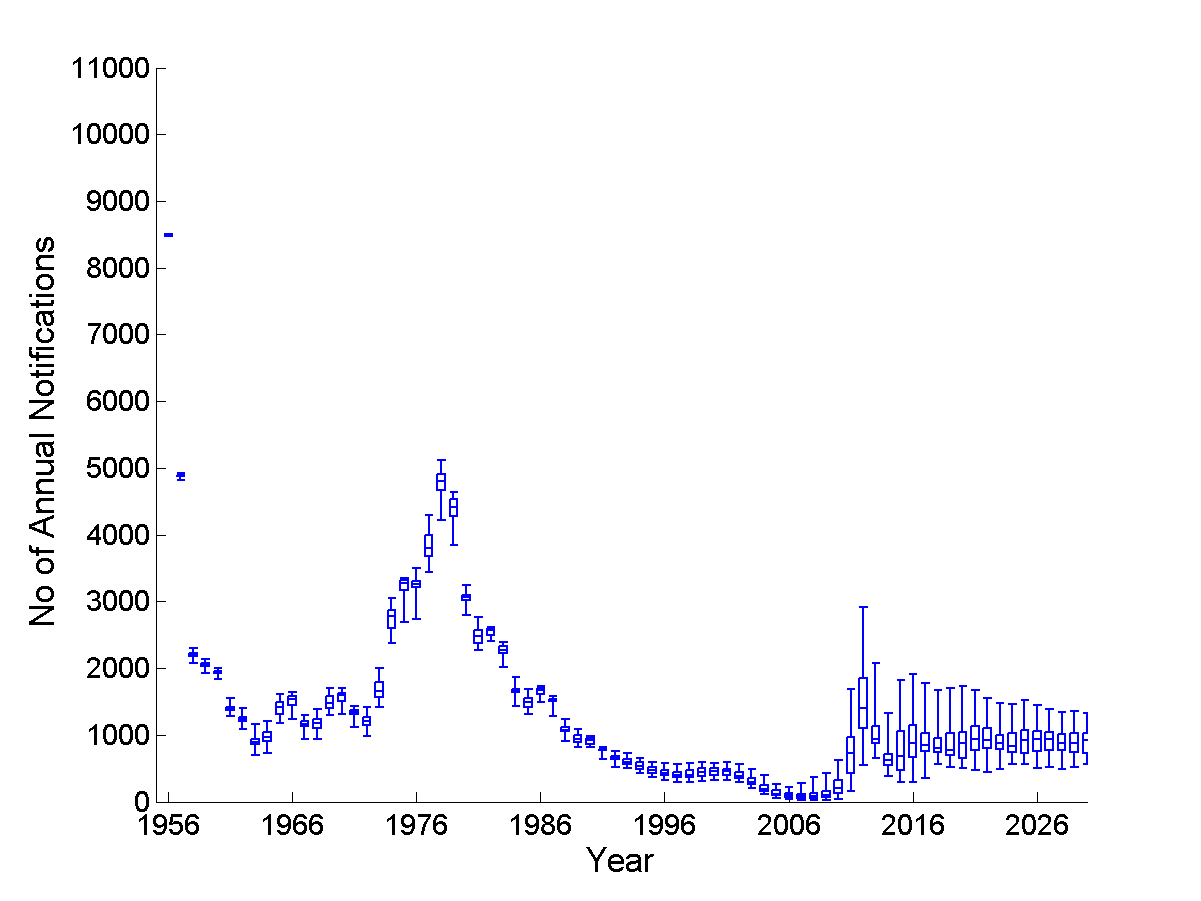

Supplement: Additional file 3: — Graphical User Interface porgramme to present pertussis simulation model results. (ZIP 8235 kb) [file 12916_2016_665_MOESM3_ESM.zip › POLYMOD_GUI_5_1.tif]

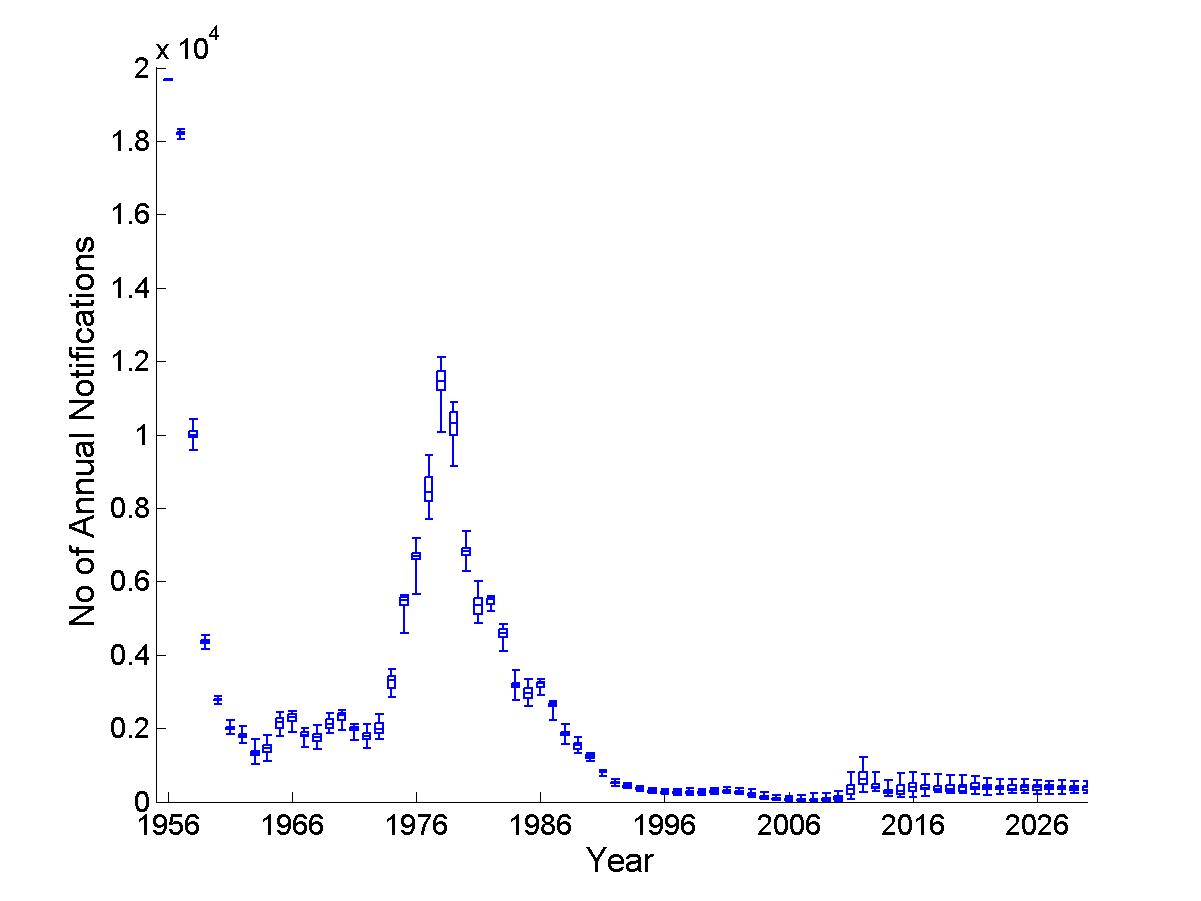

Supplement: Additional file 3: — Graphical User Interface porgramme to present pertussis simulation model results. (ZIP 8235 kb) [file 12916_2016_665_MOESM3_ESM.zip › POLYMOD_GUI_5_2.tif]

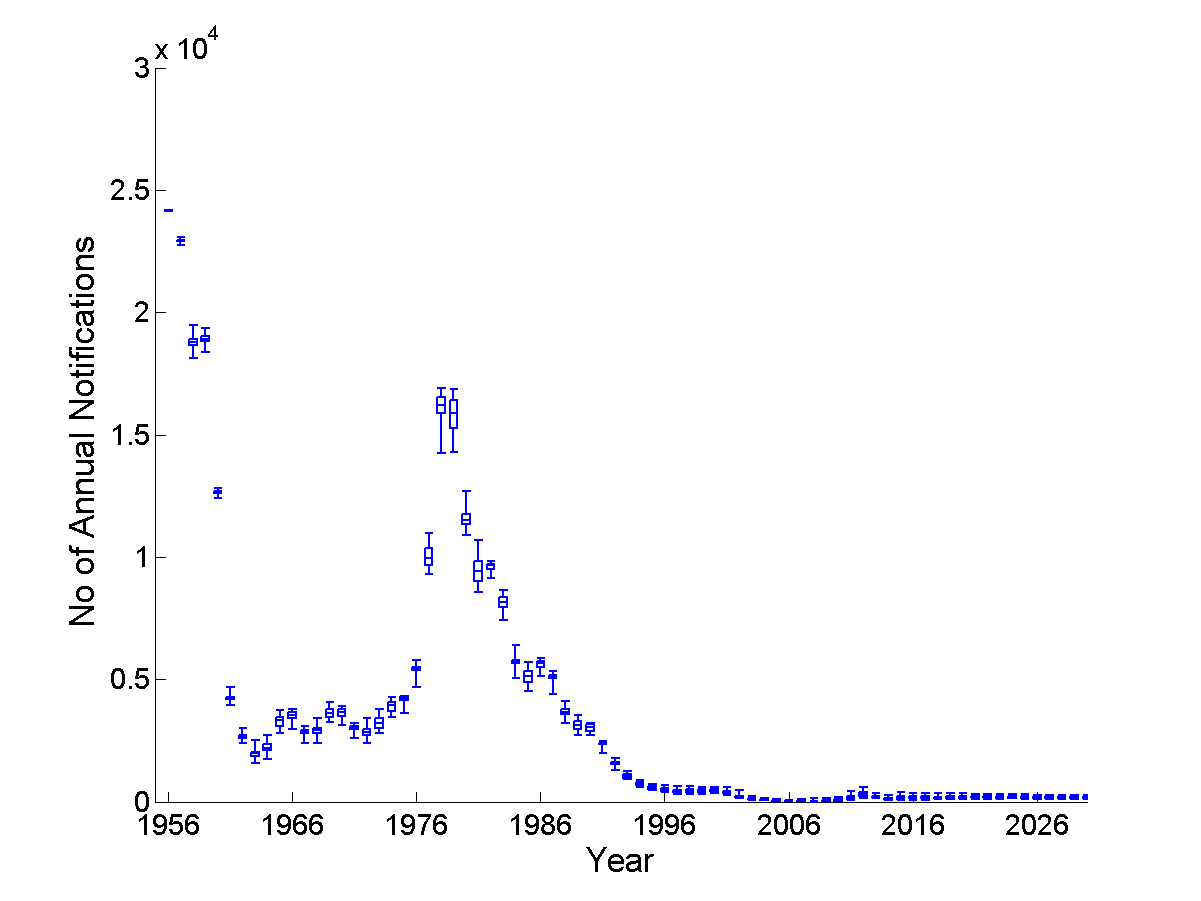

Supplement: Additional file 3: — Graphical User Interface porgramme to present pertussis simulation model results. (ZIP 8235 kb) [file 12916_2016_665_MOESM3_ESM.zip › POLYMOD_GUI_5_3.tif]

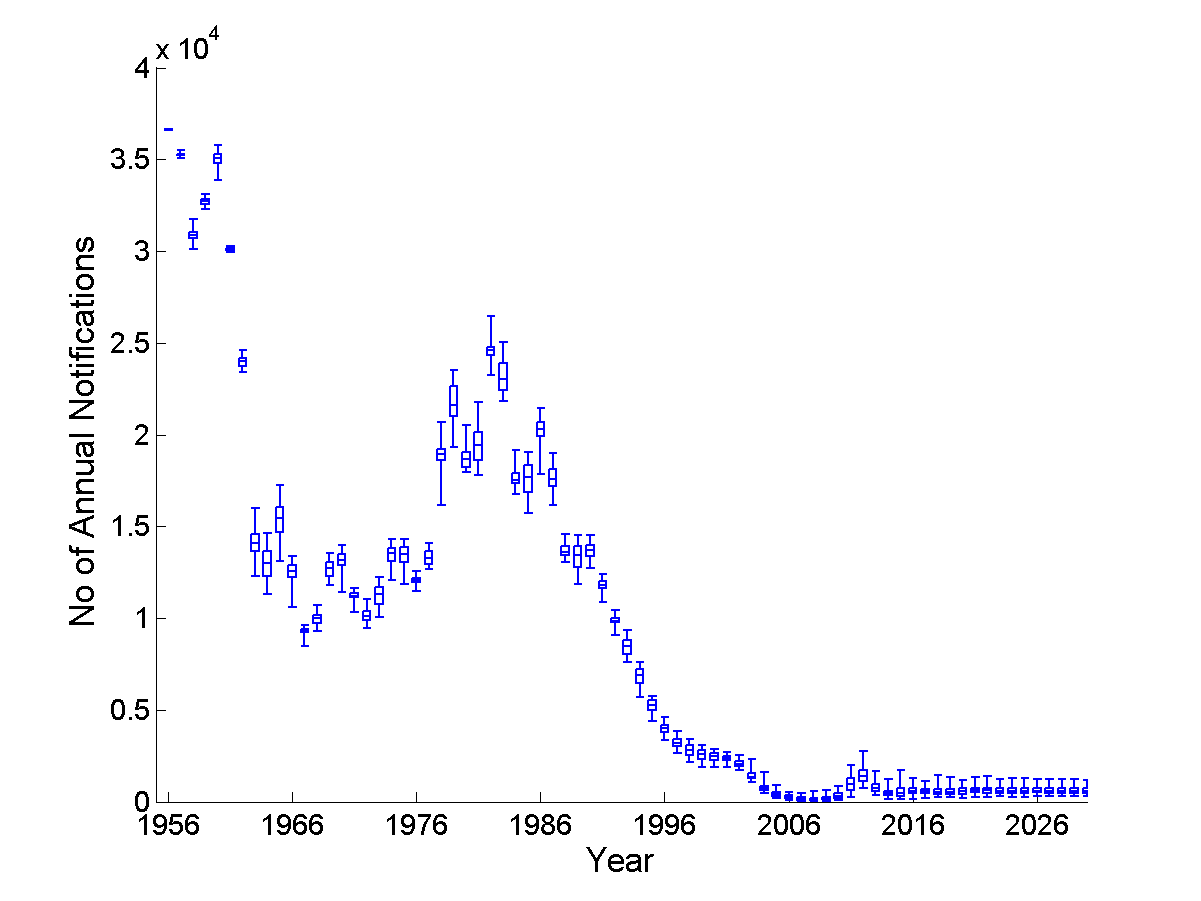

Supplement: Additional file 3: — Graphical User Interface porgramme to present pertussis simulation model results. (ZIP 8235 kb) [file 12916_2016_665_MOESM3_ESM.zip › POLYMOD_GUI_5_4.tif]

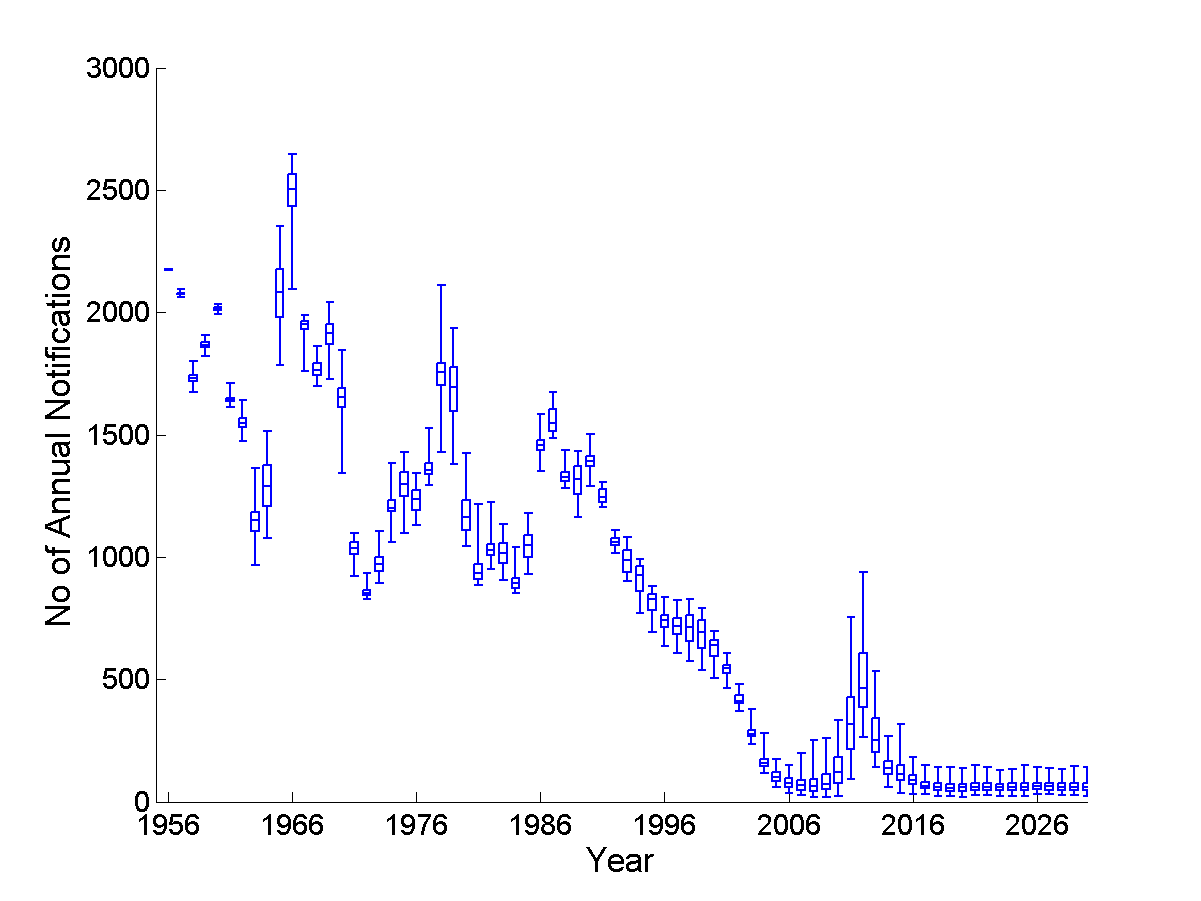

Supplement: Additional file 3: — Graphical User Interface porgramme to present pertussis simulation model results. (ZIP 8235 kb) [file 12916_2016_665_MOESM3_ESM.zip › POLYMOD_GUI_5_5.tif]

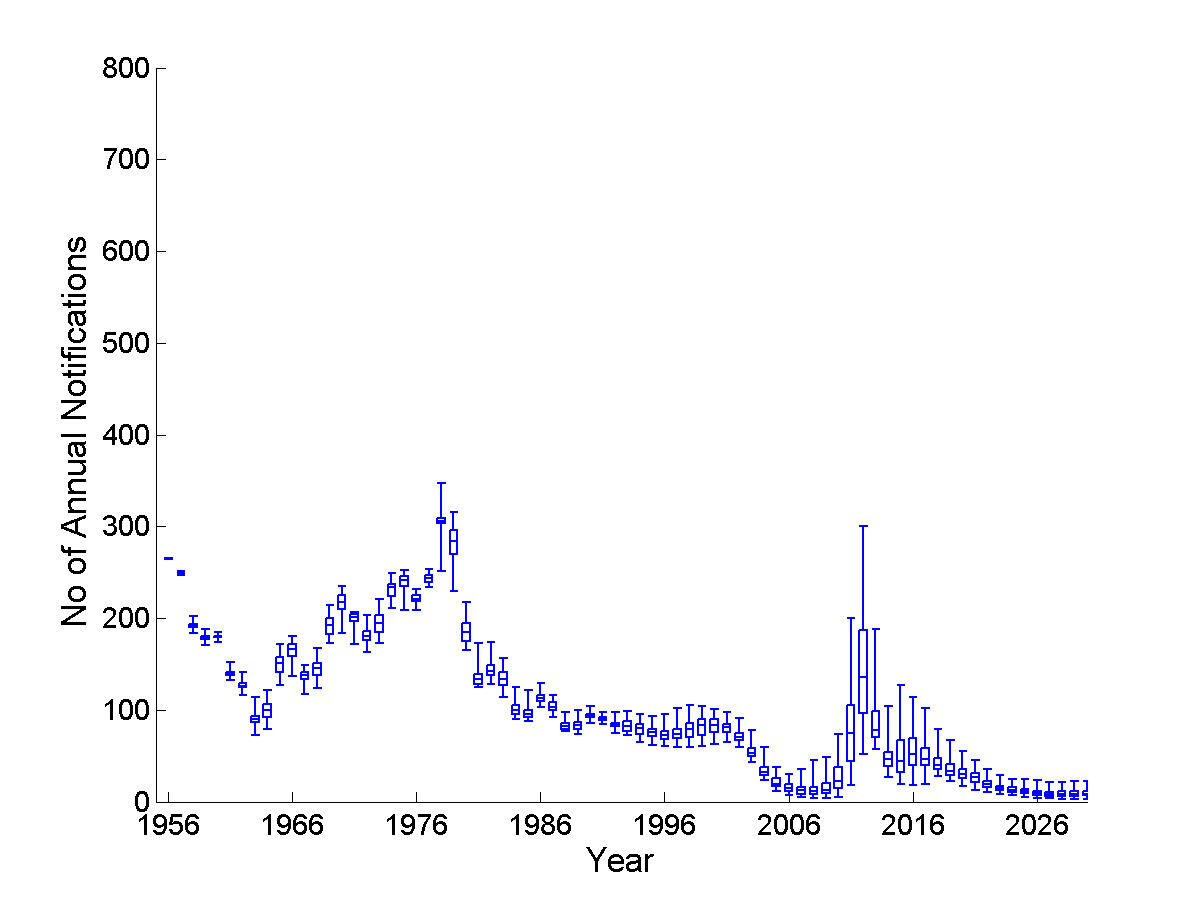

Supplement: Additional file 3: — Graphical User Interface porgramme to present pertussis simulation model results. (ZIP 8235 kb) [file 12916_2016_665_MOESM3_ESM.zip › POLYMOD_GUI_5_6.tif]

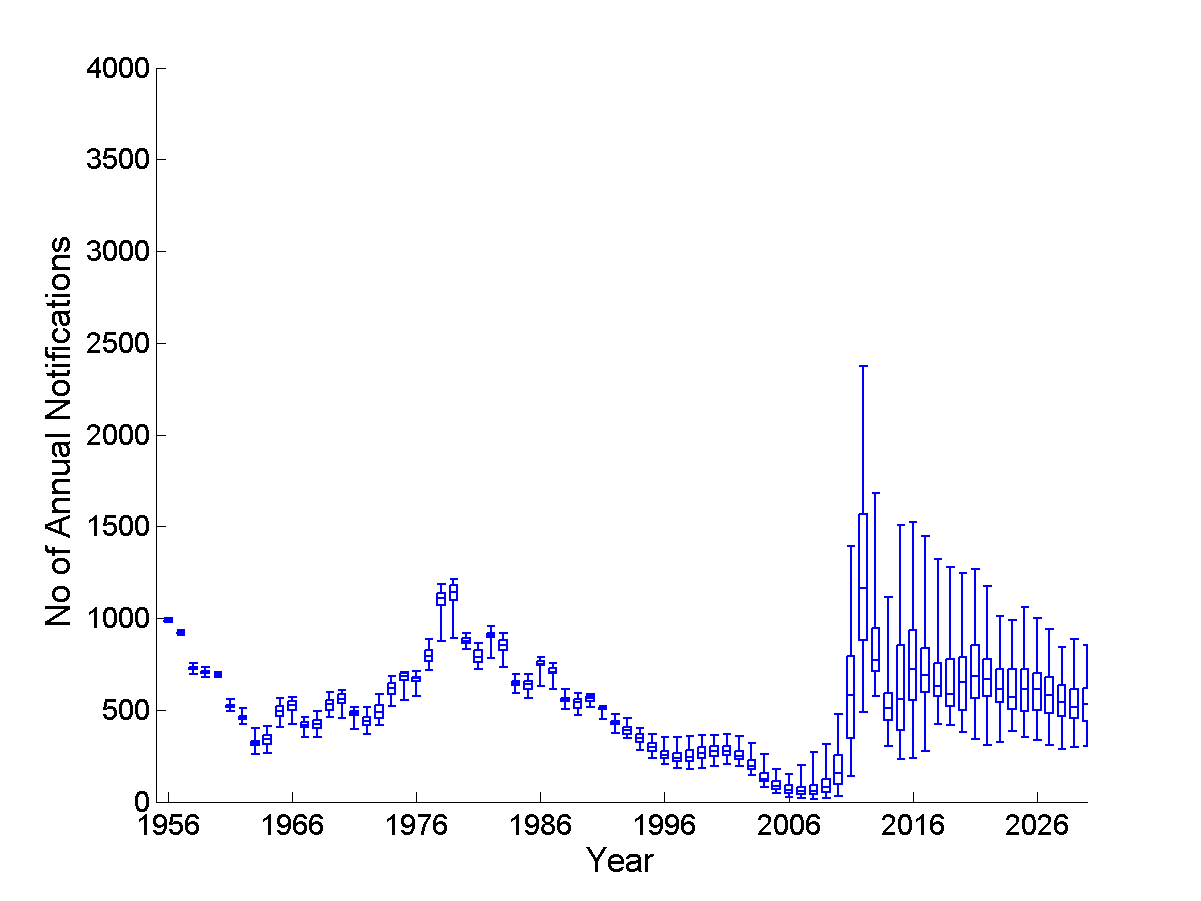

Supplement: Additional file 3: — Graphical User Interface porgramme to present pertussis simulation model results. (ZIP 8235 kb) [file 12916_2016_665_MOESM3_ESM.zip › POLYMOD_GUI_5_7.tif]

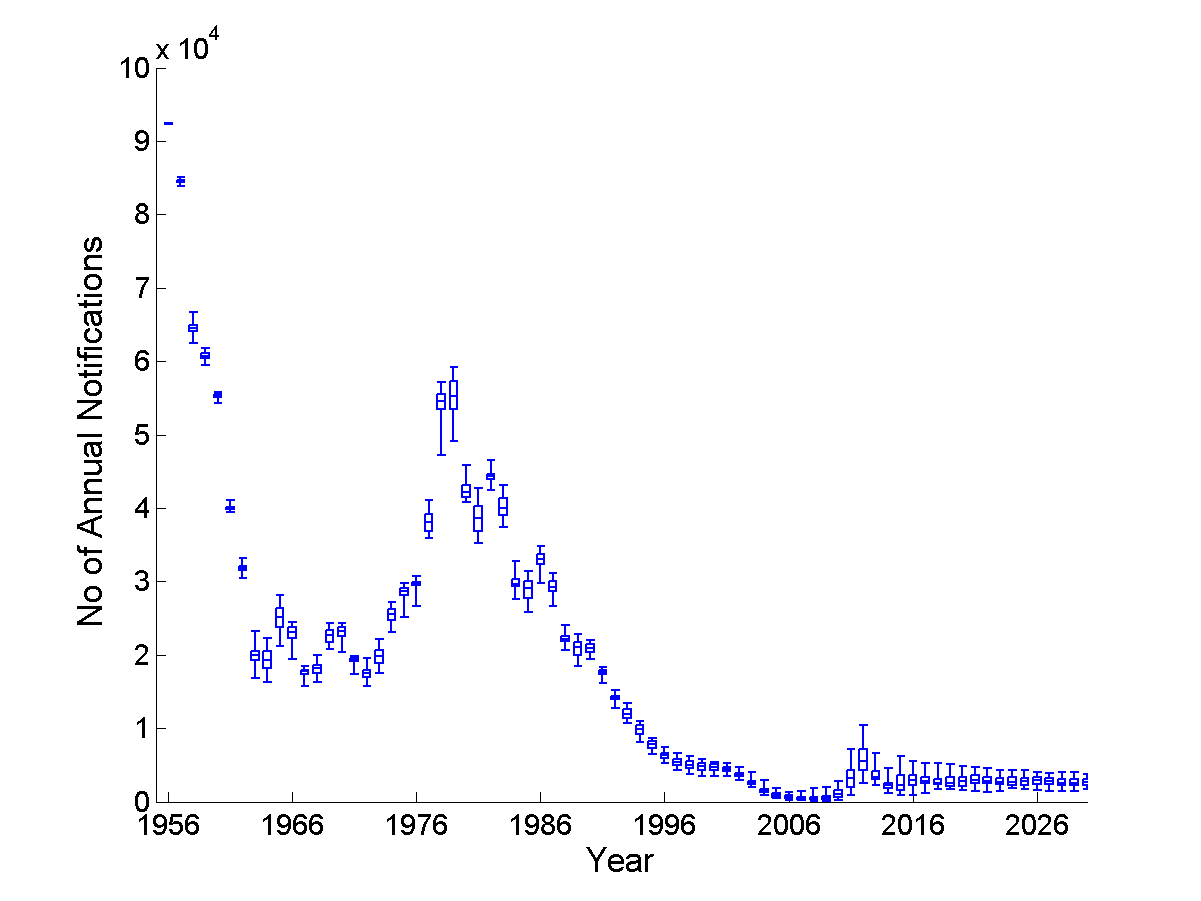

Supplement: Additional file 3: — Graphical User Interface porgramme to present pertussis simulation model results. (ZIP 8235 kb) [file 12916_2016_665_MOESM3_ESM.zip › POLYMOD_GUI_5_8.tif]

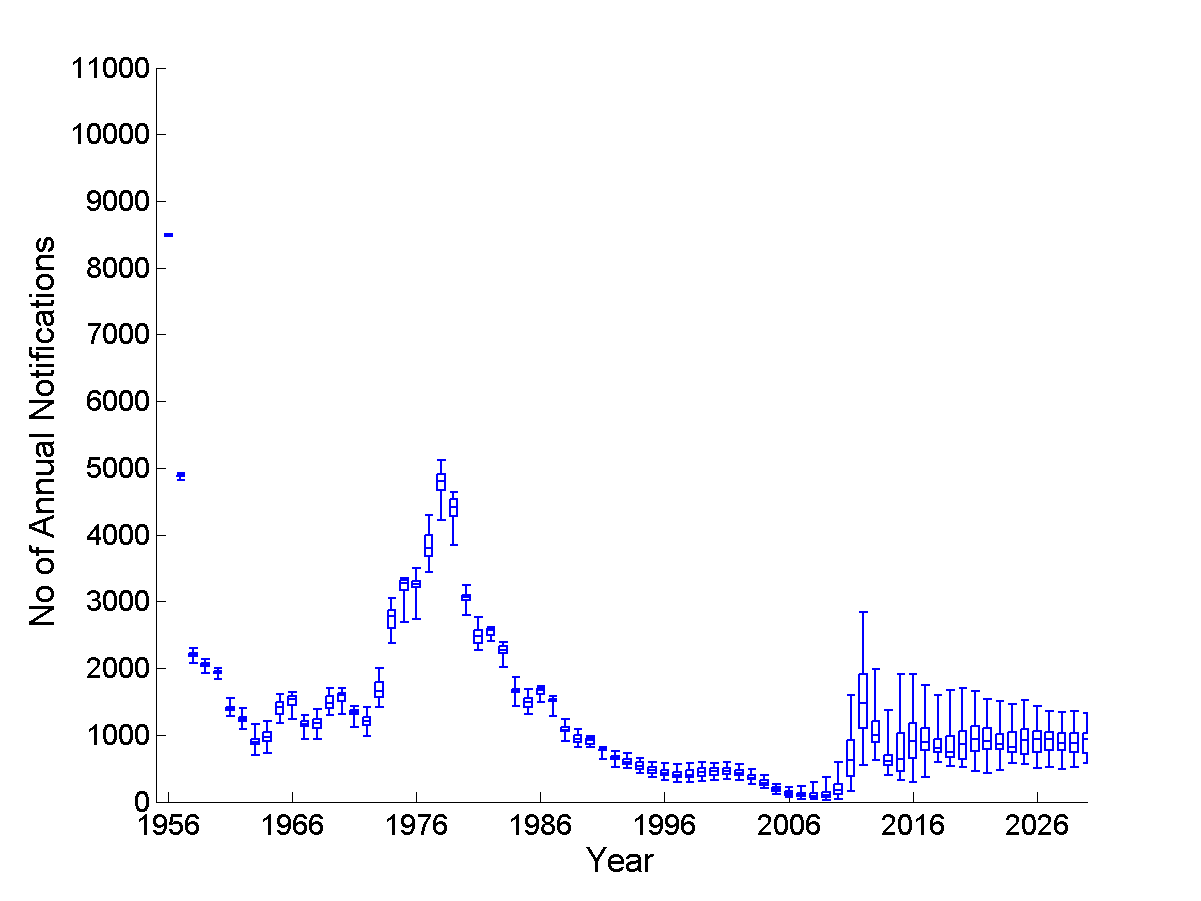

Supplement: Additional file 3: — Graphical User Interface porgramme to present pertussis simulation model results. (ZIP 8235 kb) [file 12916_2016_665_MOESM3_ESM.zip › POLYMOD_GUI_6A_1.tif]

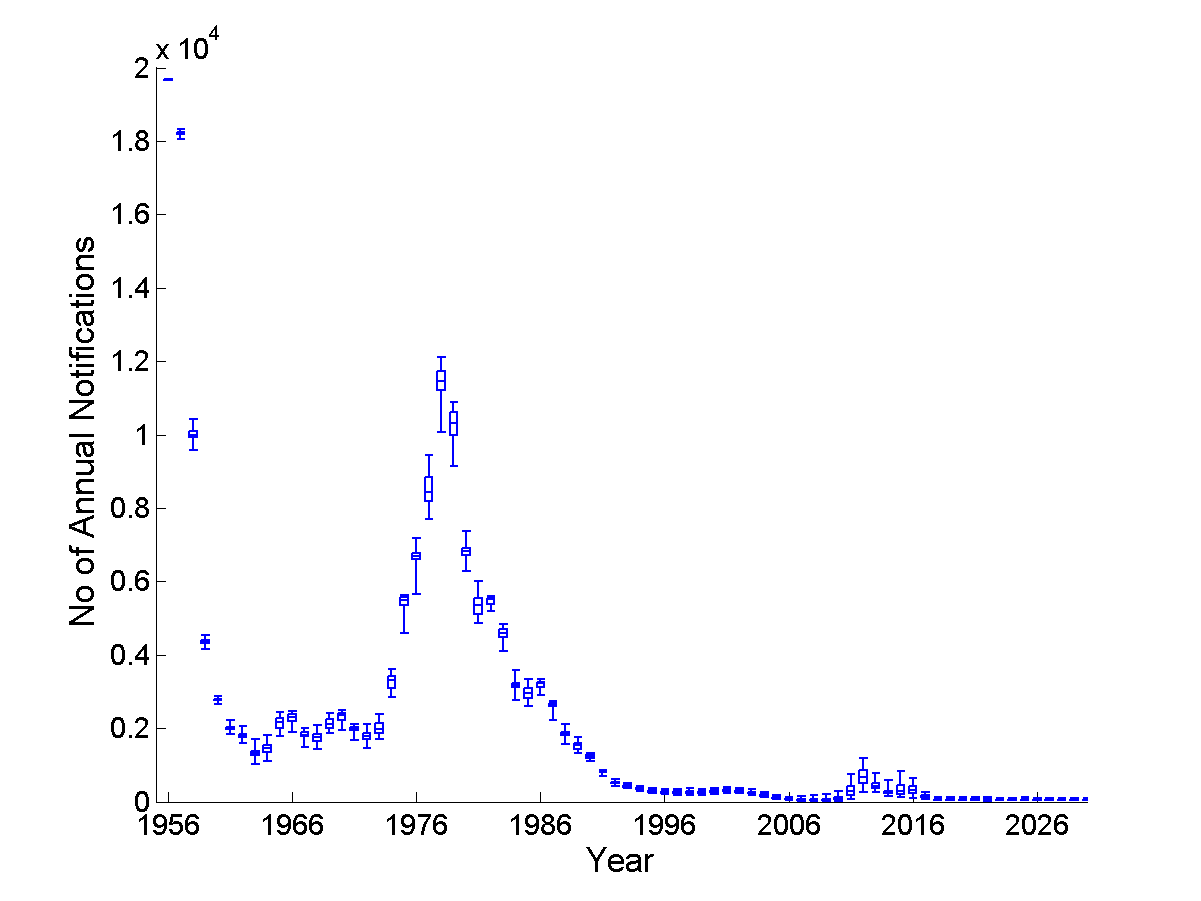

Supplement: Additional file 3: — Graphical User Interface porgramme to present pertussis simulation model results. (ZIP 8235 kb) [file 12916_2016_665_MOESM3_ESM.zip › POLYMOD_GUI_6A_2.tif]

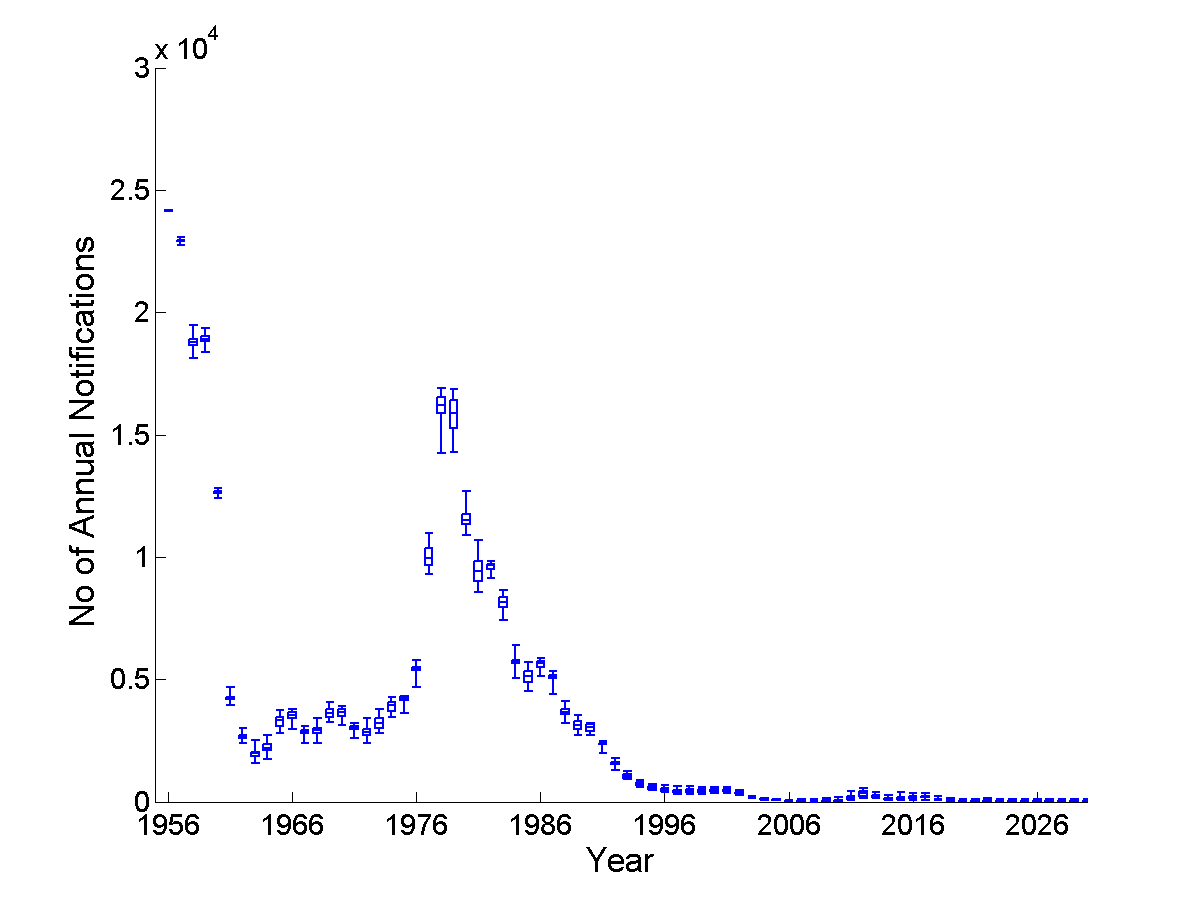

Supplement: Additional file 3: — Graphical User Interface porgramme to present pertussis simulation model results. (ZIP 8235 kb) [file 12916_2016_665_MOESM3_ESM.zip › POLYMOD_GUI_6A_3.tif]

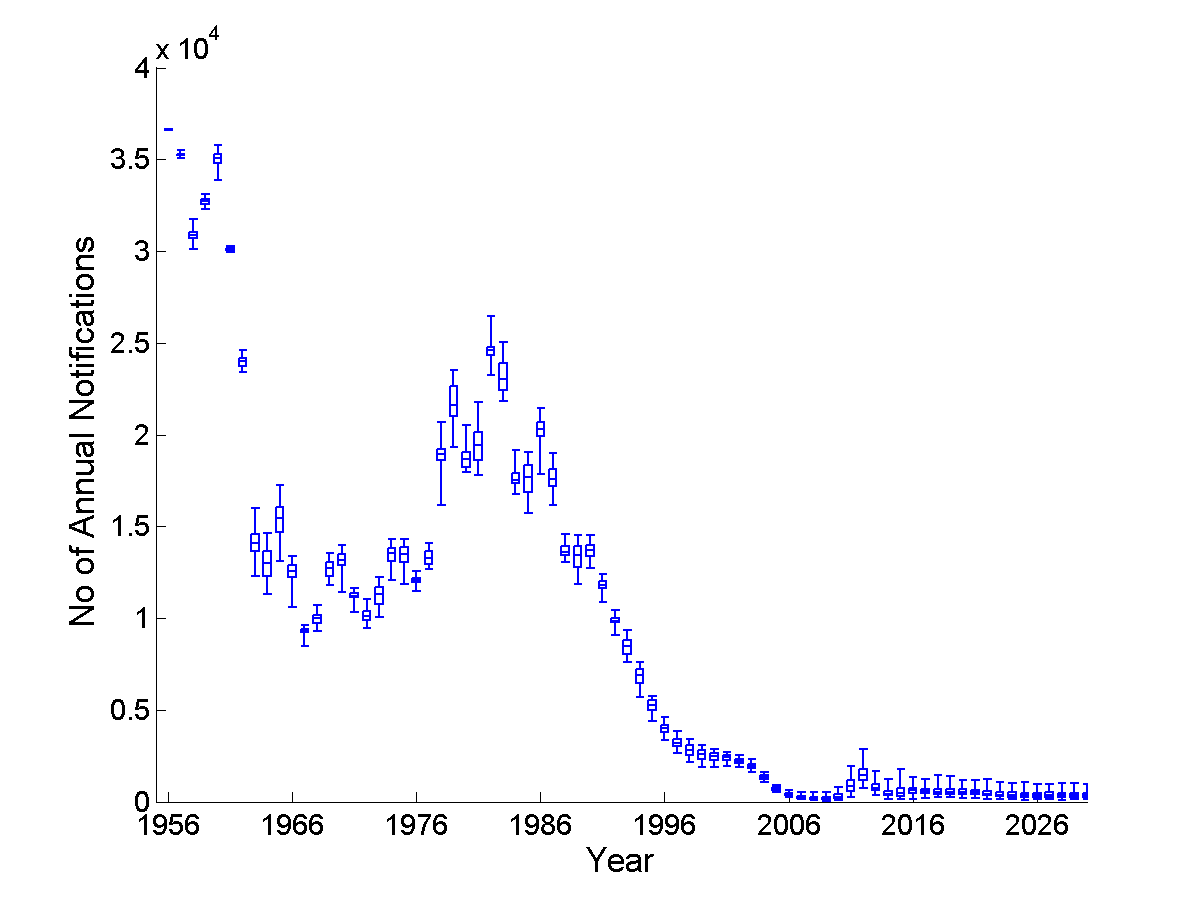

Supplement: Additional file 3: — Graphical User Interface porgramme to present pertussis simulation model results. (ZIP 8235 kb) [file 12916_2016_665_MOESM3_ESM.zip › POLYMOD_GUI_6A_4.tif]

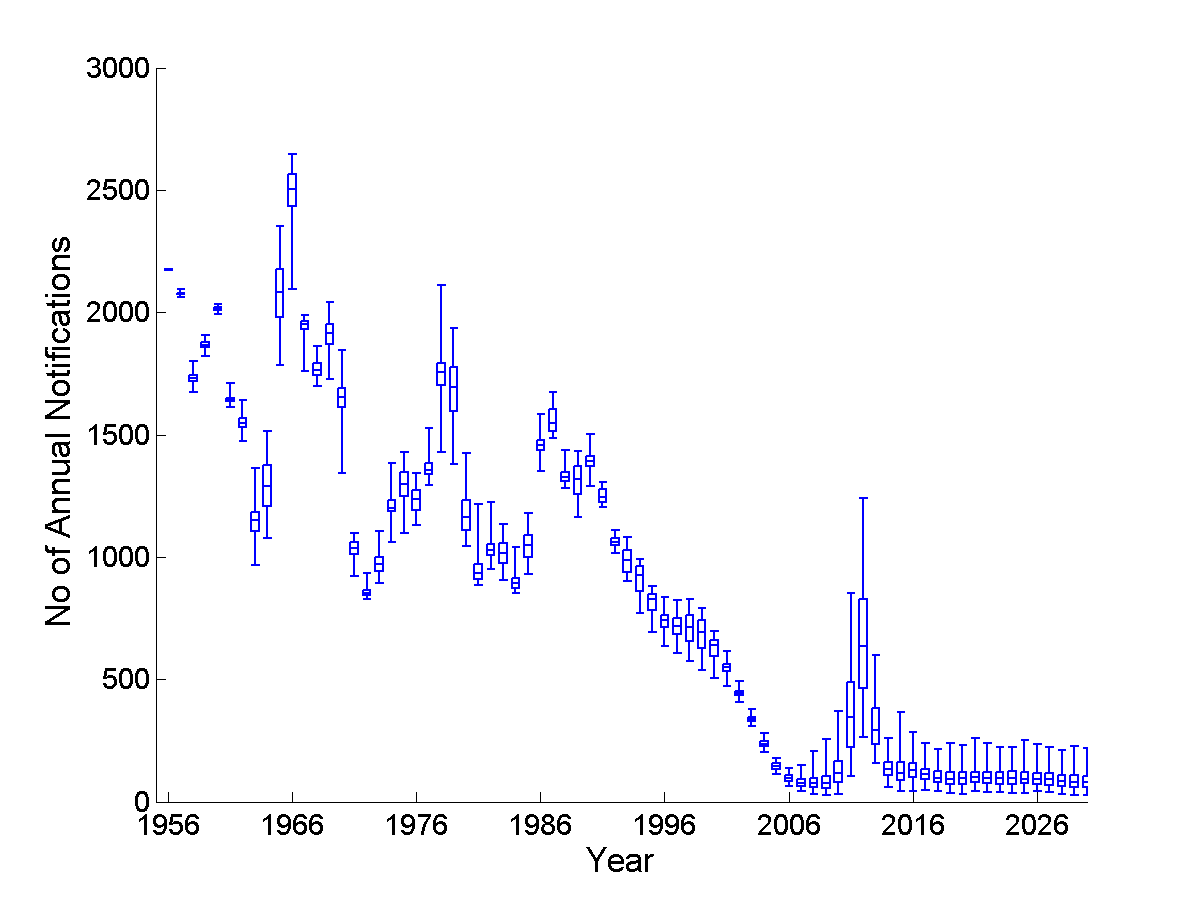

Supplement: Additional file 3: — Graphical User Interface porgramme to present pertussis simulation model results. (ZIP 8235 kb) [file 12916_2016_665_MOESM3_ESM.zip › POLYMOD_GUI_6A_5.tif]

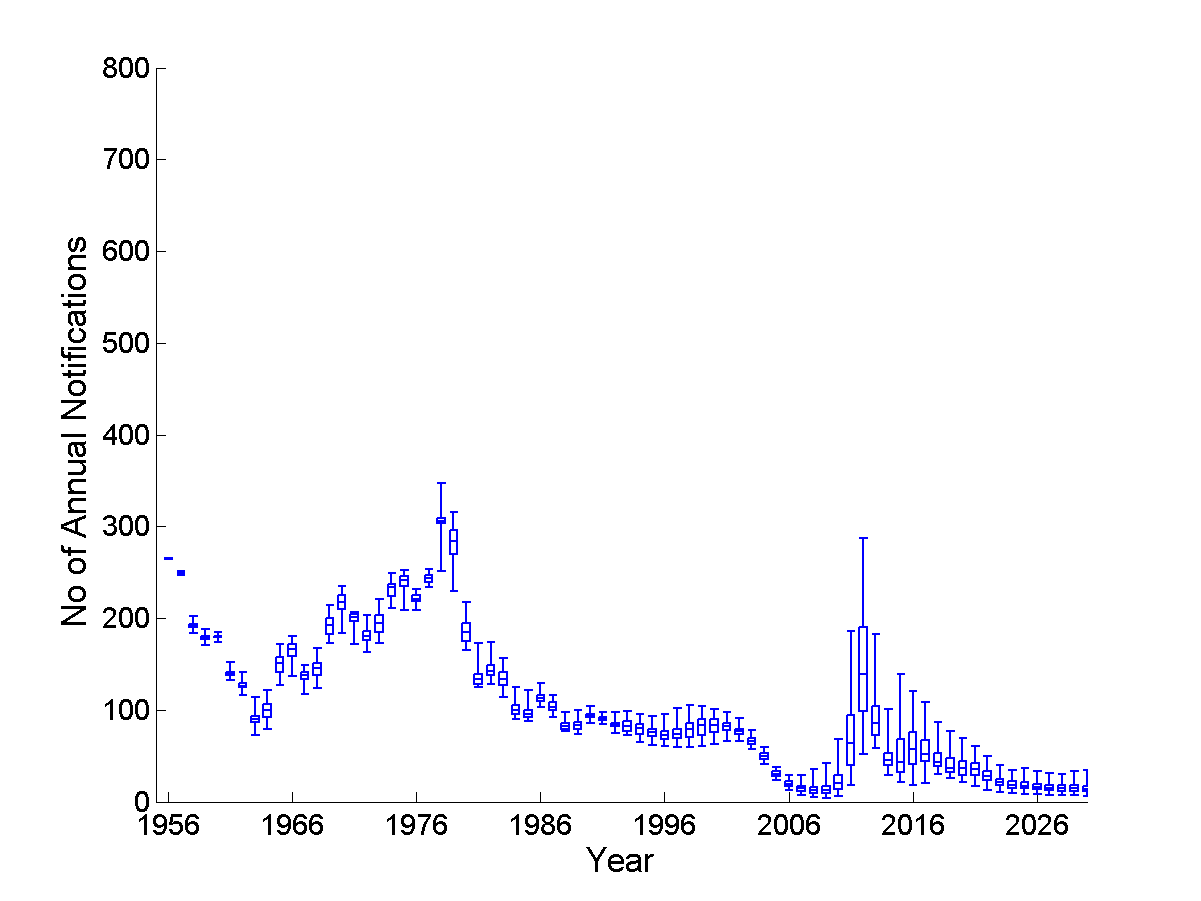

Supplement: Additional file 3: — Graphical User Interface porgramme to present pertussis simulation model results. (ZIP 8235 kb) [file 12916_2016_665_MOESM3_ESM.zip › POLYMOD_GUI_6A_6.tif]

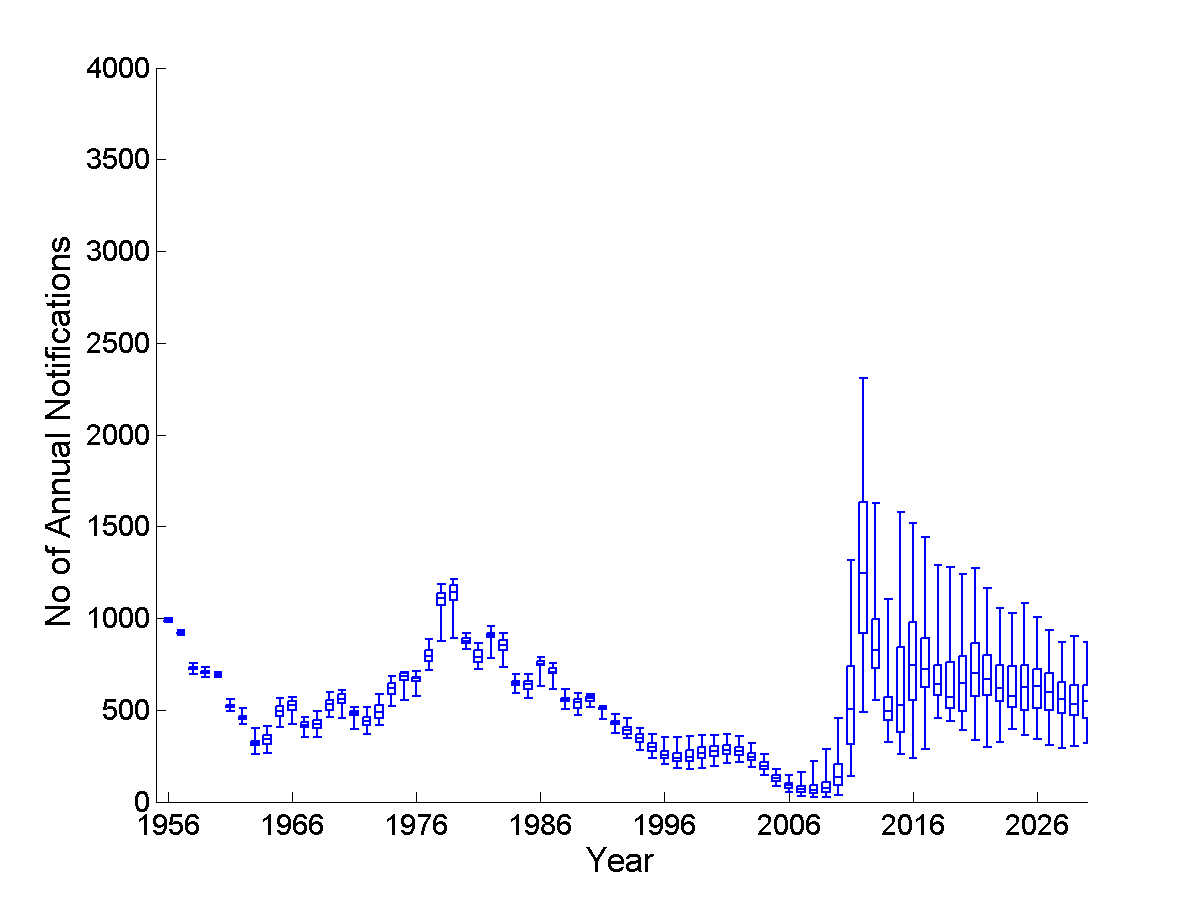

Supplement: Additional file 3: — Graphical User Interface porgramme to present pertussis simulation model results. (ZIP 8235 kb) [file 12916_2016_665_MOESM3_ESM.zip › POLYMOD_GUI_6A_7.tif]

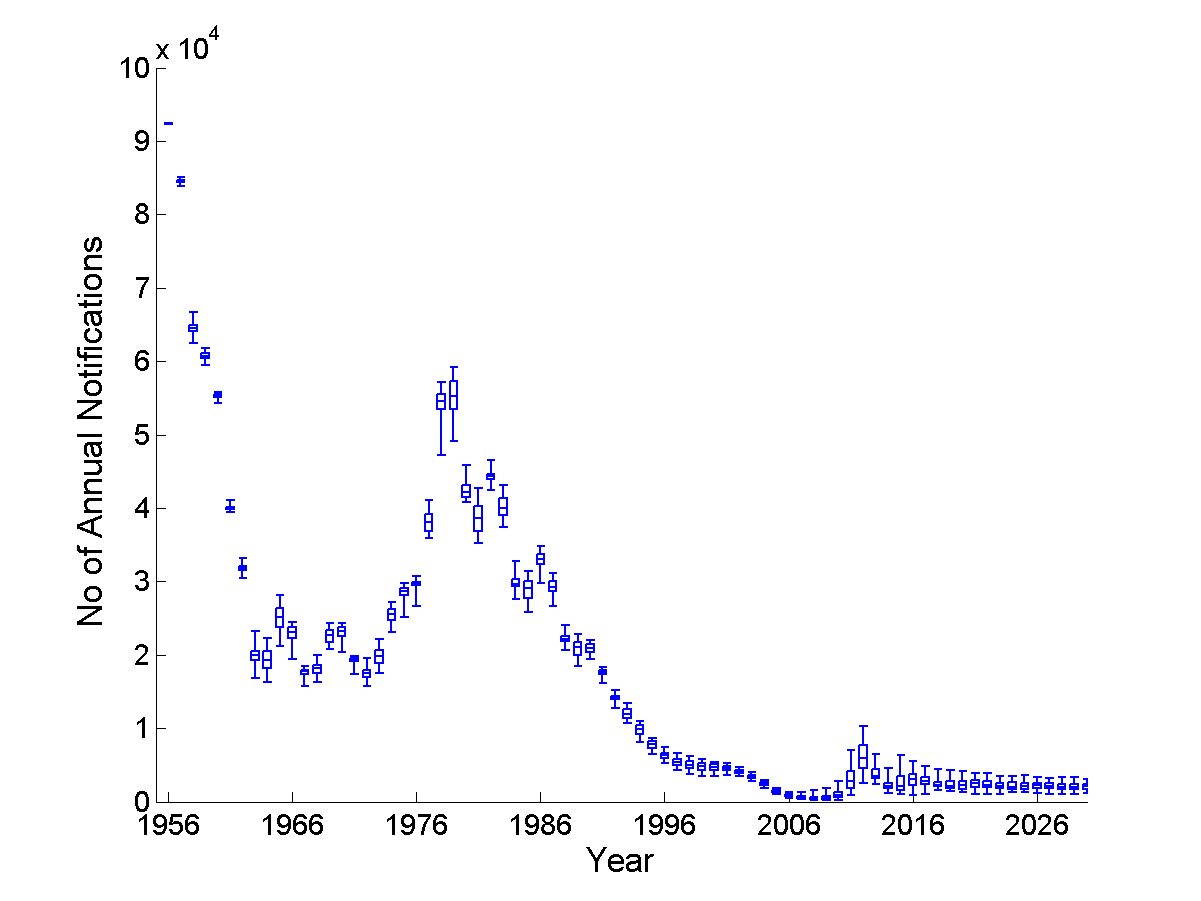

Supplement: Additional file 3: — Graphical User Interface porgramme to present pertussis simulation model results. (ZIP 8235 kb) [file 12916_2016_665_MOESM3_ESM.zip › POLYMOD_GUI_6A_8.tif]

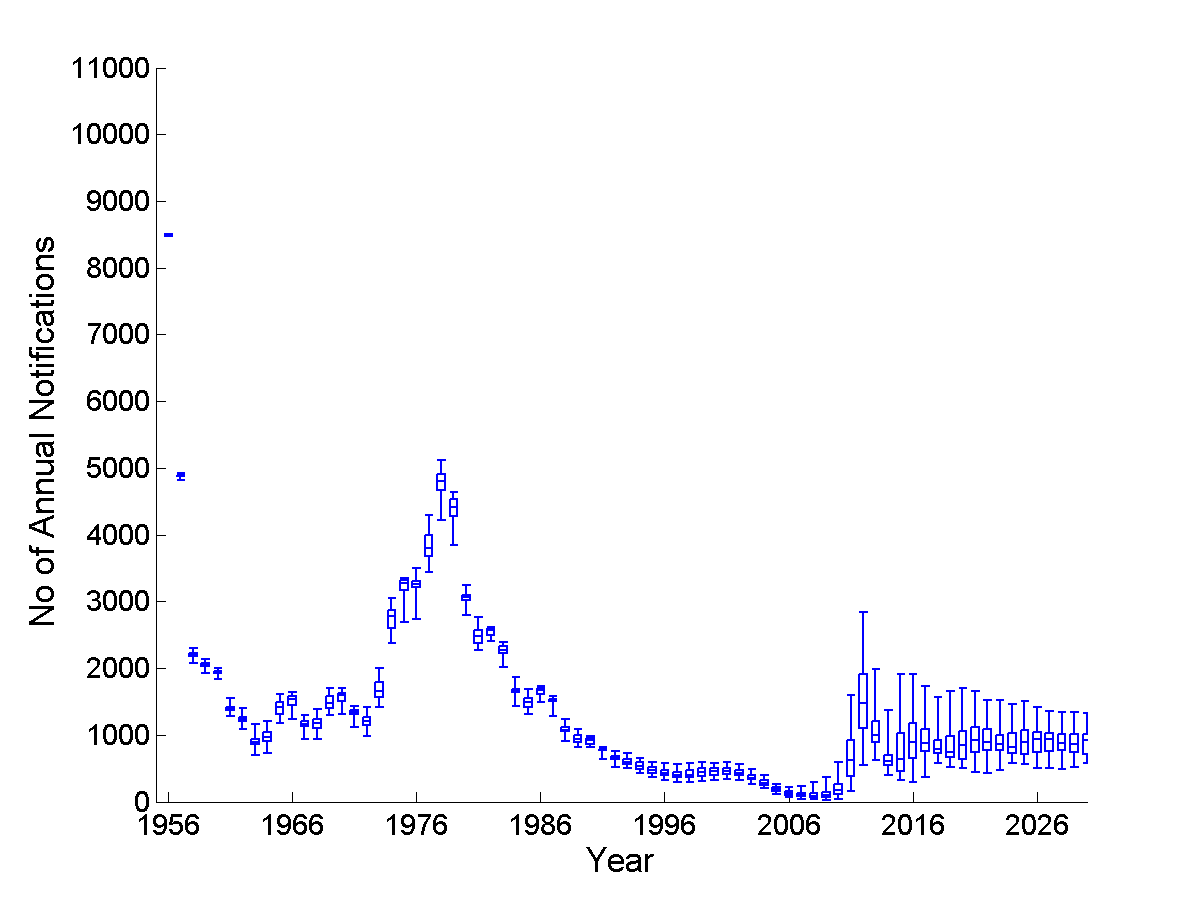

Supplement: Additional file 3: — Graphical User Interface porgramme to present pertussis simulation model results. (ZIP 8235 kb) [file 12916_2016_665_MOESM3_ESM.zip › POLYMOD_GUI_6B_1.tif]

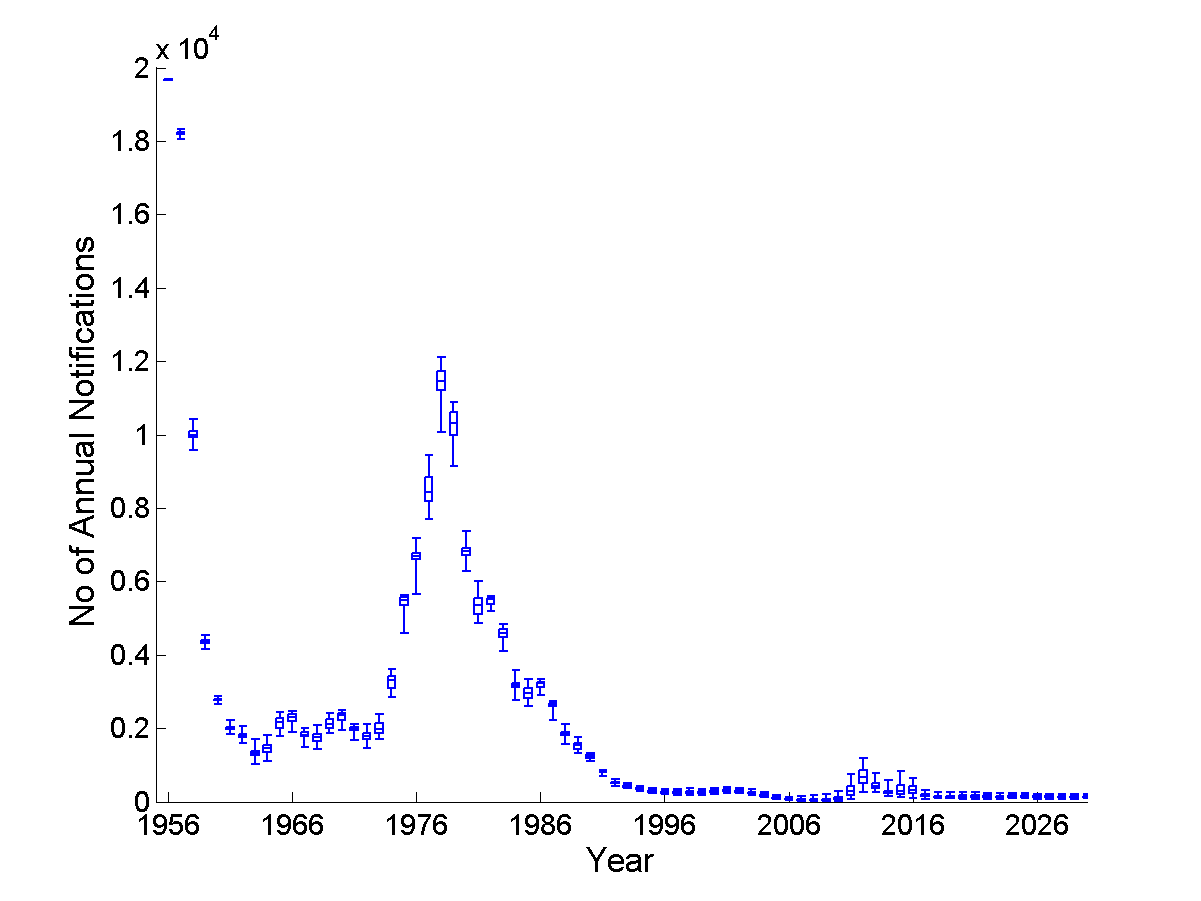

Supplement: Additional file 3: — Graphical User Interface porgramme to present pertussis simulation model results. (ZIP 8235 kb) [file 12916_2016_665_MOESM3_ESM.zip › POLYMOD_GUI_6B_2.tif]

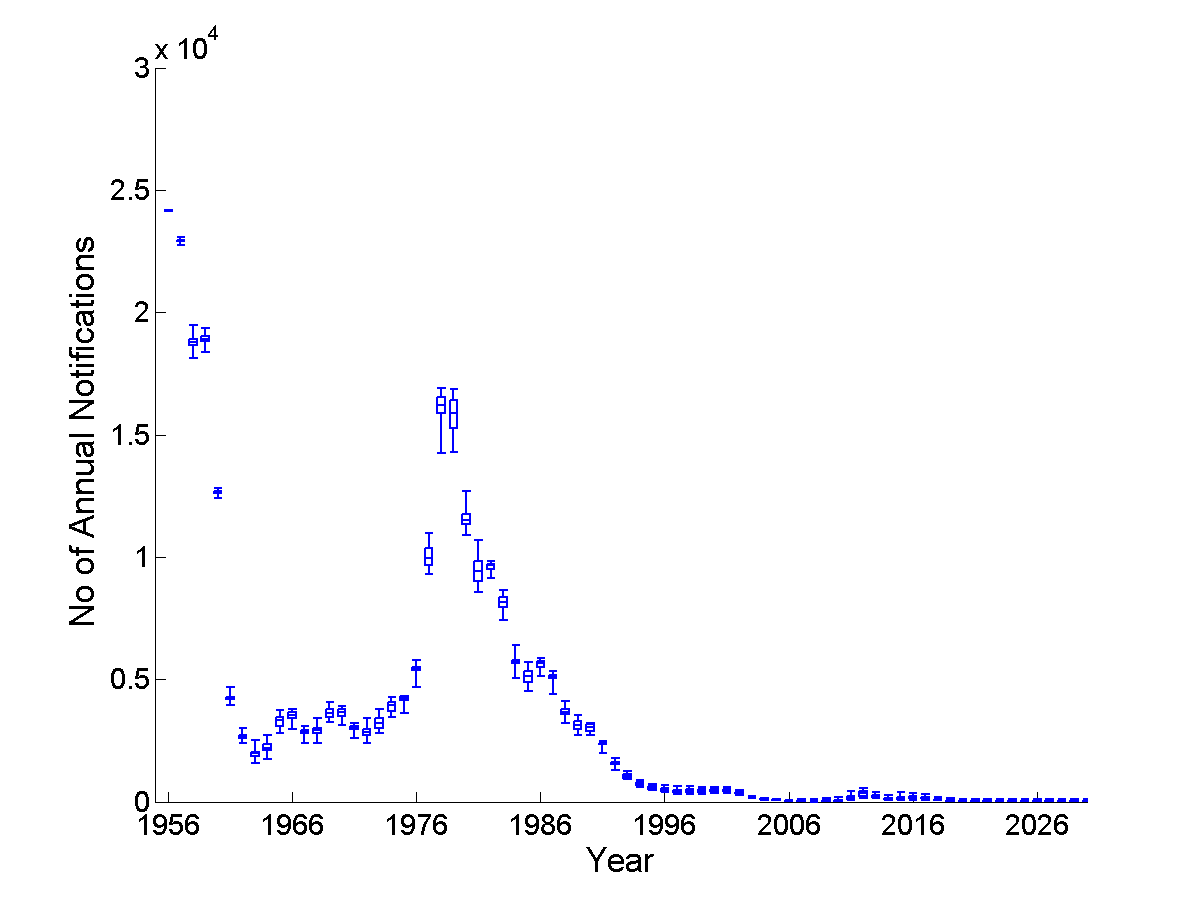

Supplement: Additional file 3: — Graphical User Interface porgramme to present pertussis simulation model results. (ZIP 8235 kb) [file 12916_2016_665_MOESM3_ESM.zip › POLYMOD_GUI_6B_3.tif]

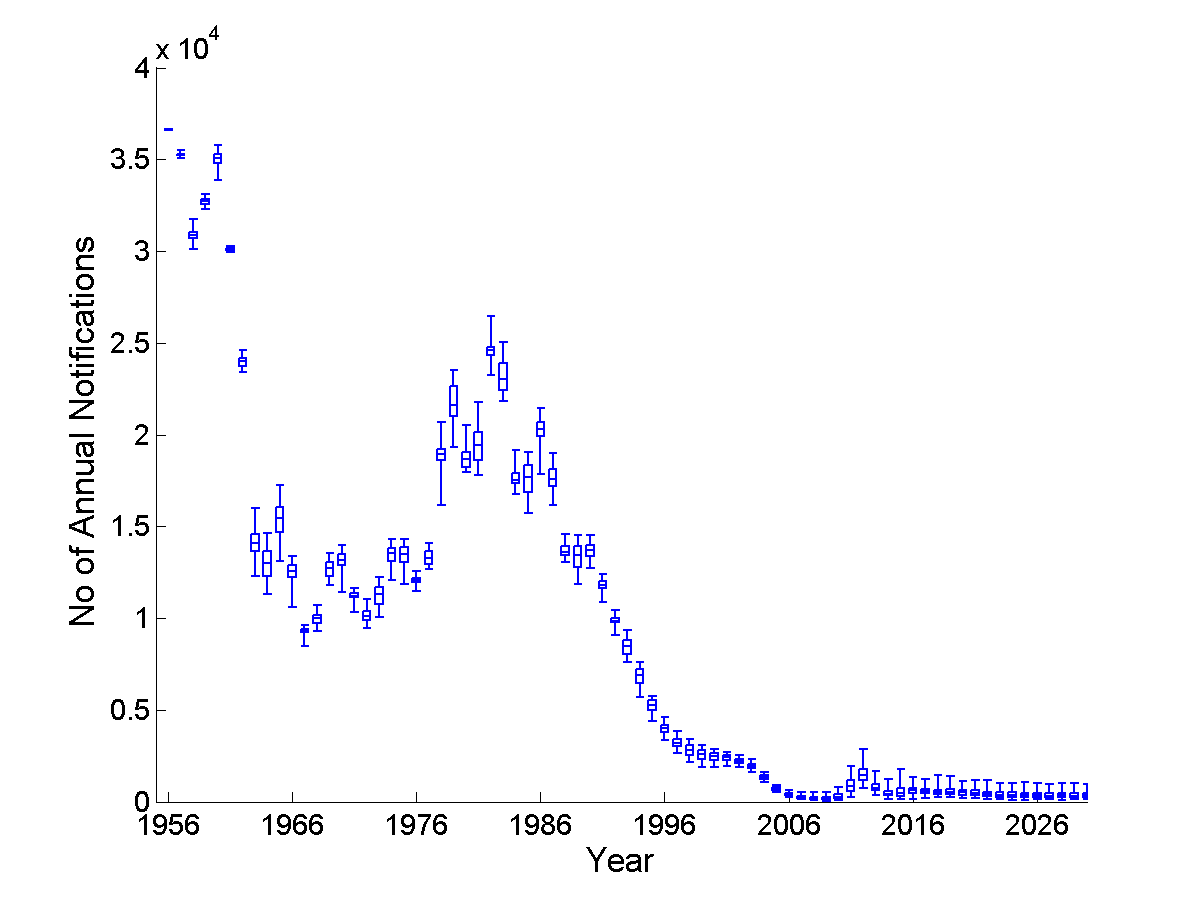

Supplement: Additional file 3: — Graphical User Interface porgramme to present pertussis simulation model results. (ZIP 8235 kb) [file 12916_2016_665_MOESM3_ESM.zip › POLYMOD_GUI_6B_4.tif]

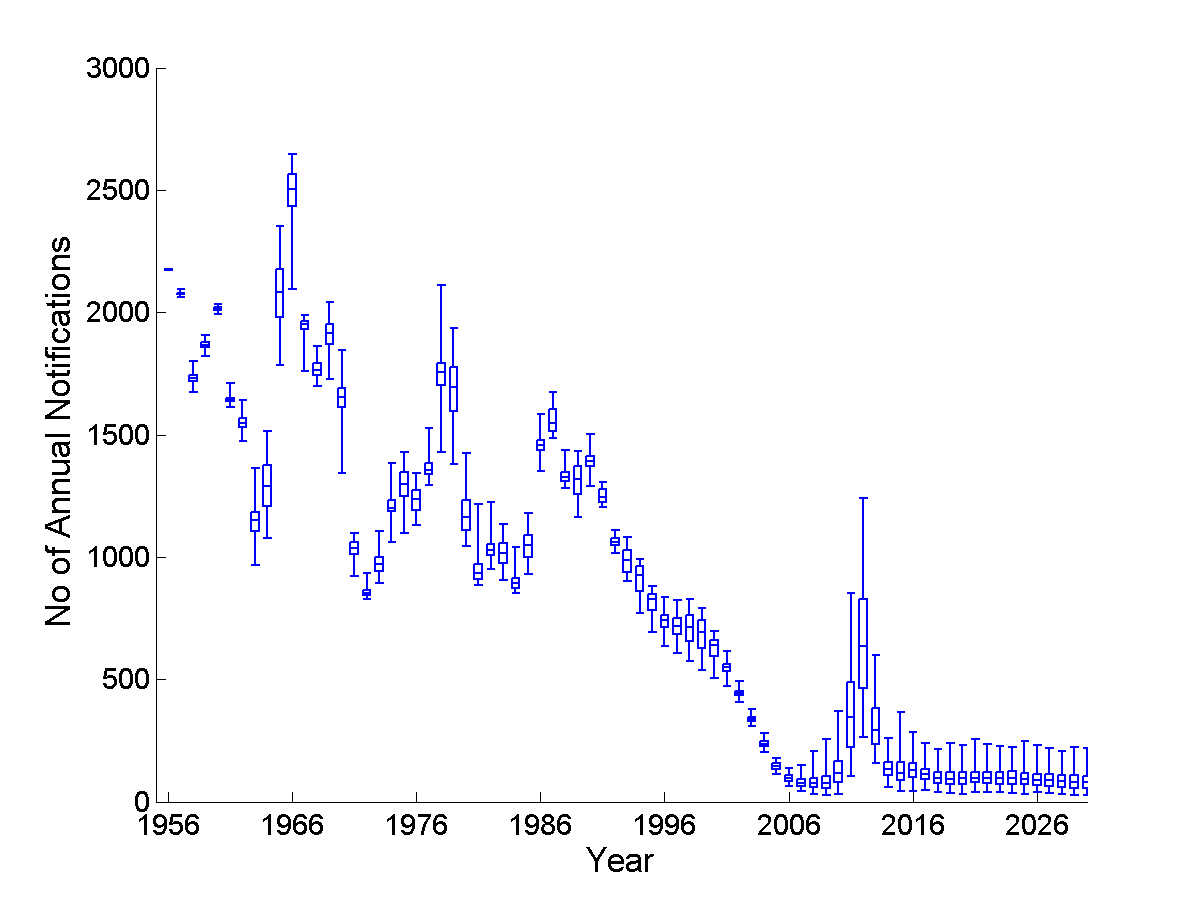

Supplement: Additional file 3: — Graphical User Interface porgramme to present pertussis simulation model results. (ZIP 8235 kb) [file 12916_2016_665_MOESM3_ESM.zip › POLYMOD_GUI_6B_5.tif]

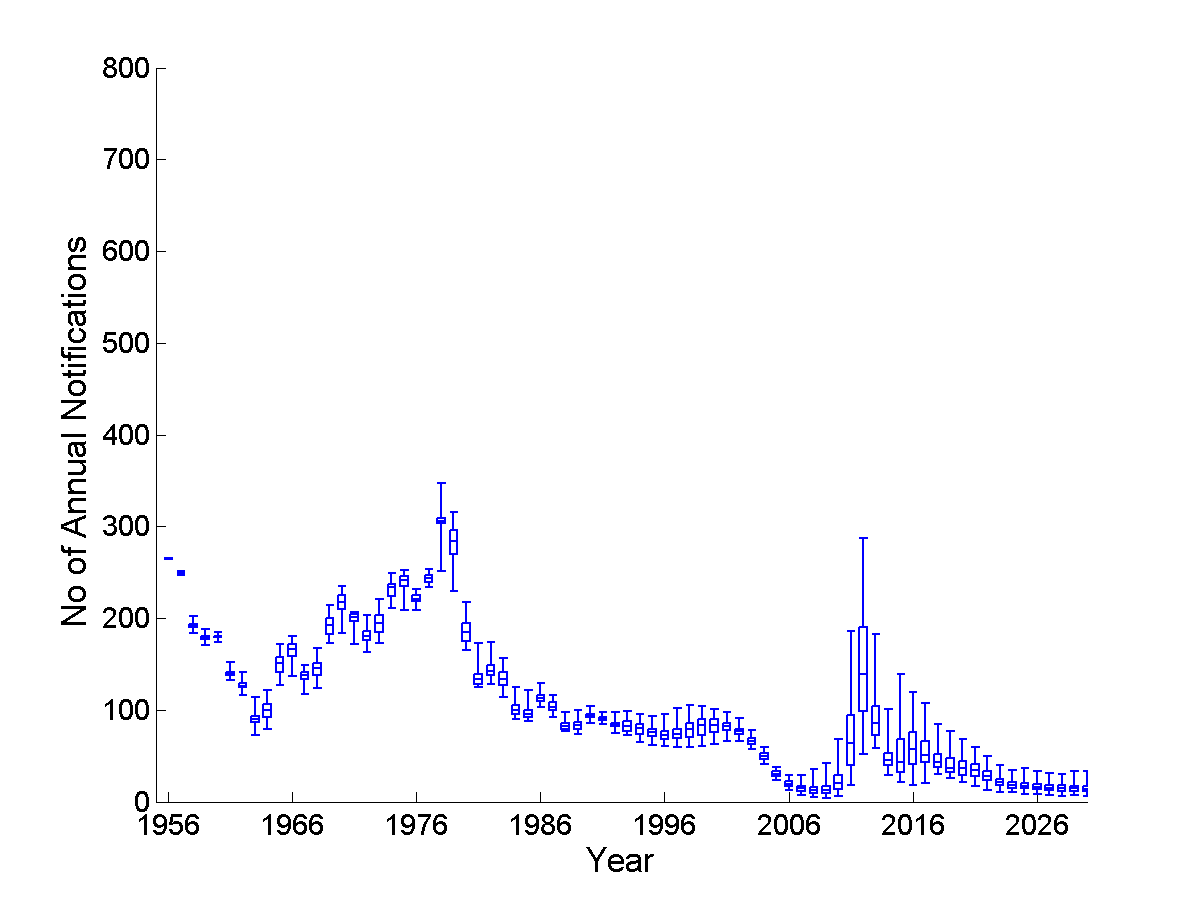

Supplement: Additional file 3: — Graphical User Interface porgramme to present pertussis simulation model results. (ZIP 8235 kb) [file 12916_2016_665_MOESM3_ESM.zip › POLYMOD_GUI_6B_6.tif]

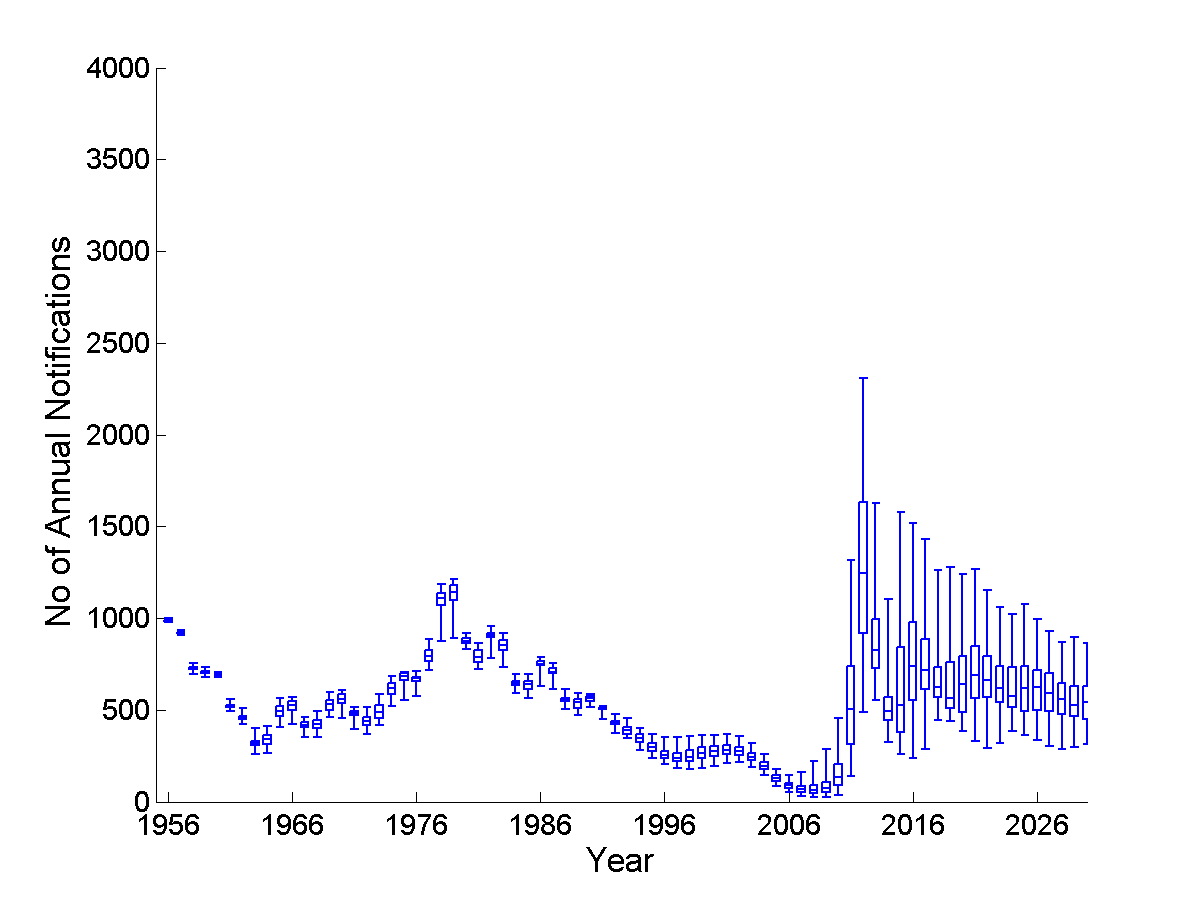

Supplement: Additional file 3: — Graphical User Interface porgramme to present pertussis simulation model results. (ZIP 8235 kb) [file 12916_2016_665_MOESM3_ESM.zip › POLYMOD_GUI_6B_7.tif]

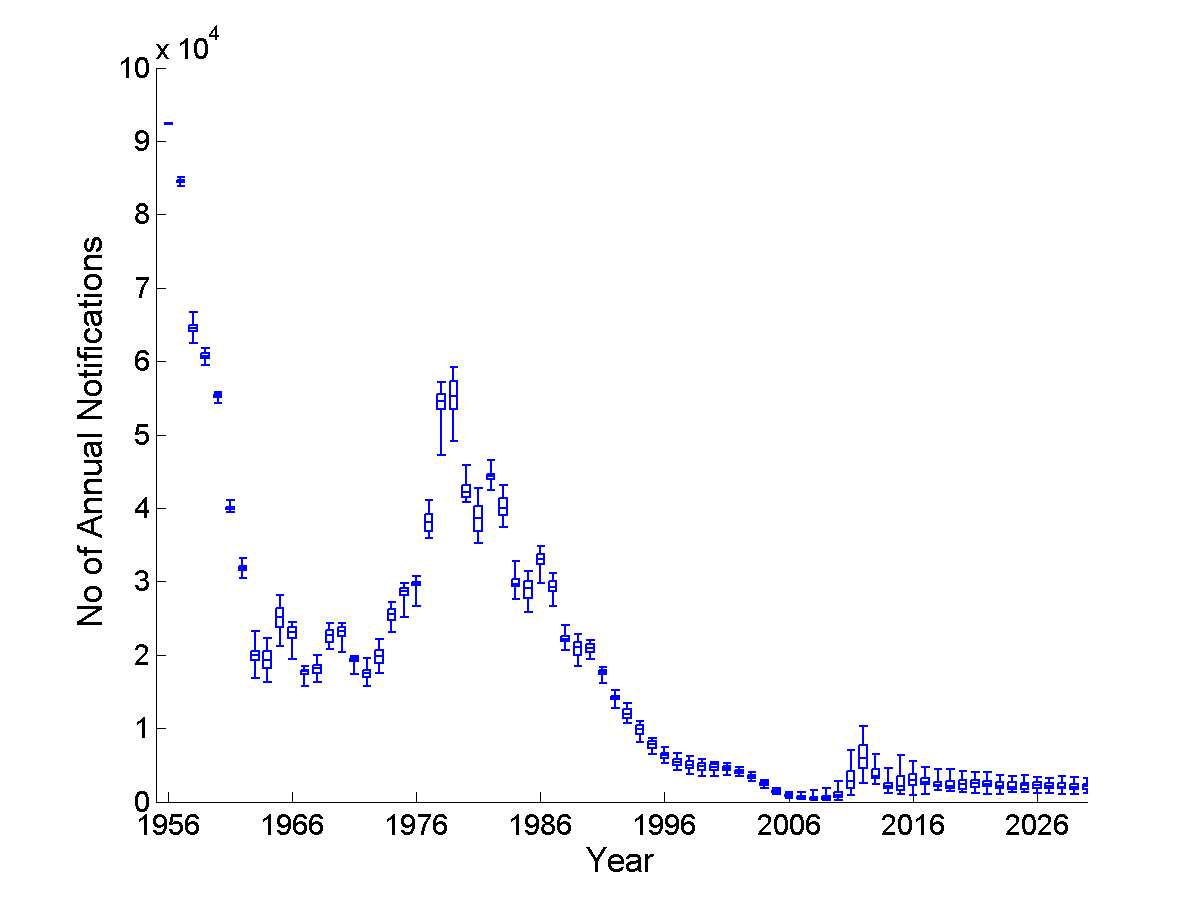

Supplement: Additional file 3: — Graphical User Interface porgramme to present pertussis simulation model results. (ZIP 8235 kb) [file 12916_2016_665_MOESM3_ESM.zip › POLYMOD_GUI_6B_8.tif]

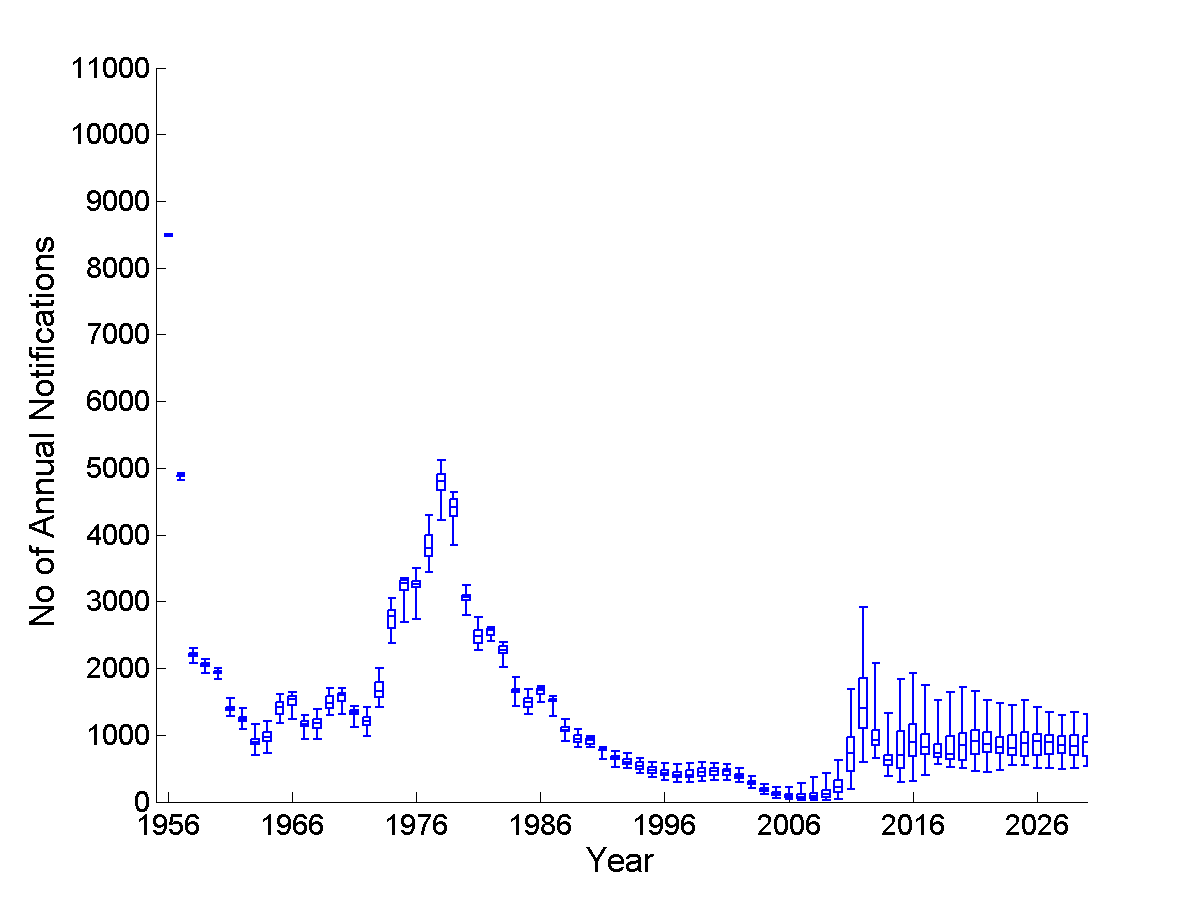

Supplement: Additional file 3: — Graphical User Interface porgramme to present pertussis simulation model results. (ZIP 8235 kb) [file 12916_2016_665_MOESM3_ESM.zip › POLYMOD_GUI_7_1.tif]

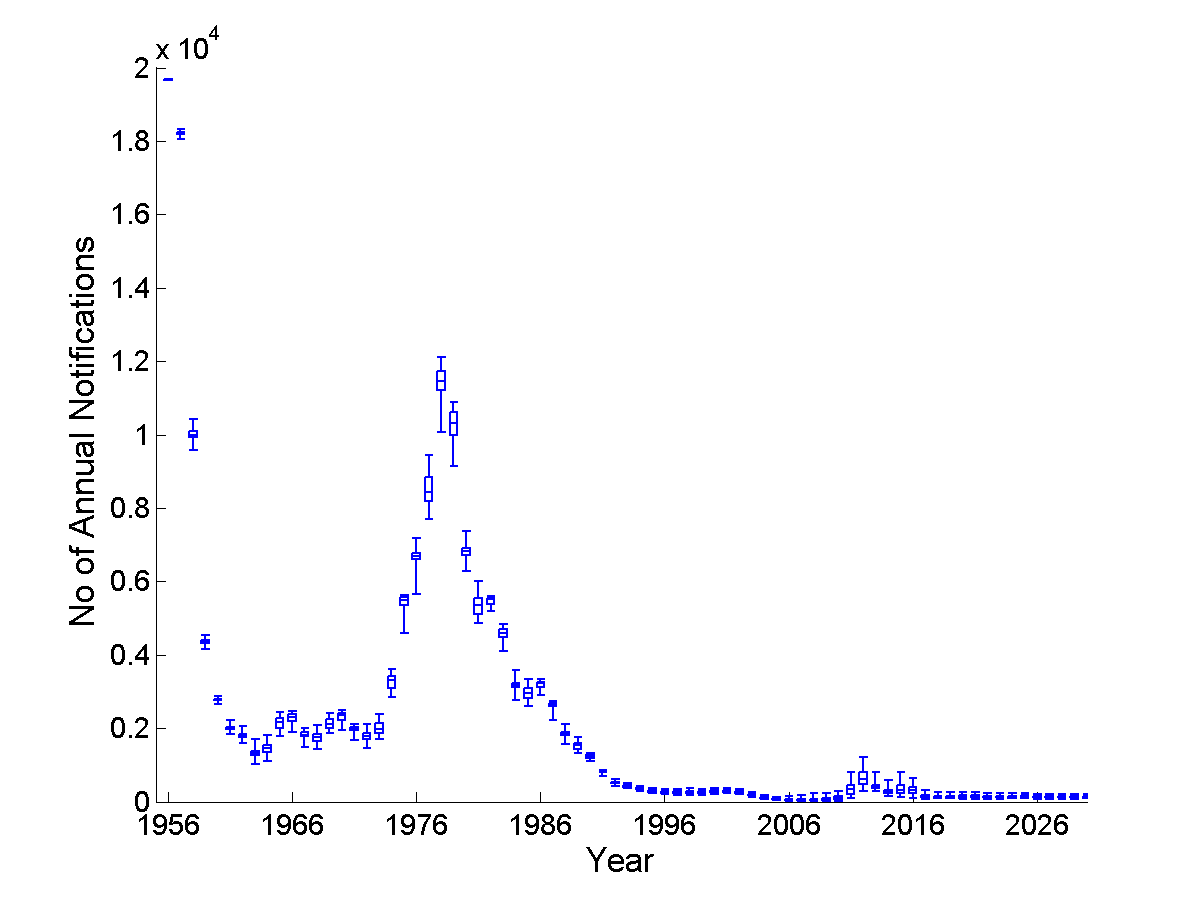

Supplement: Additional file 3: — Graphical User Interface porgramme to present pertussis simulation model results. (ZIP 8235 kb) [file 12916_2016_665_MOESM3_ESM.zip › POLYMOD_GUI_7_2.tif]

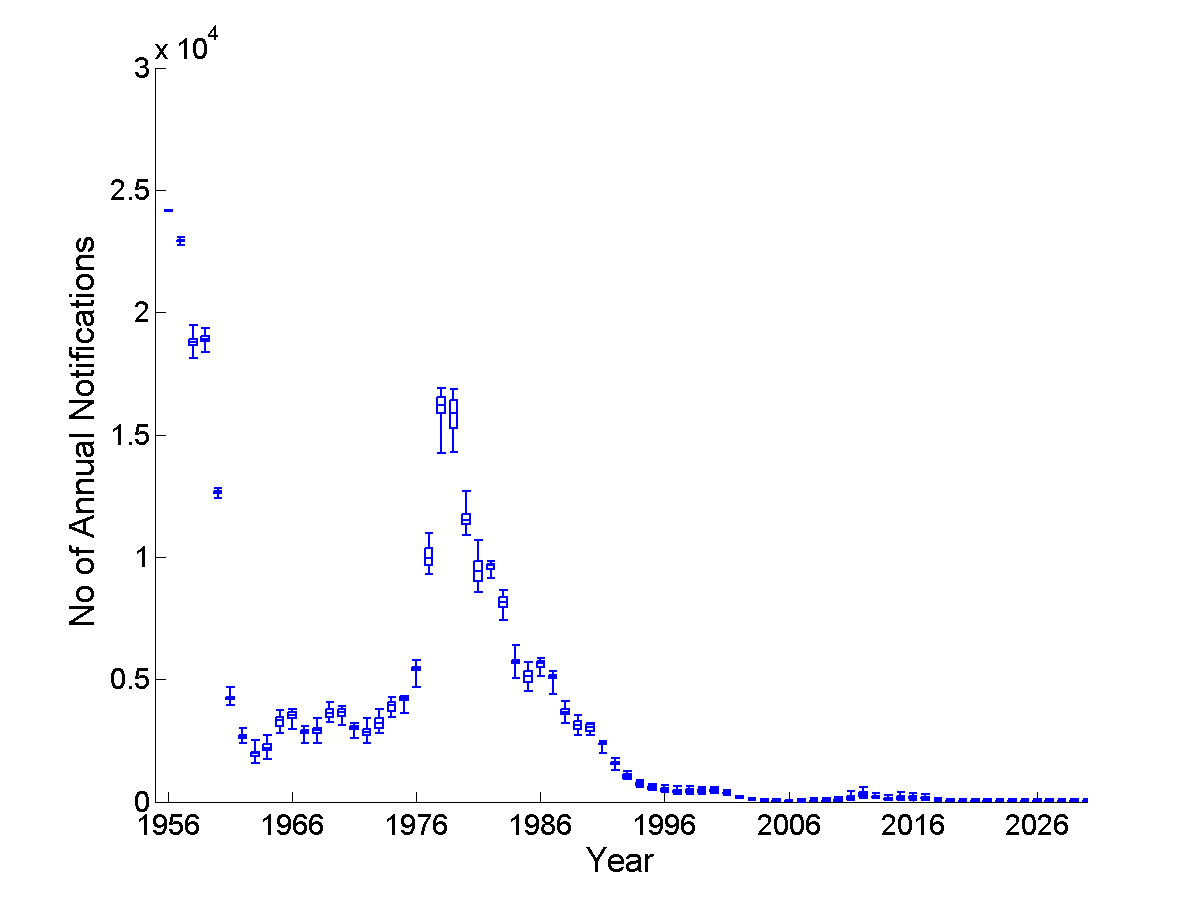

Supplement: Additional file 3: — Graphical User Interface porgramme to present pertussis simulation model results. (ZIP 8235 kb) [file 12916_2016_665_MOESM3_ESM.zip › POLYMOD_GUI_7_3.tif]

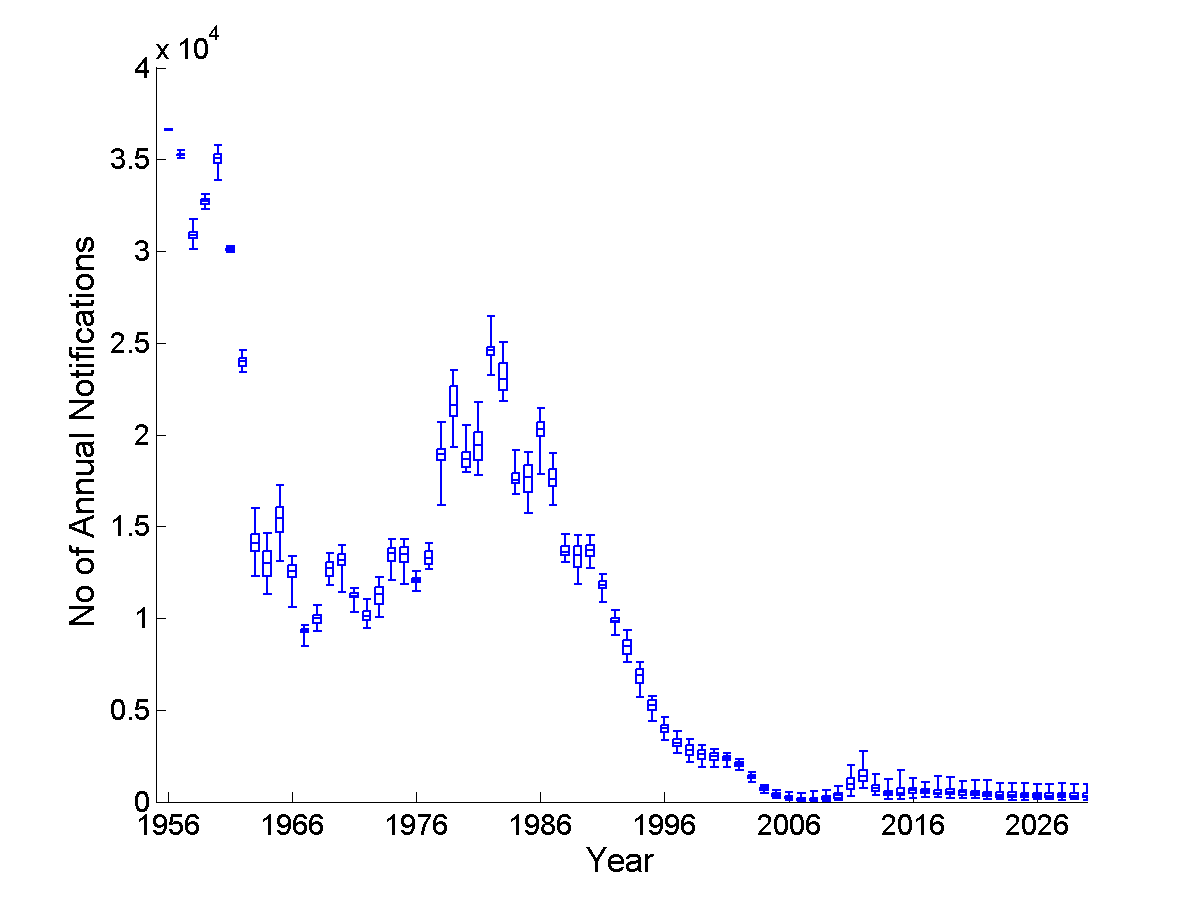

Supplement: Additional file 3: — Graphical User Interface porgramme to present pertussis simulation model results. (ZIP 8235 kb) [file 12916_2016_665_MOESM3_ESM.zip › POLYMOD_GUI_7_4.tif]

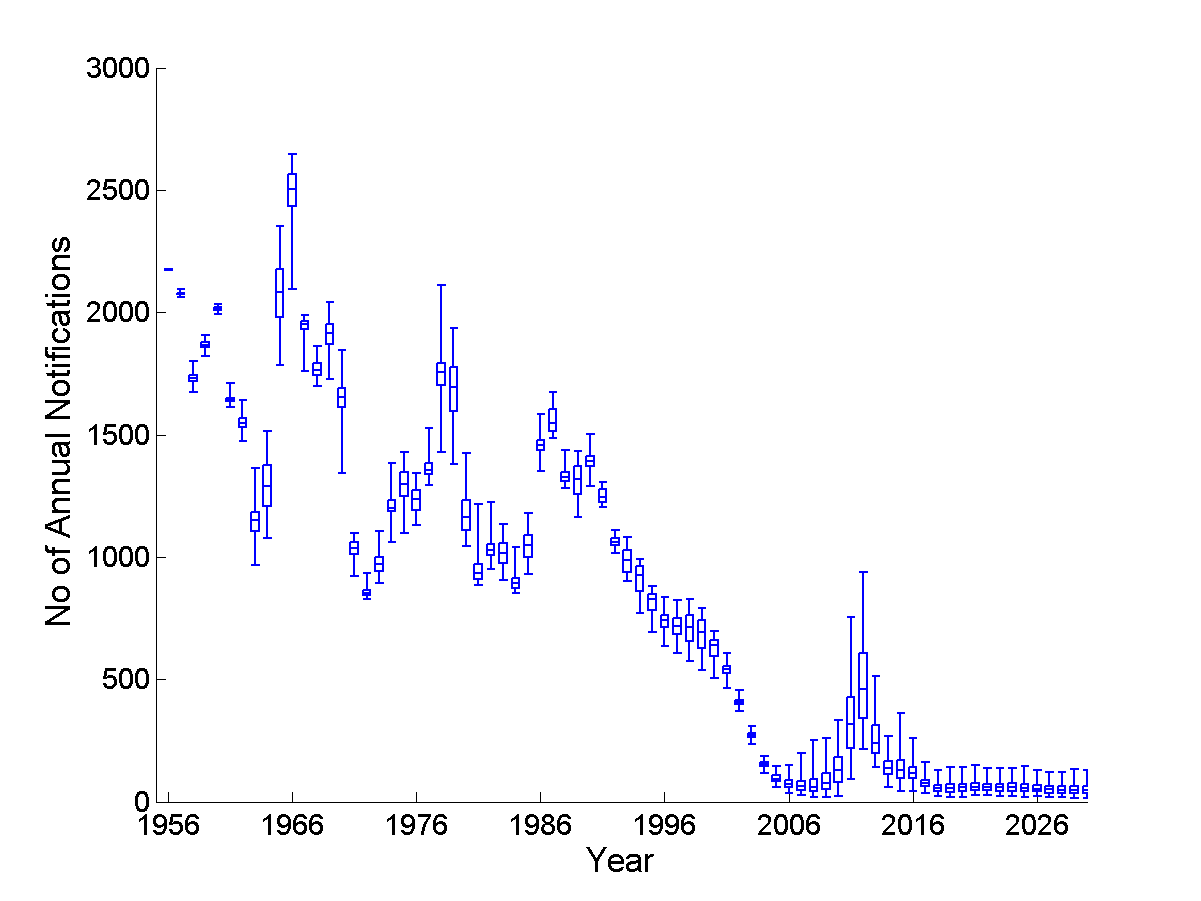

Supplement: Additional file 3: — Graphical User Interface porgramme to present pertussis simulation model results. (ZIP 8235 kb) [file 12916_2016_665_MOESM3_ESM.zip › POLYMOD_GUI_7_5.tif]

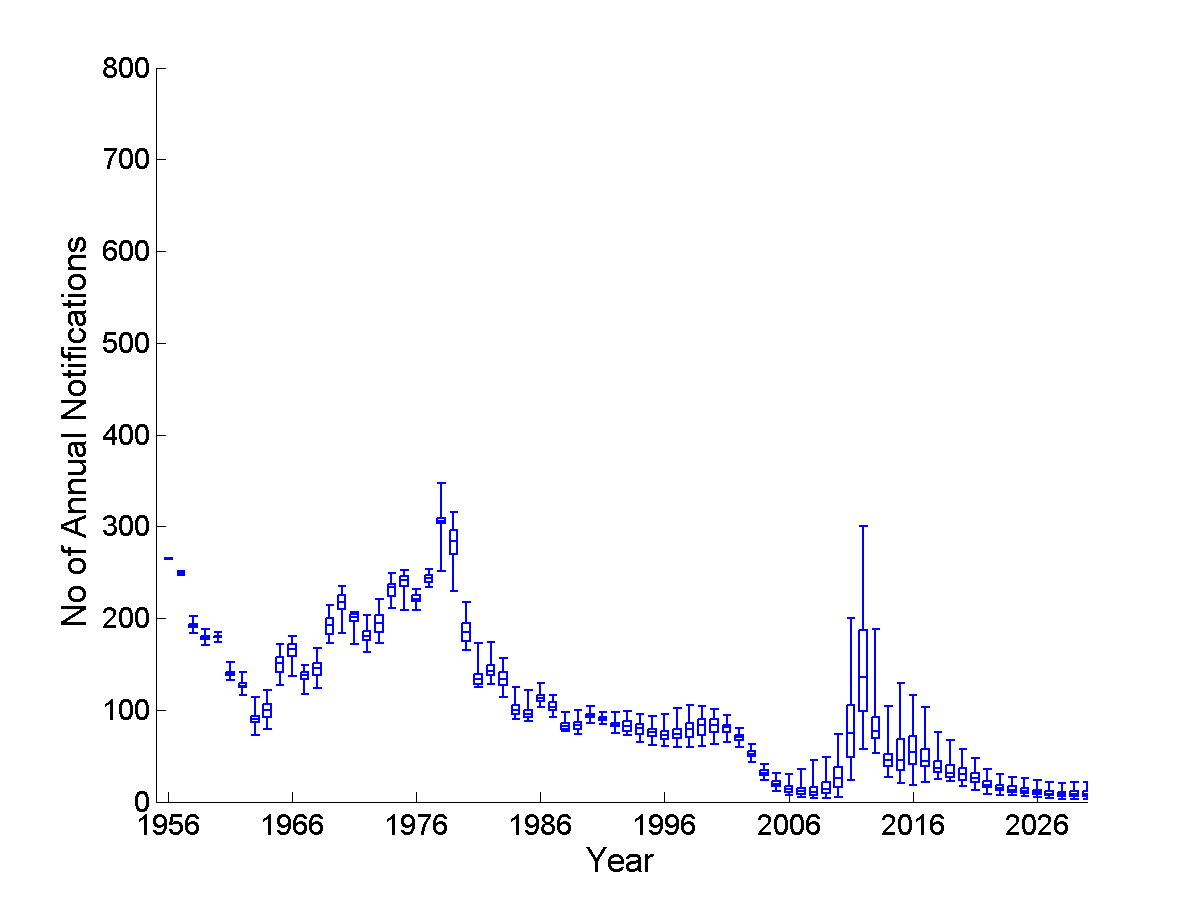

Supplement: Additional file 3: — Graphical User Interface porgramme to present pertussis simulation model results. (ZIP 8235 kb) [file 12916_2016_665_MOESM3_ESM.zip › POLYMOD_GUI_7_6.tif]

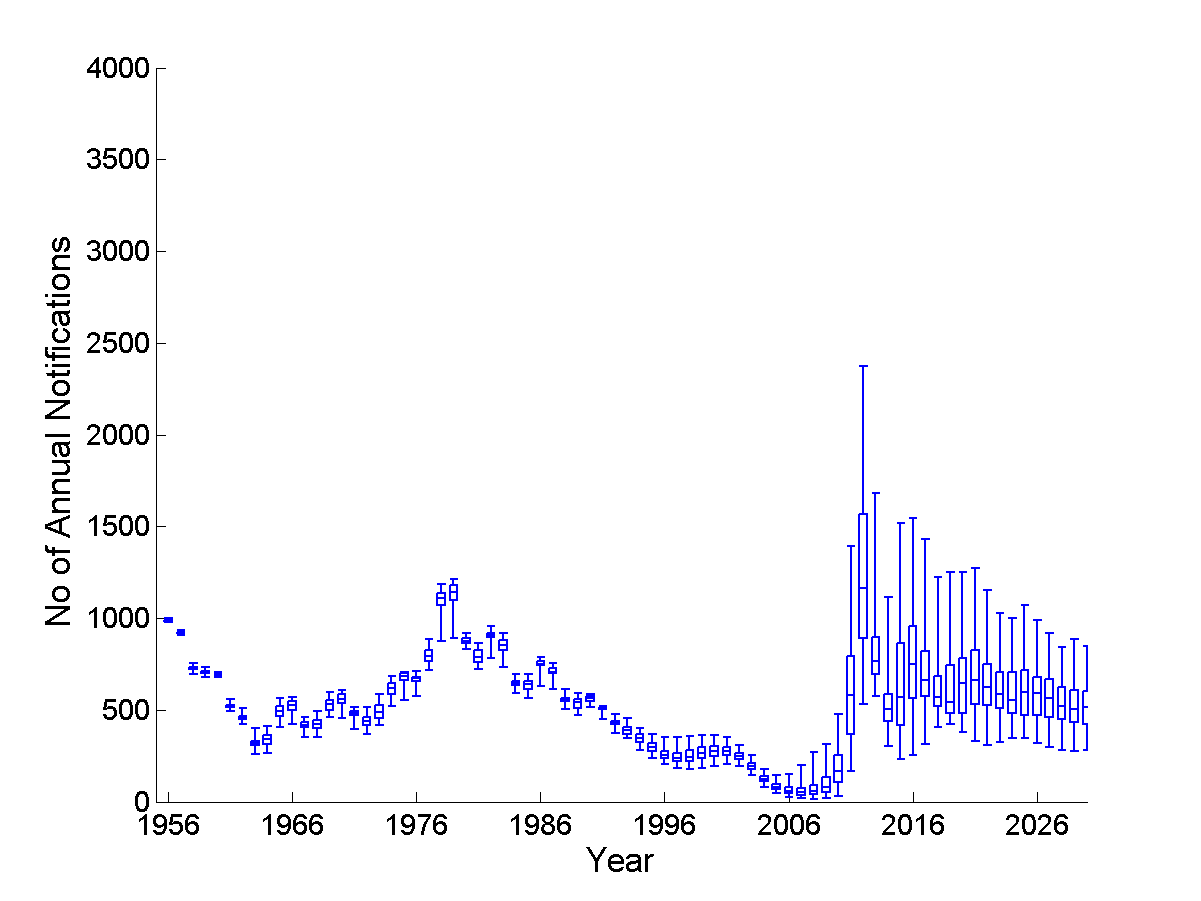

Supplement: Additional file 3: — Graphical User Interface porgramme to present pertussis simulation model results. (ZIP 8235 kb) [file 12916_2016_665_MOESM3_ESM.zip › POLYMOD_GUI_7_7.tif]

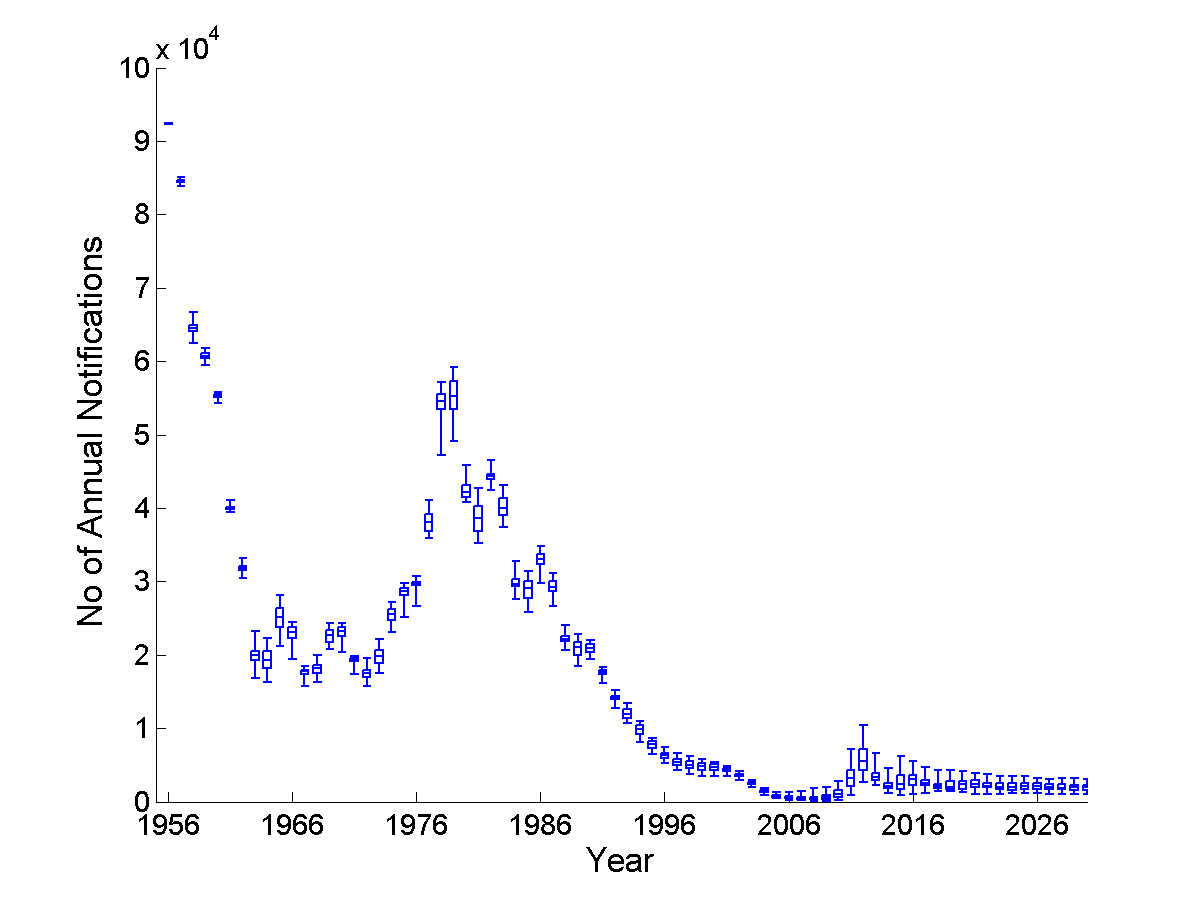

Supplement: Additional file 3: — Graphical User Interface porgramme to present pertussis simulation model results. (ZIP 8235 kb) [file 12916_2016_665_MOESM3_ESM.zip › POLYMOD_GUI_7_8.tif]

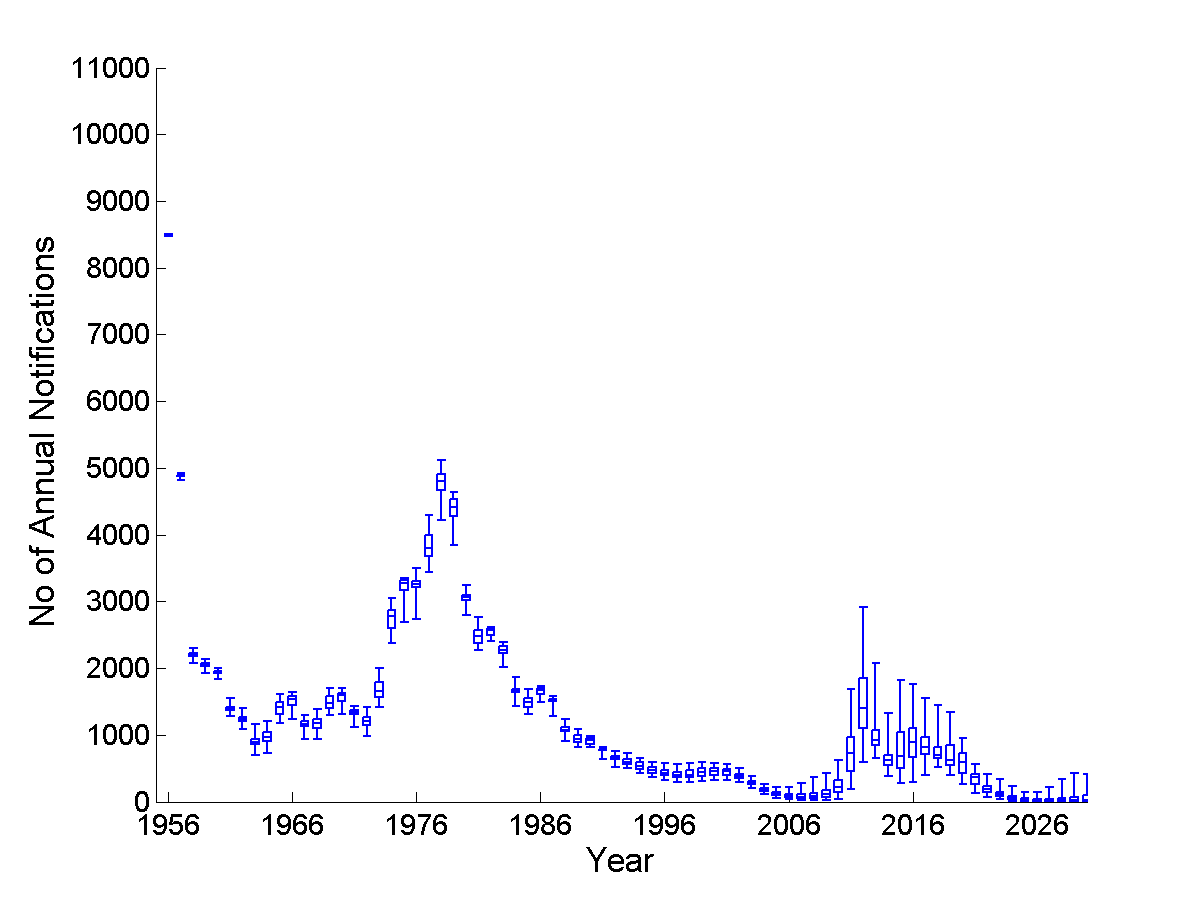

Supplement: Additional file 3: — Graphical User Interface porgramme to present pertussis simulation model results. (ZIP 8235 kb) [file 12916_2016_665_MOESM3_ESM.zip › POLYMOD_GUI_8_1.tif]

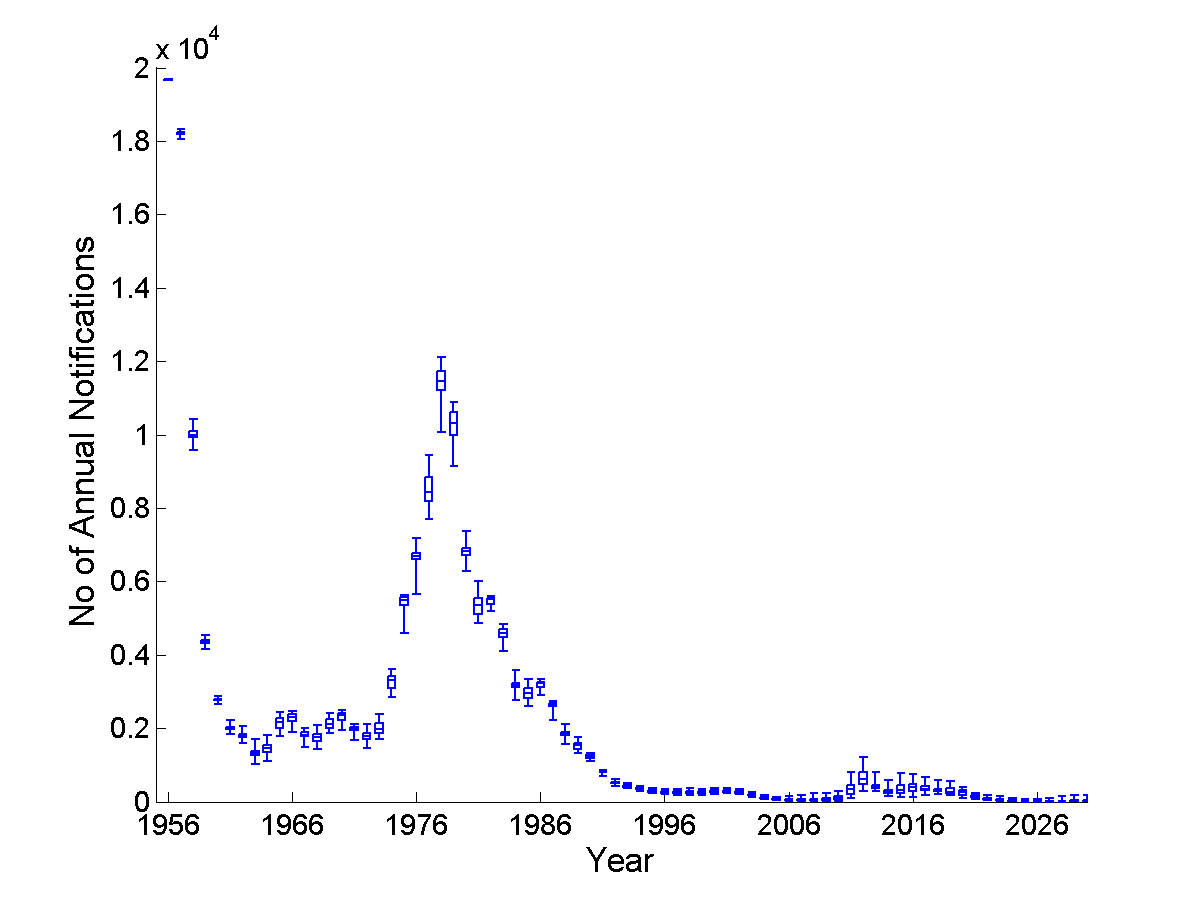

Supplement: Additional file 3: — Graphical User Interface porgramme to present pertussis simulation model results. (ZIP 8235 kb) [file 12916_2016_665_MOESM3_ESM.zip › POLYMOD_GUI_8_2.tif]

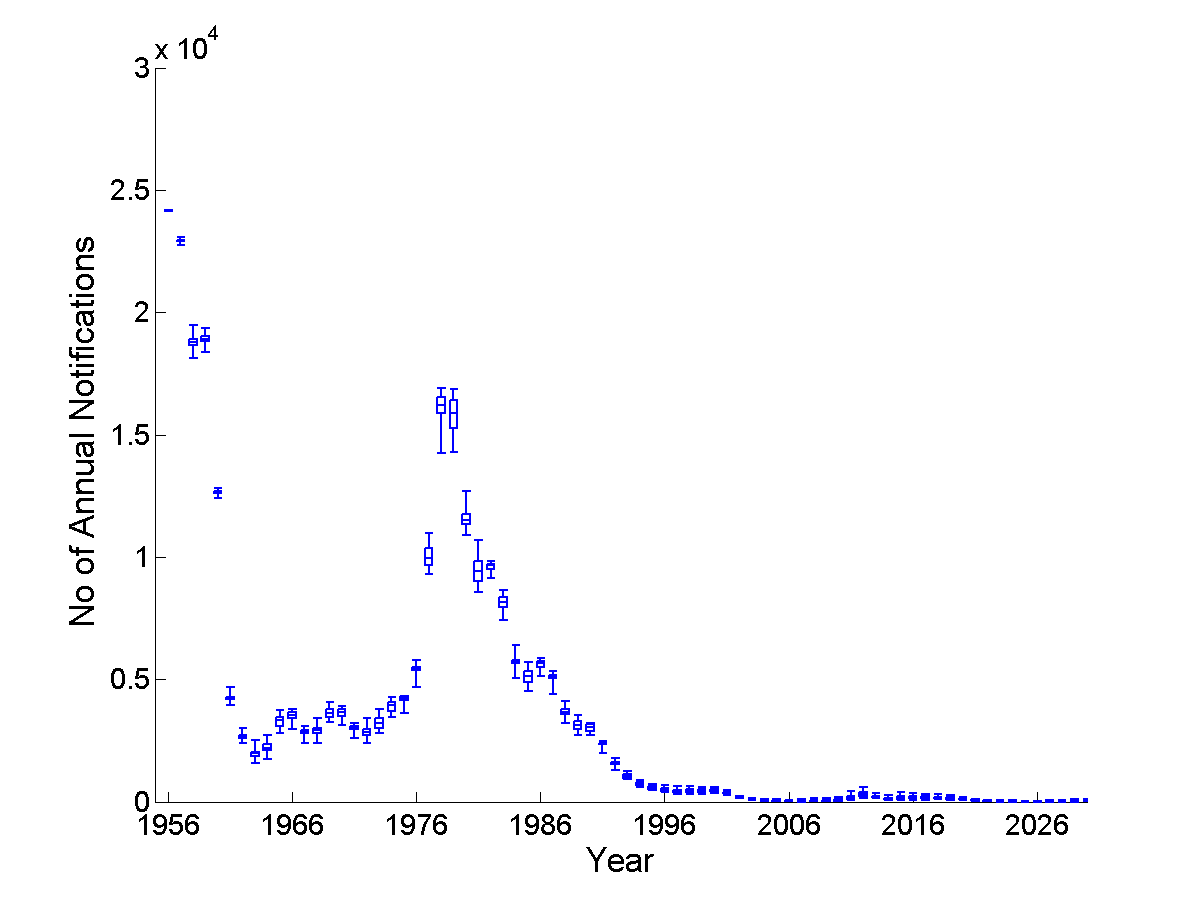

Supplement: Additional file 3: — Graphical User Interface porgramme to present pertussis simulation model results. (ZIP 8235 kb) [file 12916_2016_665_MOESM3_ESM.zip › POLYMOD_GUI_8_3.tif]

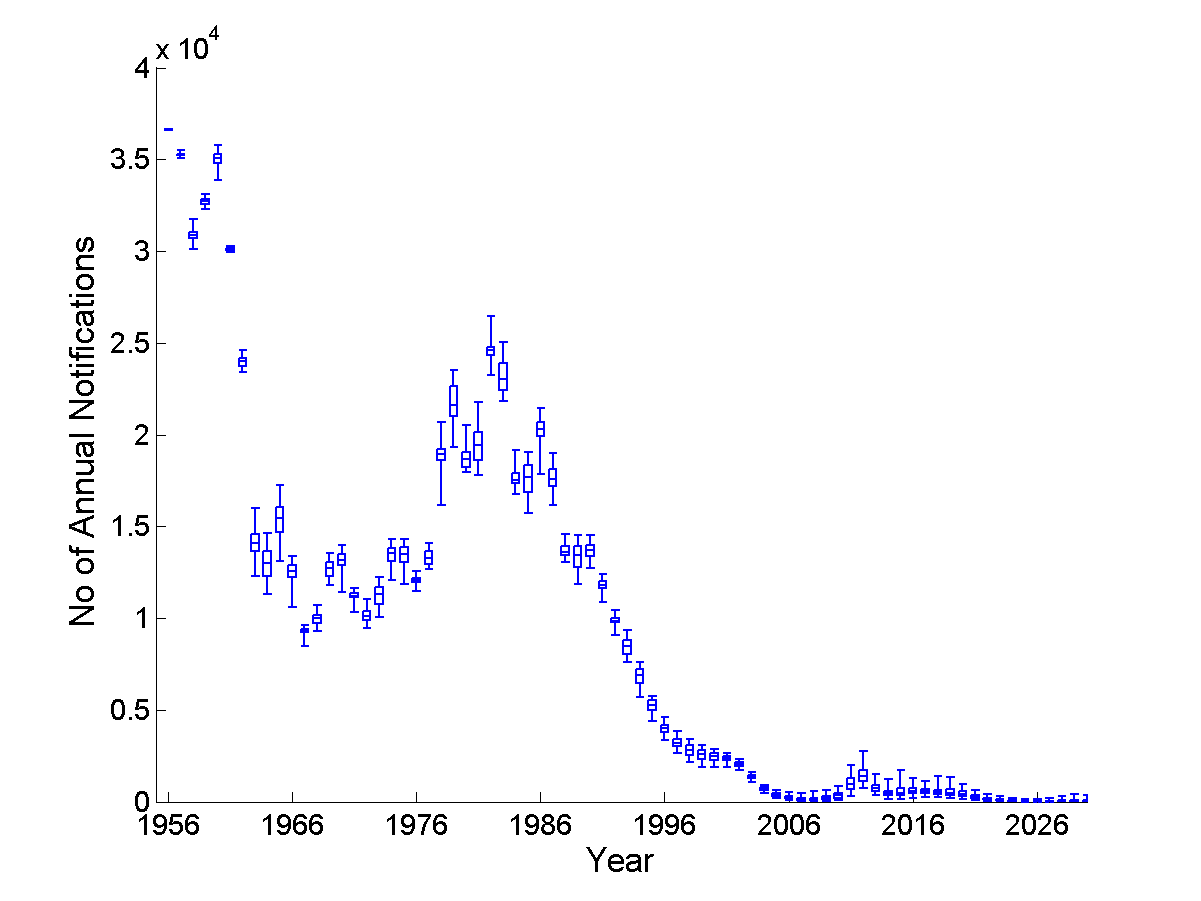

Supplement: Additional file 3: — Graphical User Interface porgramme to present pertussis simulation model results. (ZIP 8235 kb) [file 12916_2016_665_MOESM3_ESM.zip › POLYMOD_GUI_8_4.tif]

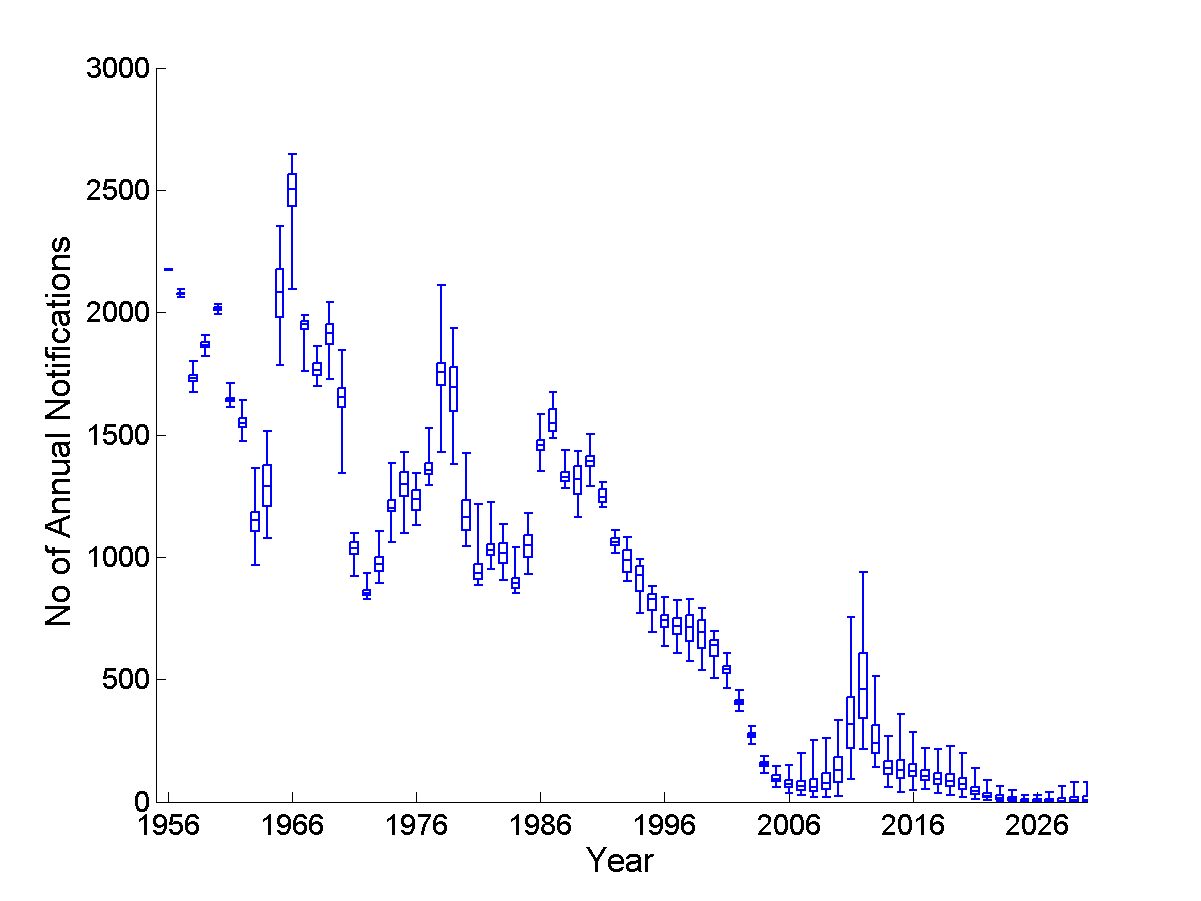

Supplement: Additional file 3: — Graphical User Interface porgramme to present pertussis simulation model results. (ZIP 8235 kb) [file 12916_2016_665_MOESM3_ESM.zip › POLYMOD_GUI_8_5.tif]

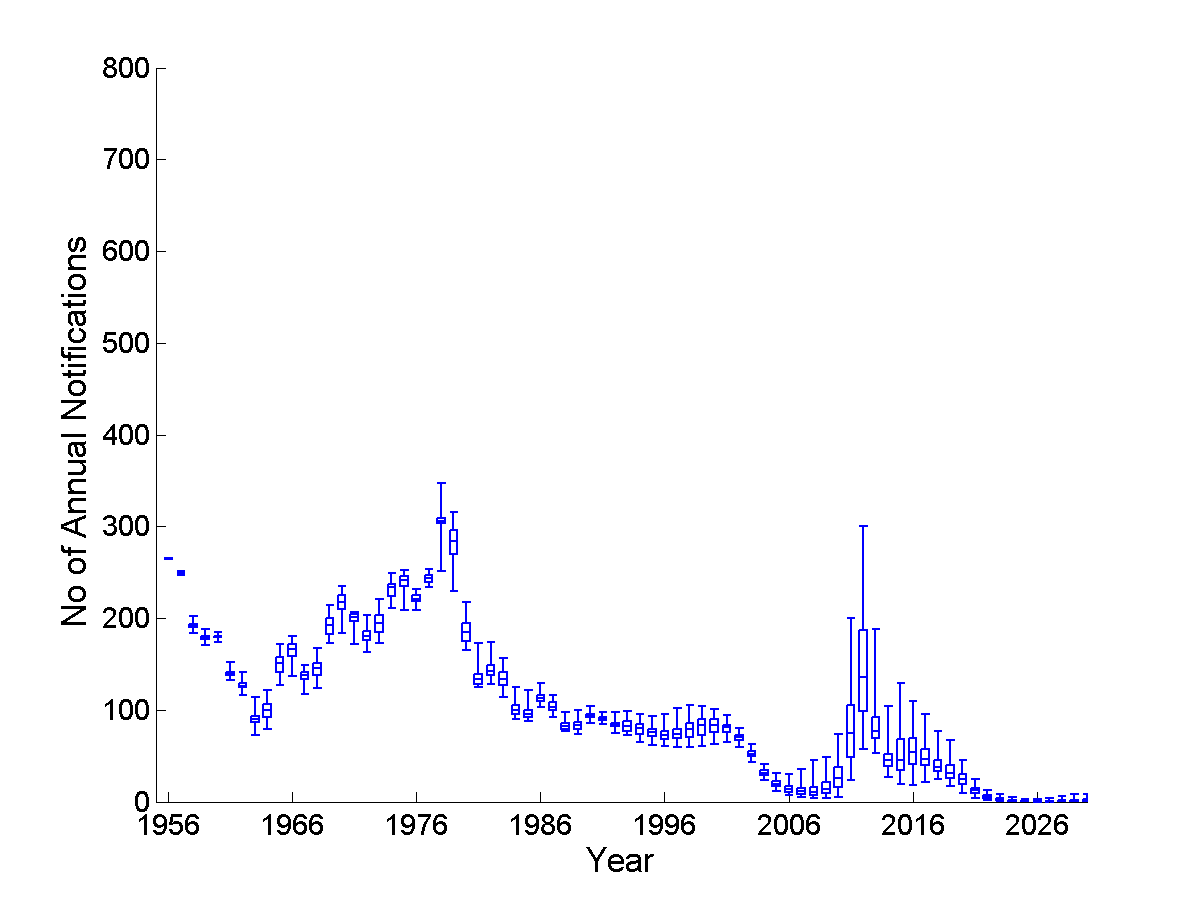

Supplement: Additional file 3: — Graphical User Interface porgramme to present pertussis simulation model results. (ZIP 8235 kb) [file 12916_2016_665_MOESM3_ESM.zip › POLYMOD_GUI_8_6.tif]

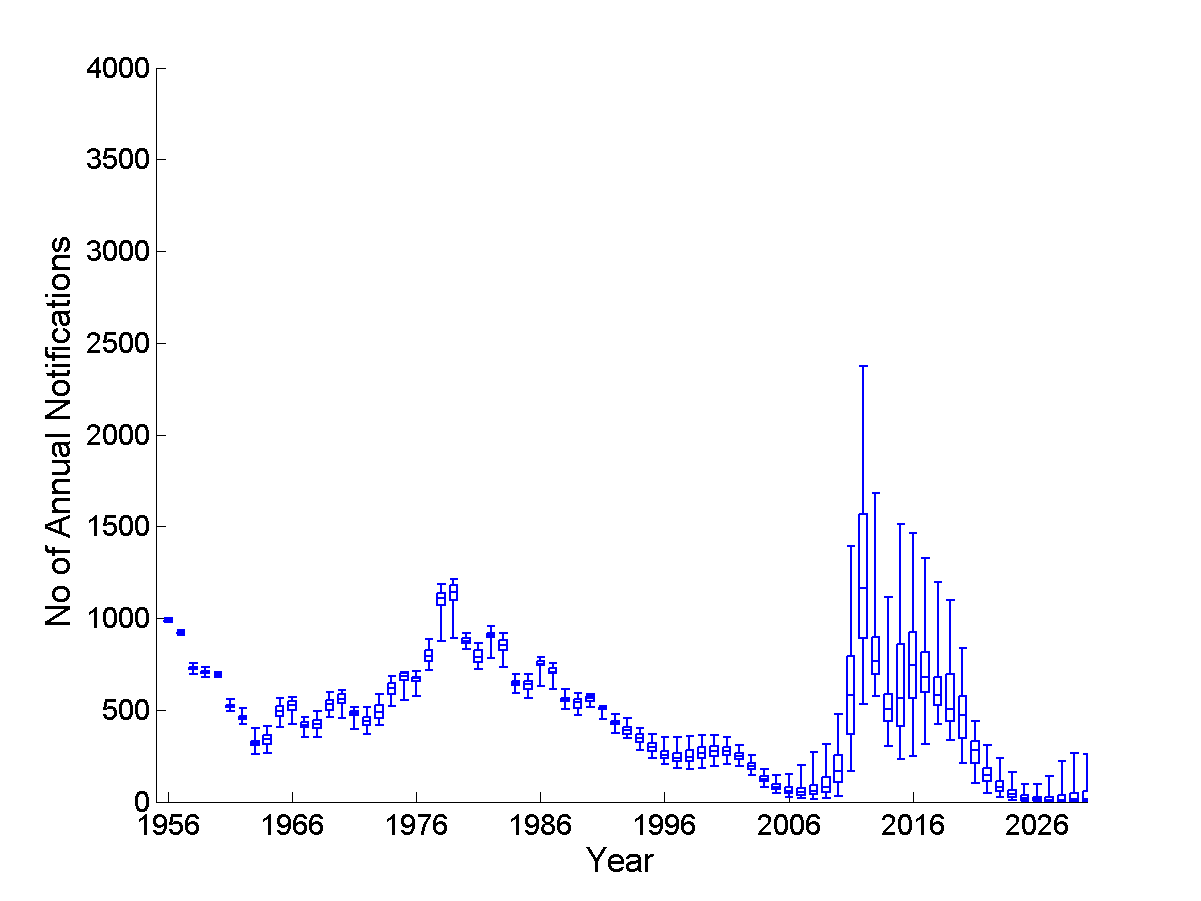

Supplement: Additional file 3: — Graphical User Interface porgramme to present pertussis simulation model results. (ZIP 8235 kb) [file 12916_2016_665_MOESM3_ESM.zip › POLYMOD_GUI_8_7.tif]

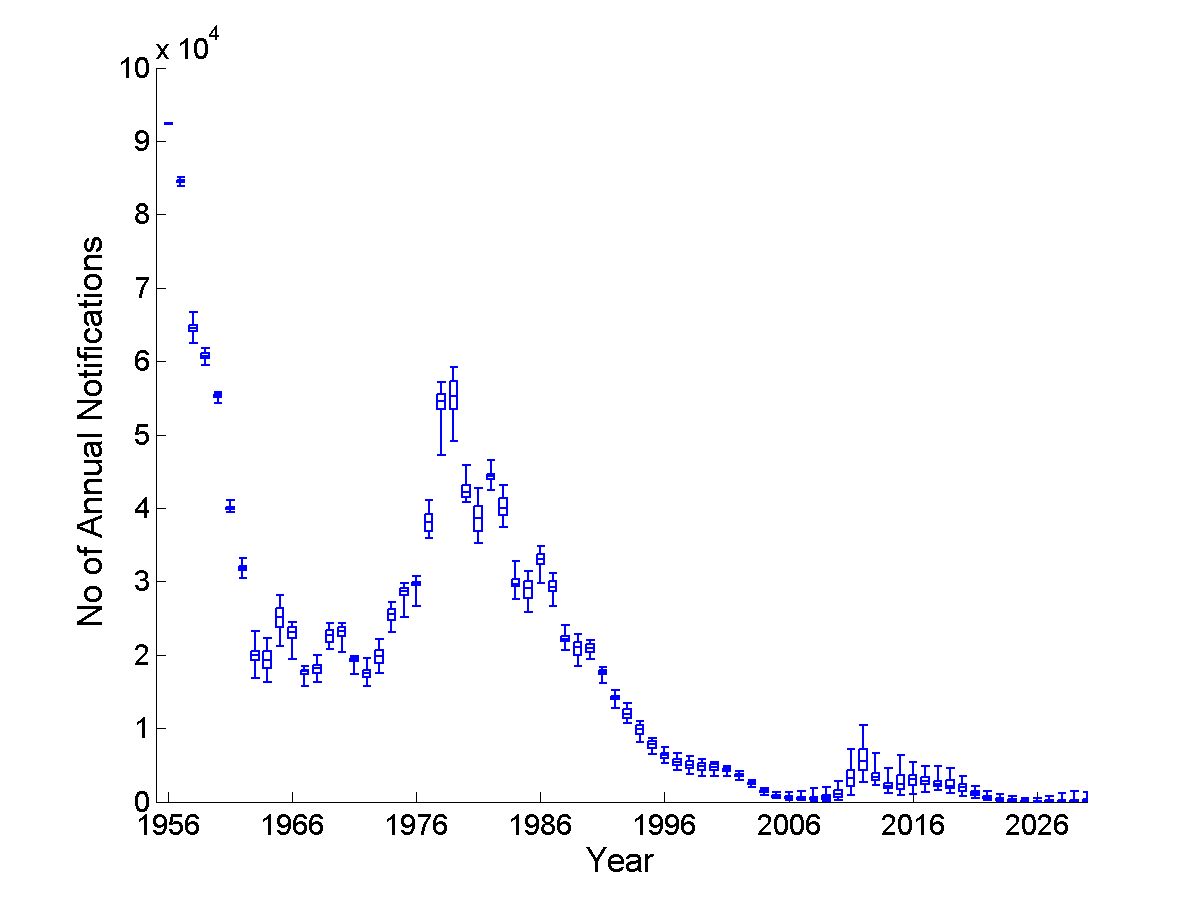

Supplement: Additional file 3: — Graphical User Interface porgramme to present pertussis simulation model results. (ZIP 8235 kb) [file 12916_2016_665_MOESM3_ESM.zip › POLYMOD_GUI_8_8.tif]

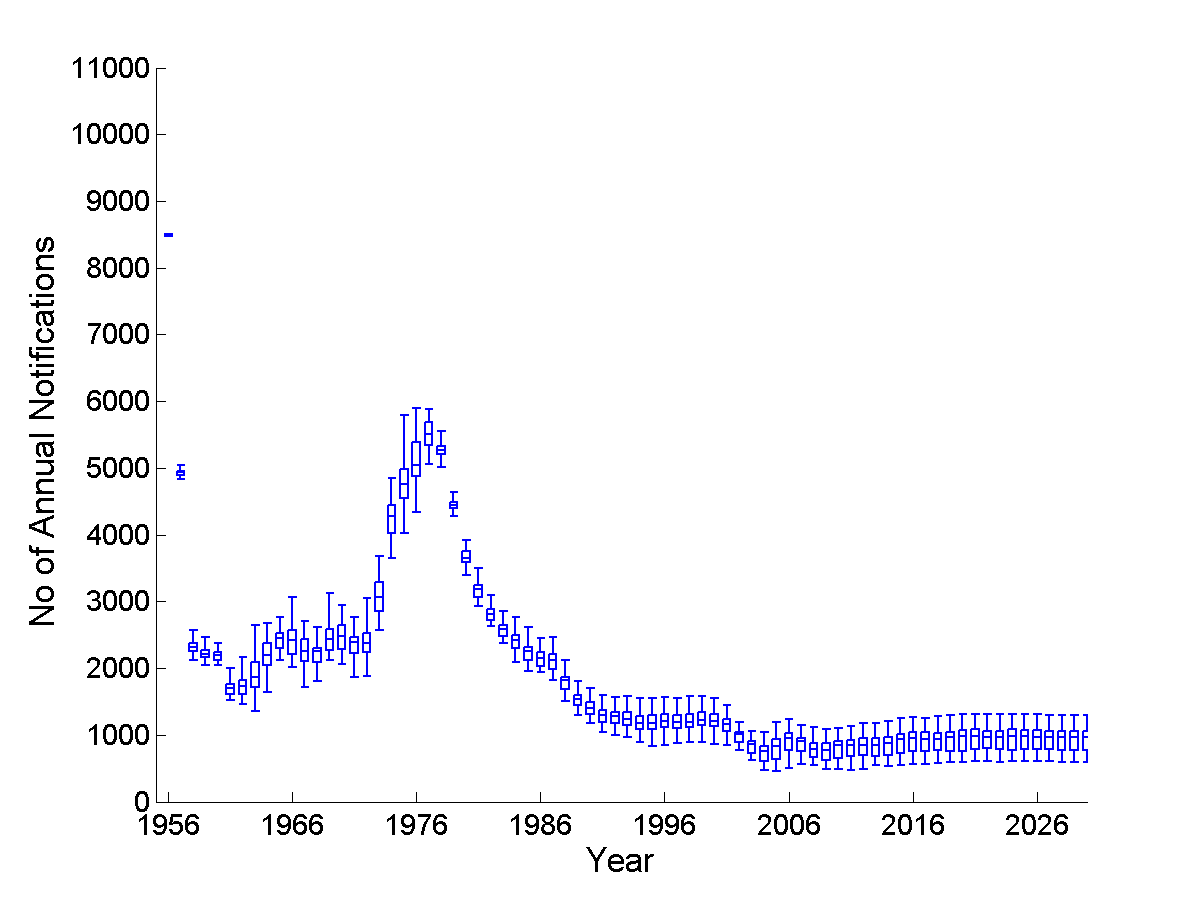

Supplement: Additional file 3: — Graphical User Interface porgramme to present pertussis simulation model results. (ZIP 8235 kb) [file 12916_2016_665_MOESM3_ESM.zip › POLYMOD_GUI_9_1.tif]

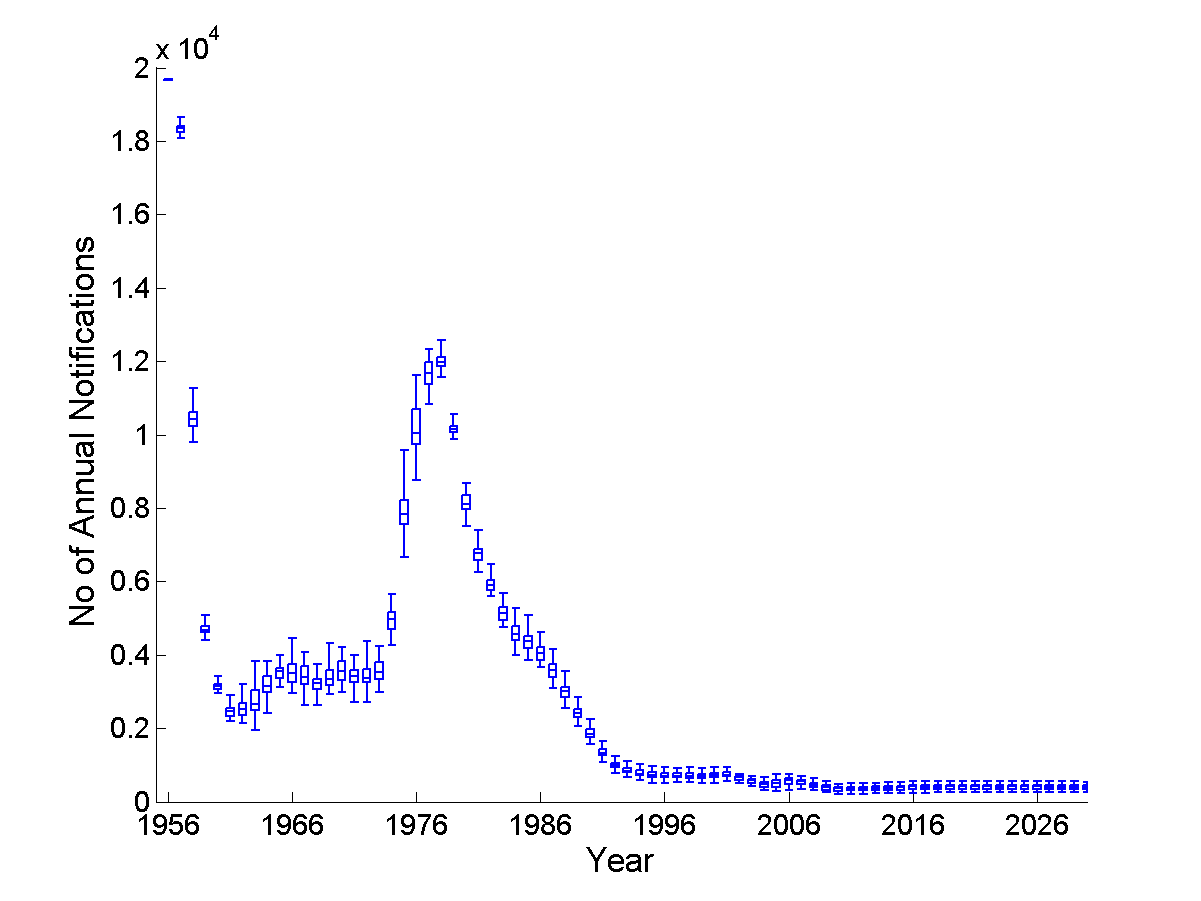

Supplement: Additional file 3: — Graphical User Interface porgramme to present pertussis simulation model results. (ZIP 8235 kb) [file 12916_2016_665_MOESM3_ESM.zip › POLYMOD_GUI_9_2.tif]

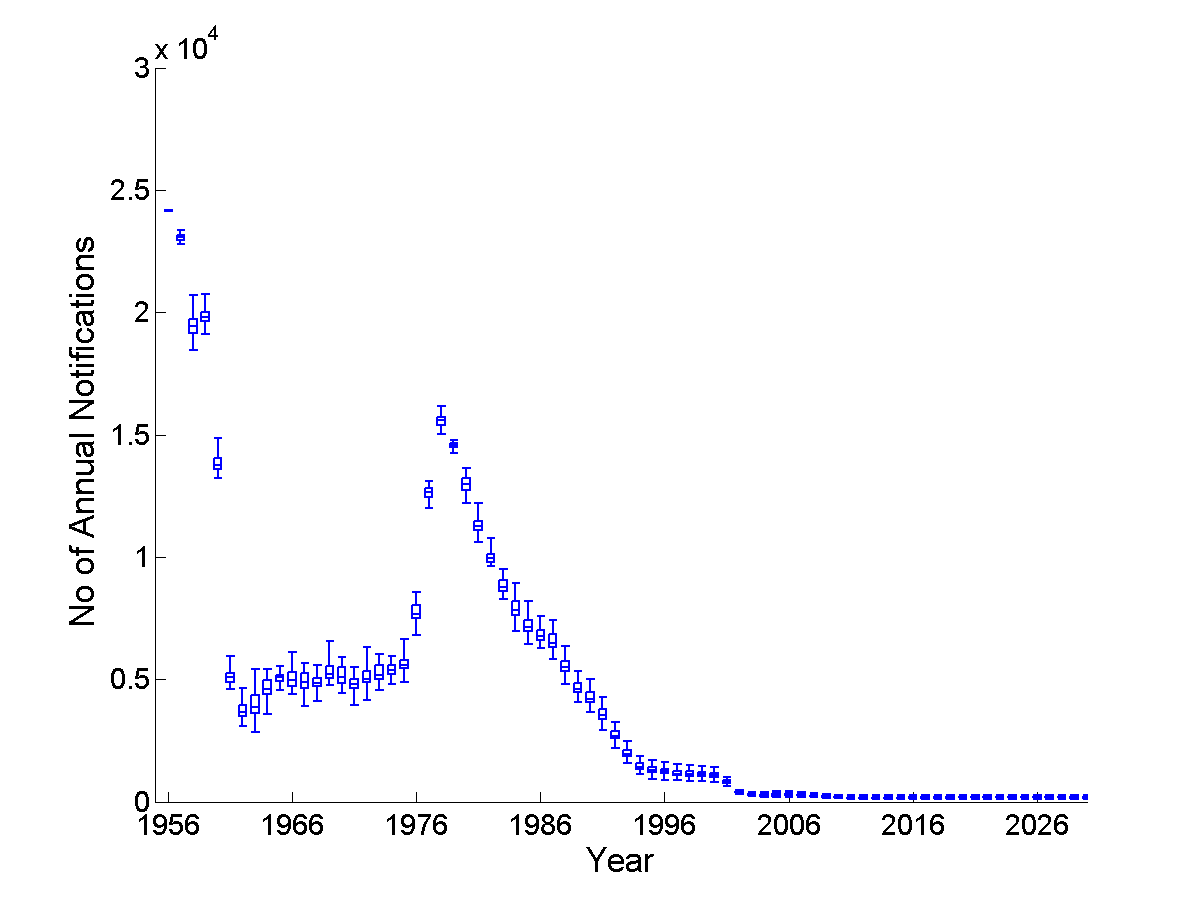

Supplement: Additional file 3: — Graphical User Interface porgramme to present pertussis simulation model results. (ZIP 8235 kb) [file 12916_2016_665_MOESM3_ESM.zip › POLYMOD_GUI_9_3.tif]

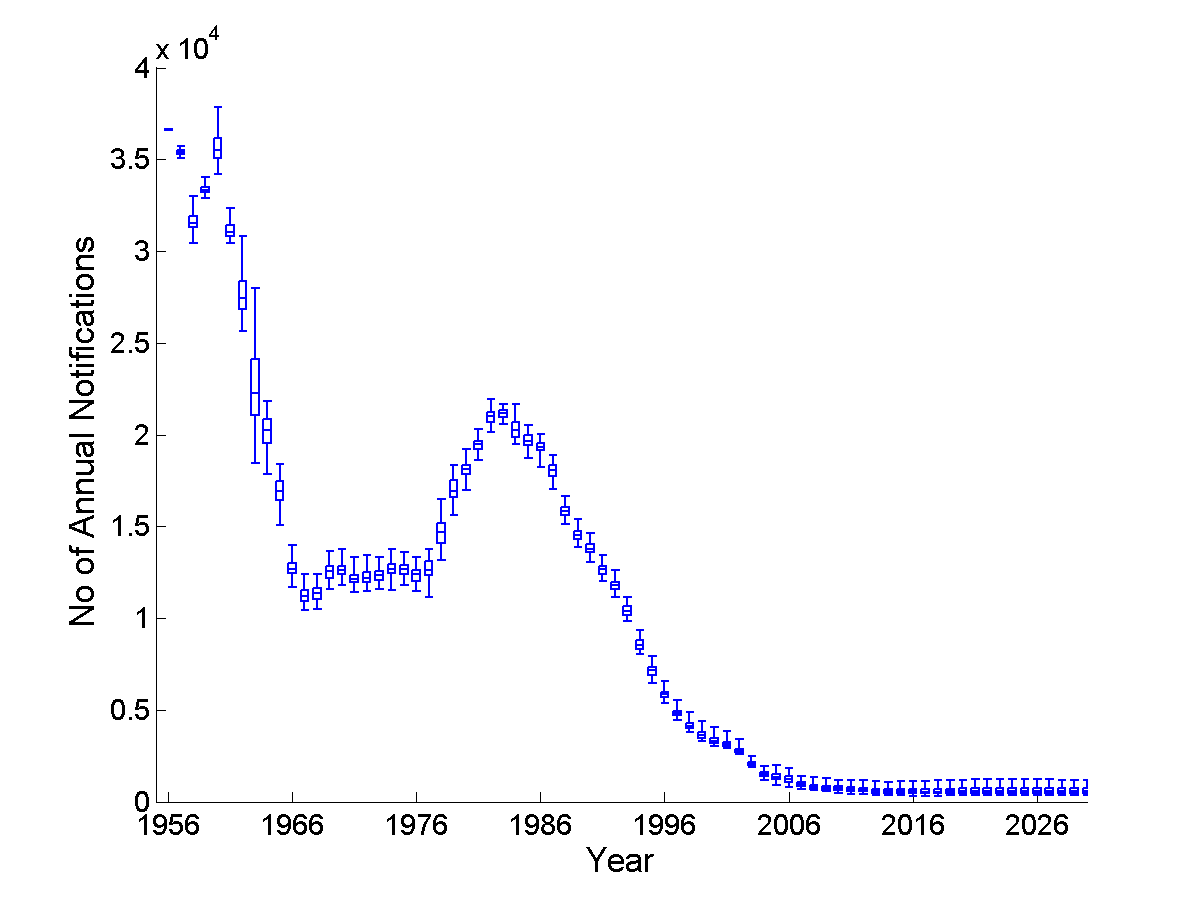

Supplement: Additional file 3: — Graphical User Interface porgramme to present pertussis simulation model results. (ZIP 8235 kb) [file 12916_2016_665_MOESM3_ESM.zip › POLYMOD_GUI_9_4.tif]

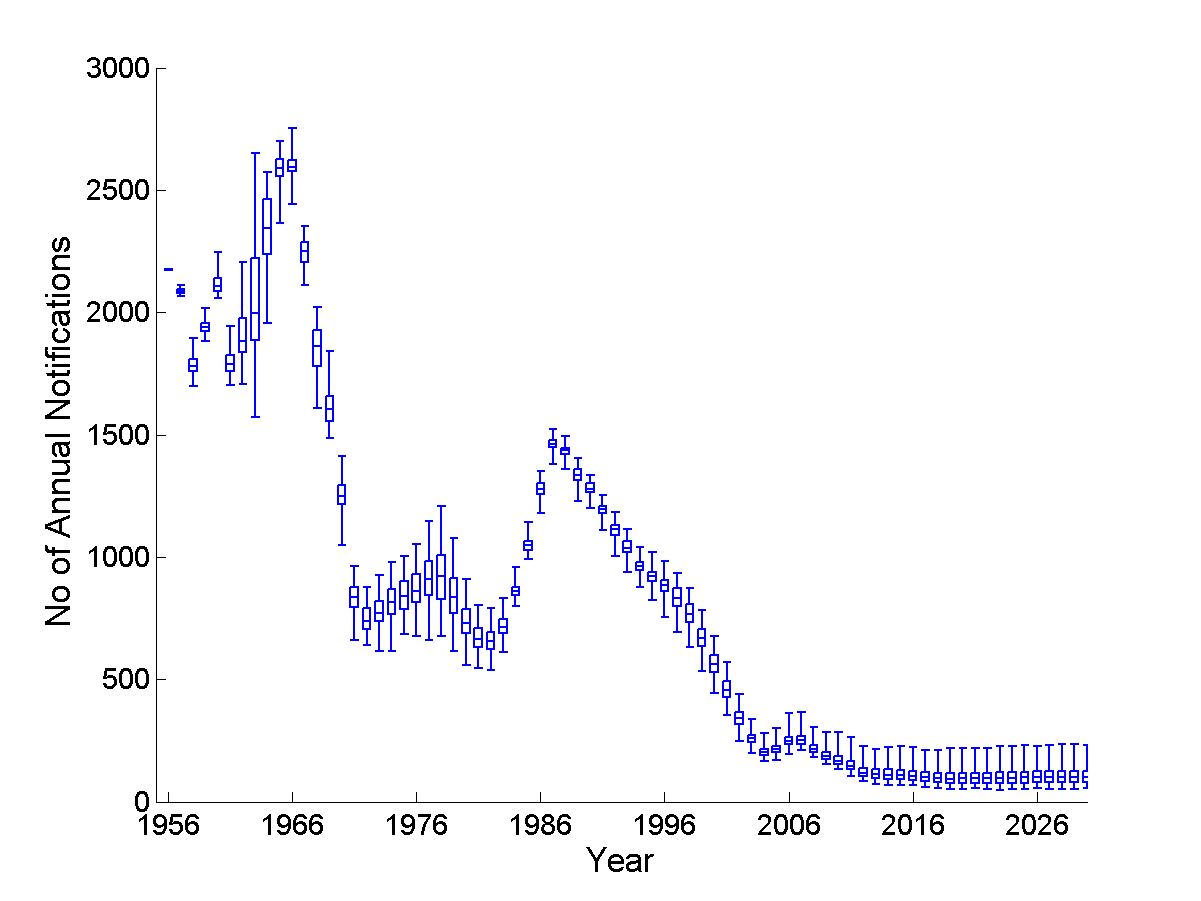

Supplement: Additional file 3: — Graphical User Interface porgramme to present pertussis simulation model results. (ZIP 8235 kb) [file 12916_2016_665_MOESM3_ESM.zip › POLYMOD_GUI_9_5.tif]

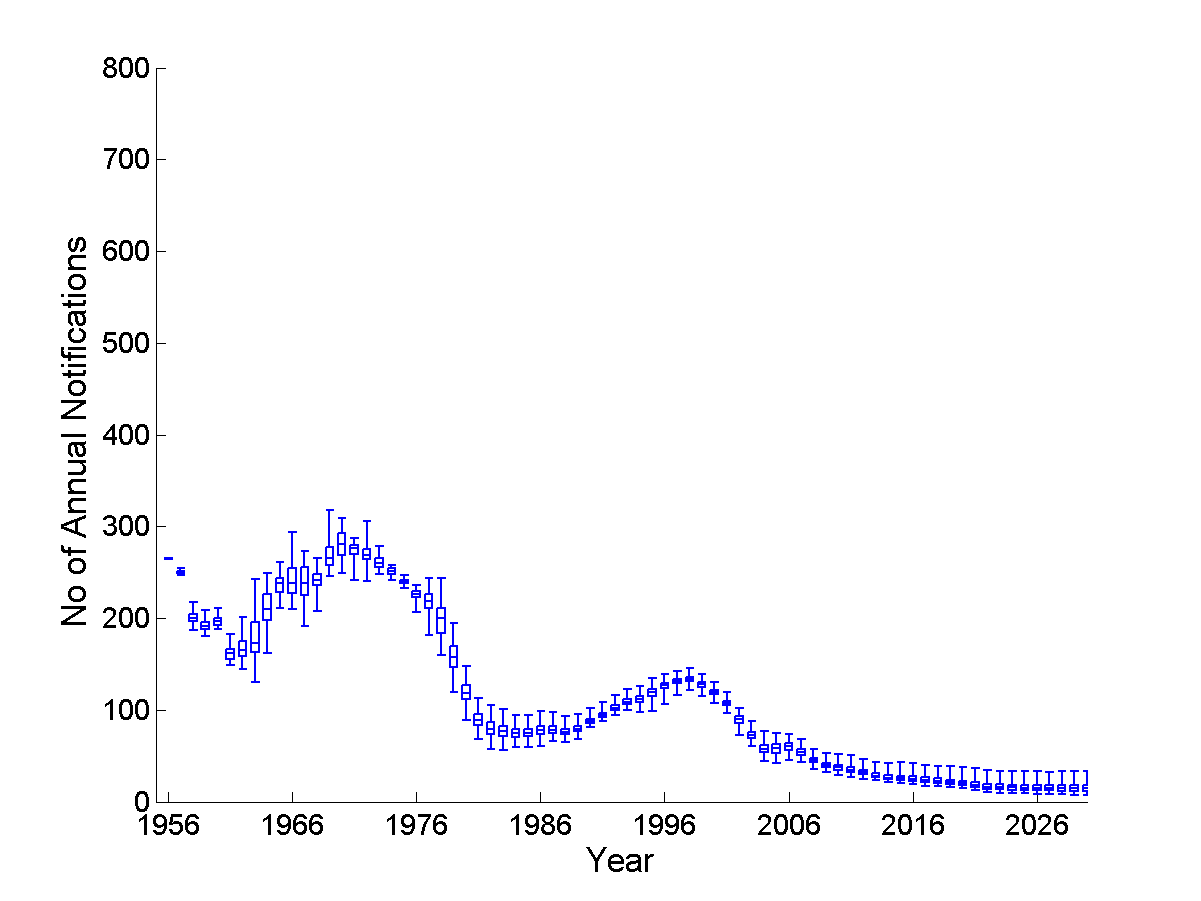

Supplement: Additional file 3: — Graphical User Interface porgramme to present pertussis simulation model results. (ZIP 8235 kb) [file 12916_2016_665_MOESM3_ESM.zip › POLYMOD_GUI_9_6.tif]

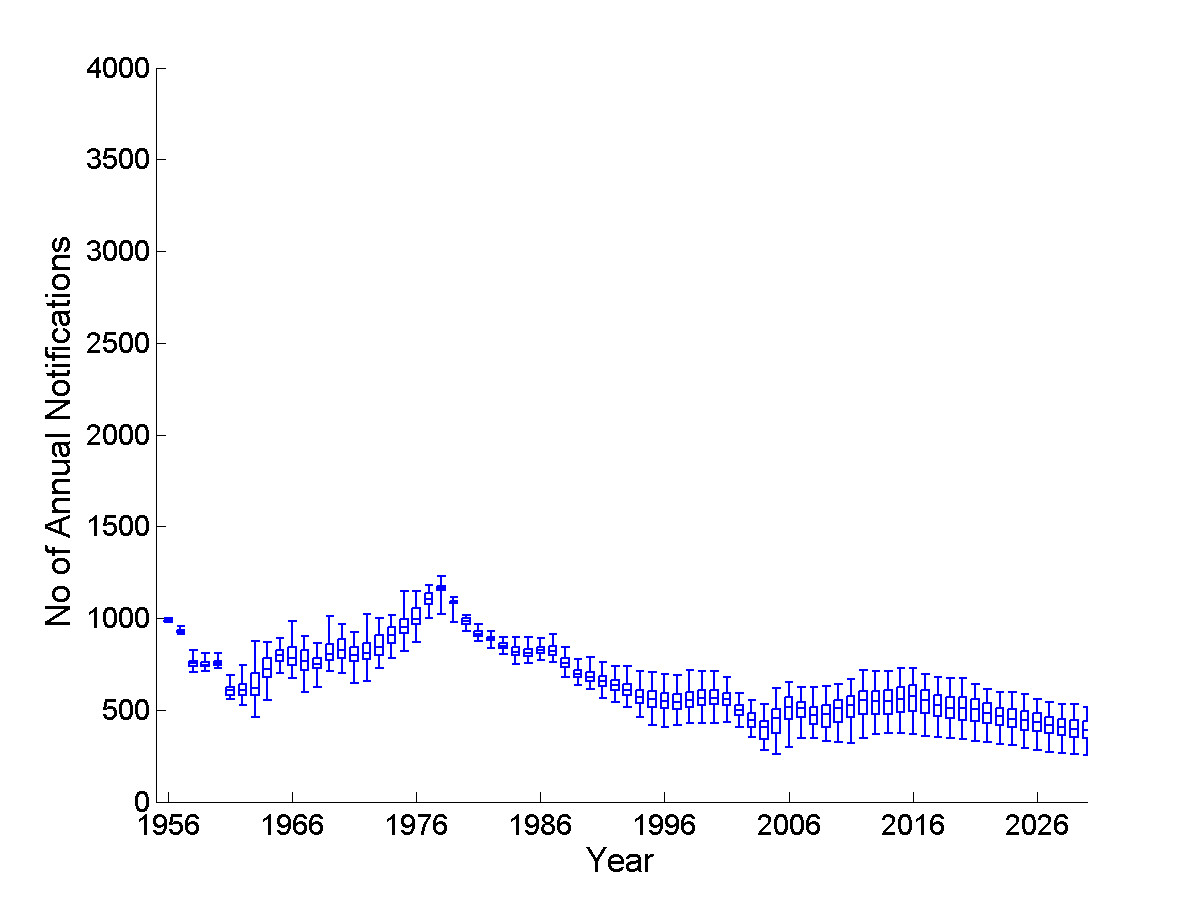

Supplement: Additional file 3: — Graphical User Interface porgramme to present pertussis simulation model results. (ZIP 8235 kb) [file 12916_2016_665_MOESM3_ESM.zip › POLYMOD_GUI_9_7.tif]

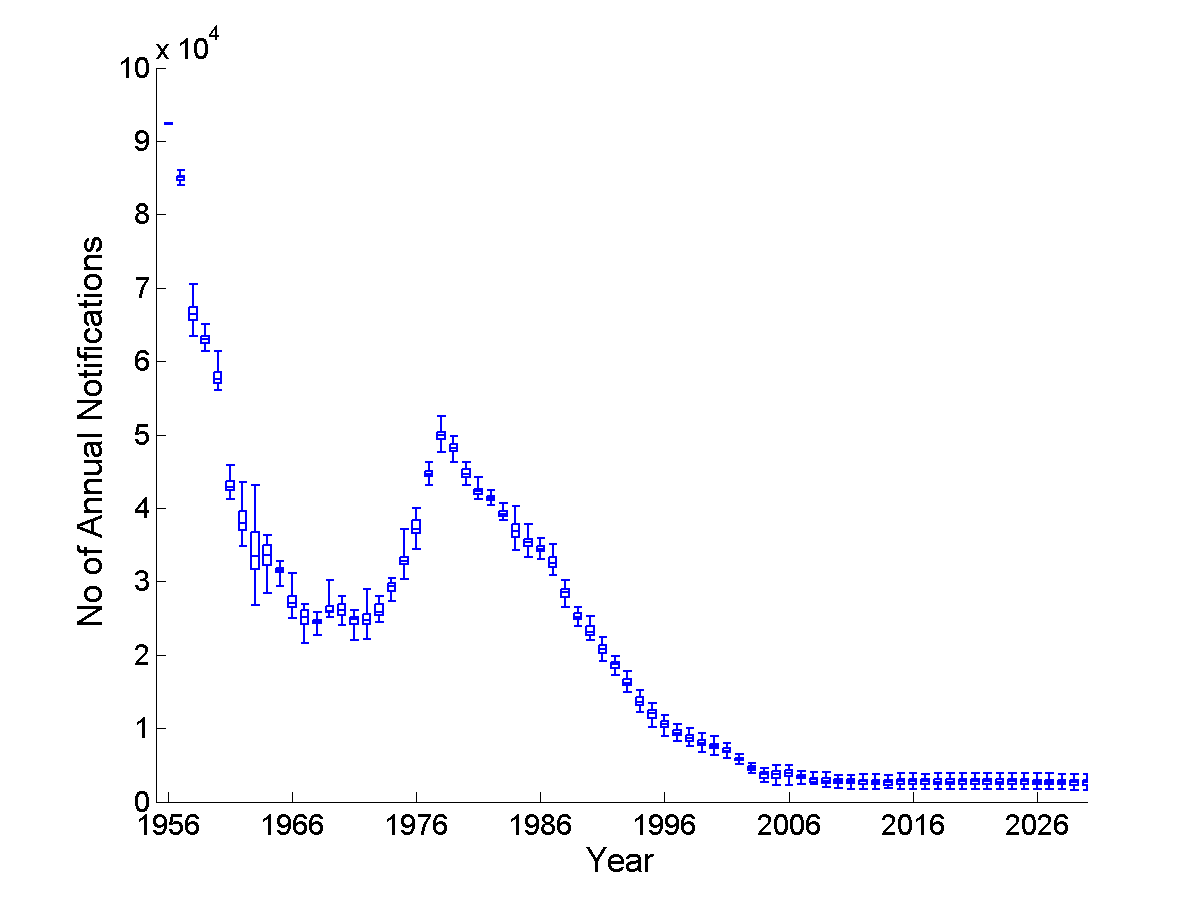

Supplement: Additional file 3: — Graphical User Interface porgramme to present pertussis simulation model results. (ZIP 8235 kb) [file 12916_2016_665_MOESM3_ESM.zip › POLYMOD_GUI_9_8.tif]

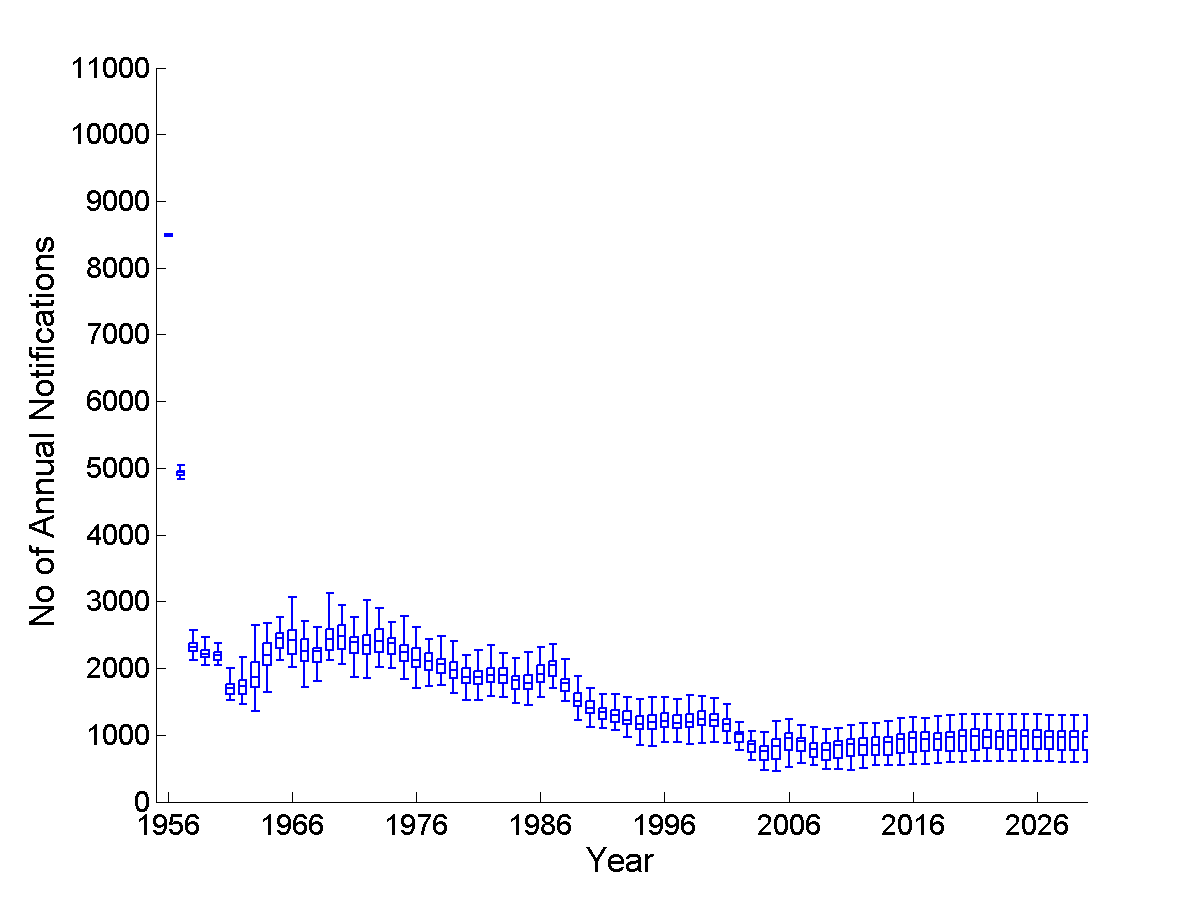

Supplement: Additional file 3: — Graphical User Interface porgramme to present pertussis simulation model results. (ZIP 8235 kb) [file 12916_2016_665_MOESM3_ESM.zip › POLYMOD_GUI_10_1.tif]

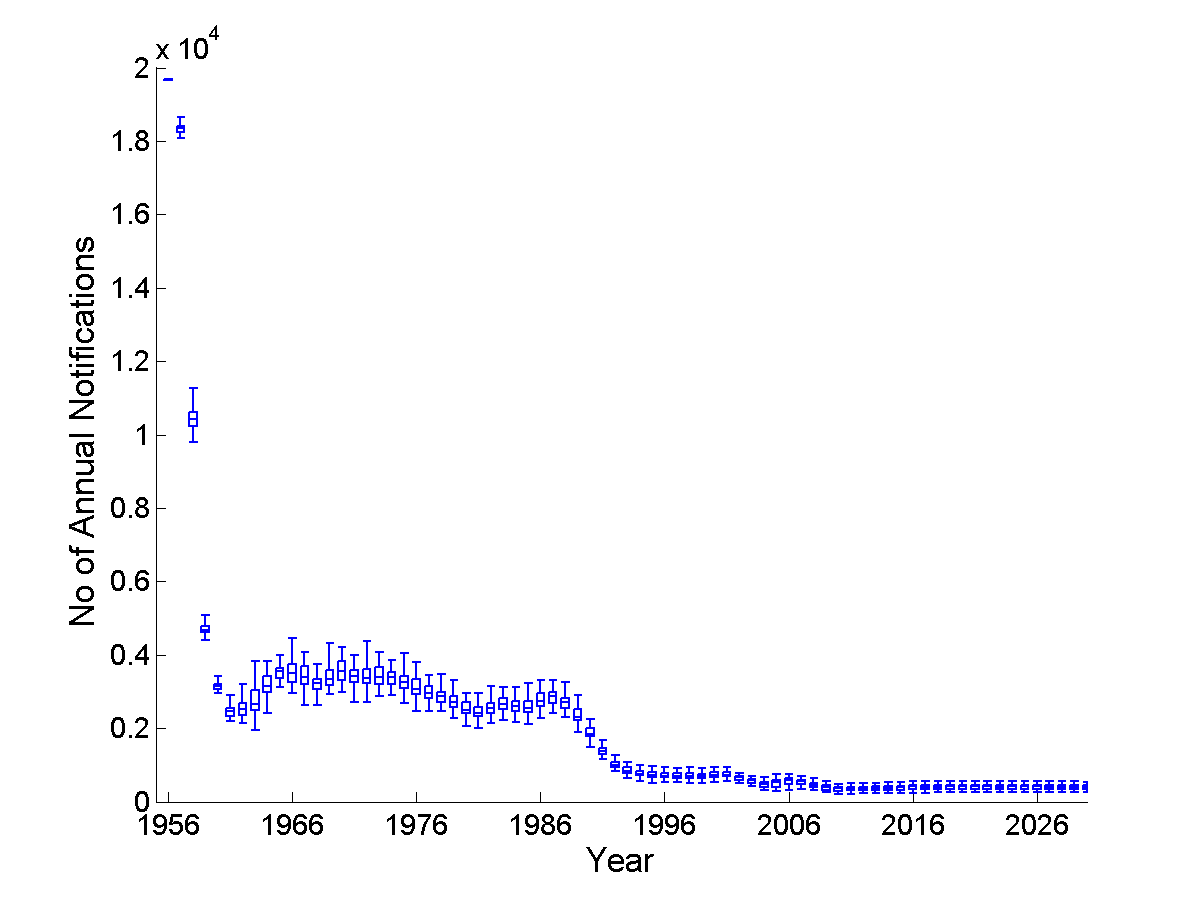

Supplement: Additional file 3: — Graphical User Interface porgramme to present pertussis simulation model results. (ZIP 8235 kb) [file 12916_2016_665_MOESM3_ESM.zip › POLYMOD_GUI_10_2.tif]

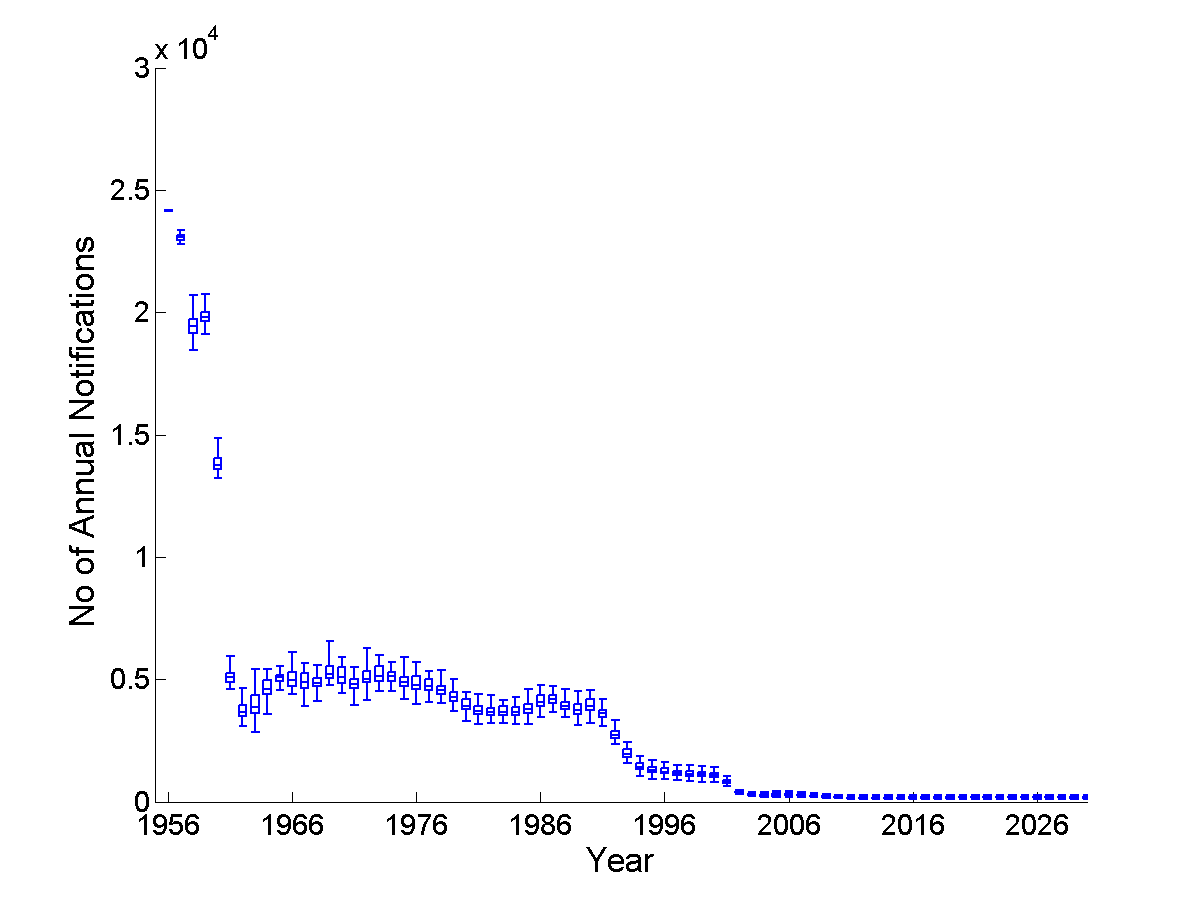

Supplement: Additional file 3: — Graphical User Interface porgramme to present pertussis simulation model results. (ZIP 8235 kb) [file 12916_2016_665_MOESM3_ESM.zip › POLYMOD_GUI_10_3.tif]

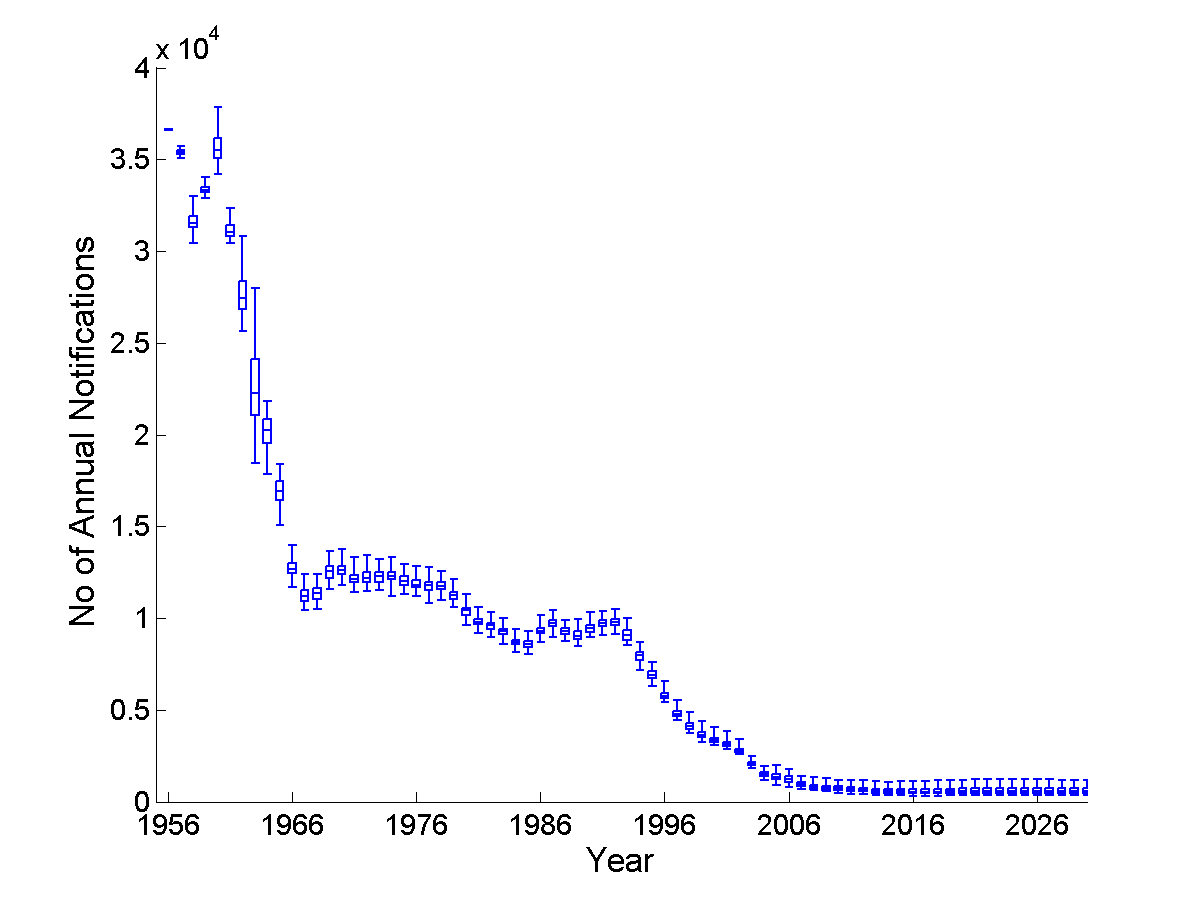

Supplement: Additional file 3: — Graphical User Interface porgramme to present pertussis simulation model results. (ZIP 8235 kb) [file 12916_2016_665_MOESM3_ESM.zip › POLYMOD_GUI_10_4.tif]

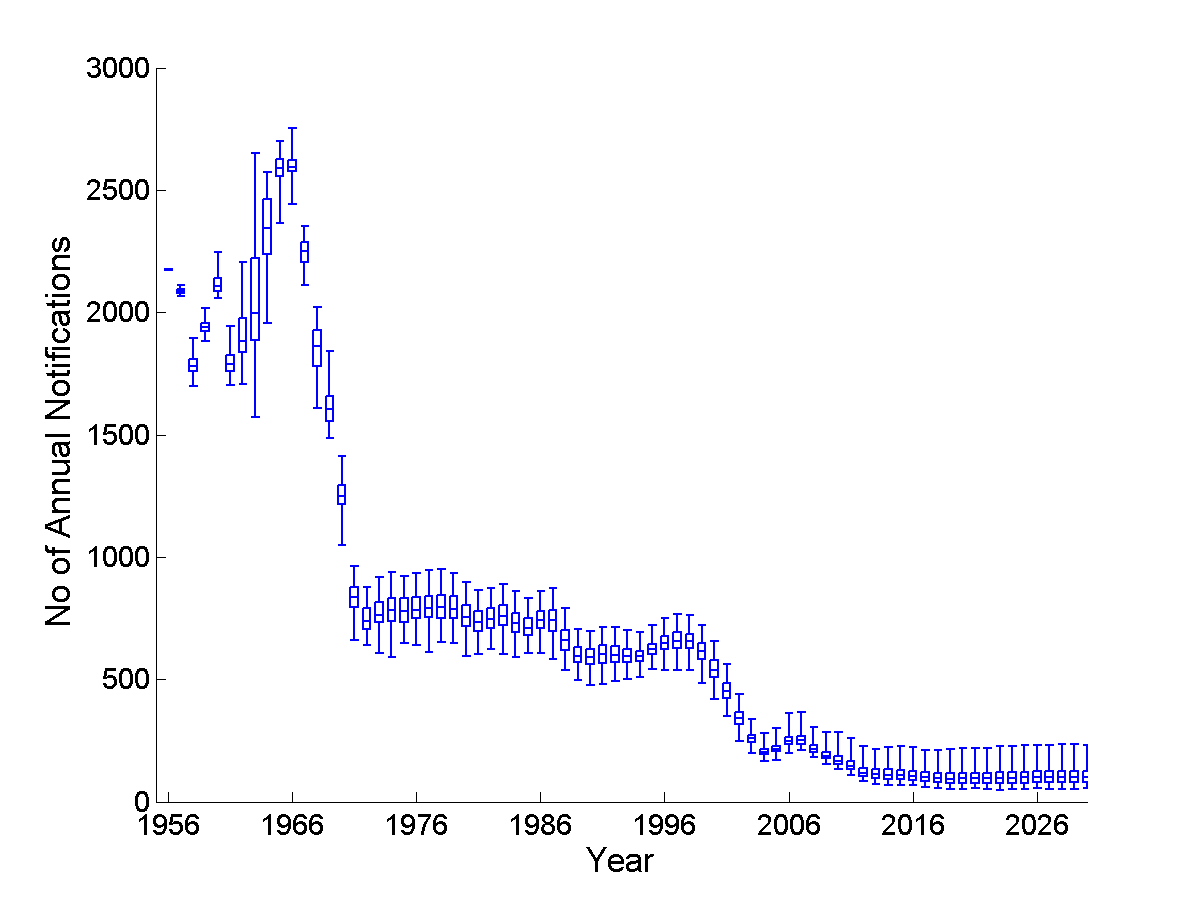

Supplement: Additional file 3: — Graphical User Interface porgramme to present pertussis simulation model results. (ZIP 8235 kb) [file 12916_2016_665_MOESM3_ESM.zip › POLYMOD_GUI_10_5.tif]

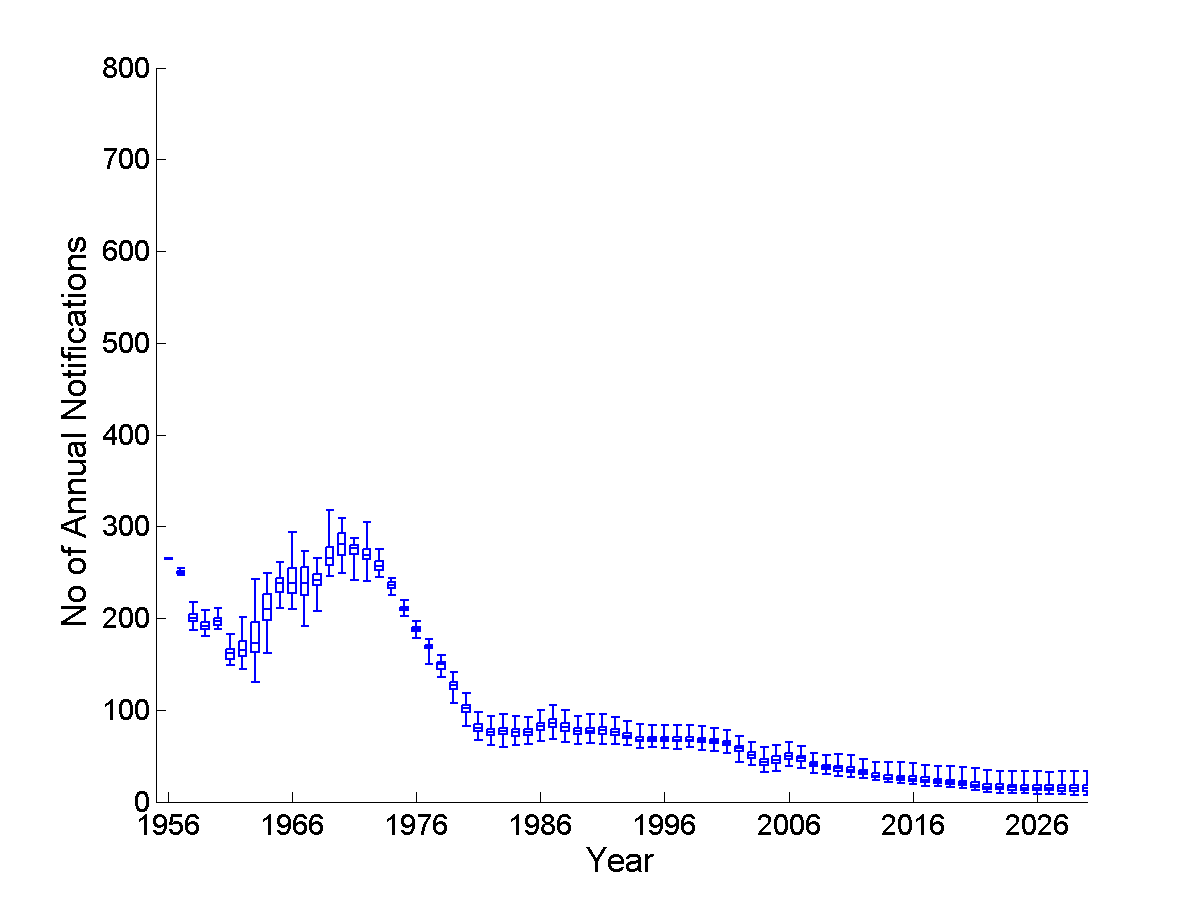

Supplement: Additional file 3: — Graphical User Interface porgramme to present pertussis simulation model results. (ZIP 8235 kb) [file 12916_2016_665_MOESM3_ESM.zip › POLYMOD_GUI_10_6.tif]

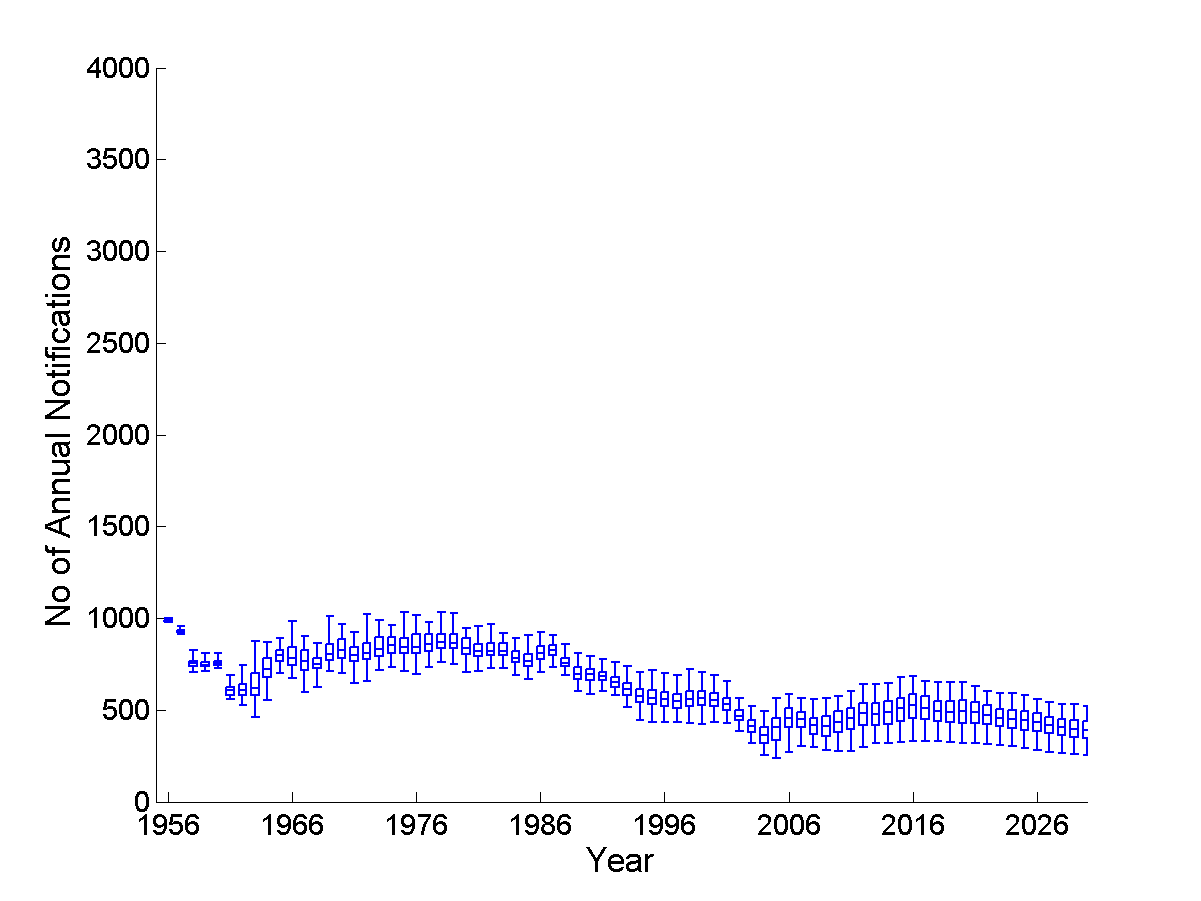

Supplement: Additional file 3: — Graphical User Interface porgramme to present pertussis simulation model results. (ZIP 8235 kb) [file 12916_2016_665_MOESM3_ESM.zip › POLYMOD_GUI_10_7.tif]

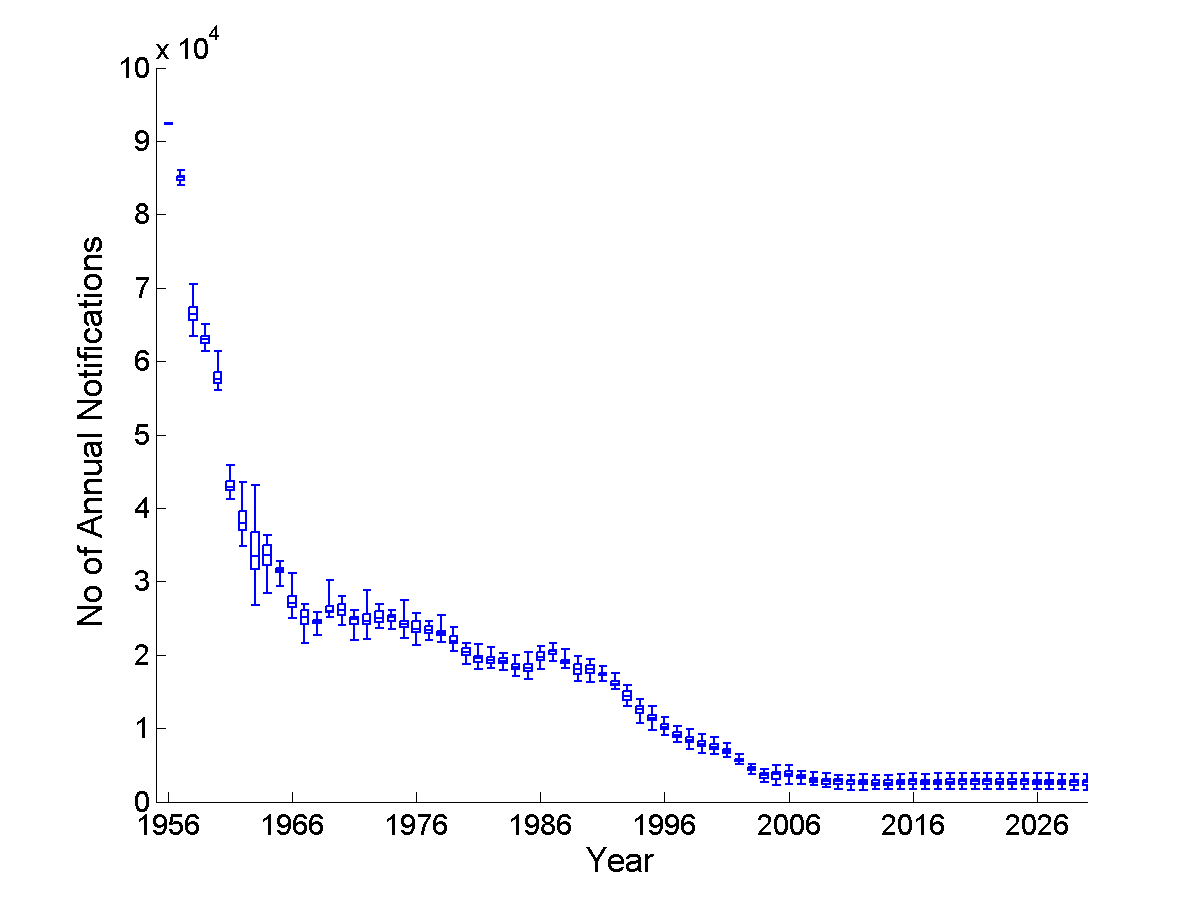

Supplement: Additional file 3: — Graphical User Interface porgramme to present pertussis simulation model results. (ZIP 8235 kb) [file 12916_2016_665_MOESM3_ESM.zip › POLYMOD_GUI_10_8.tif]

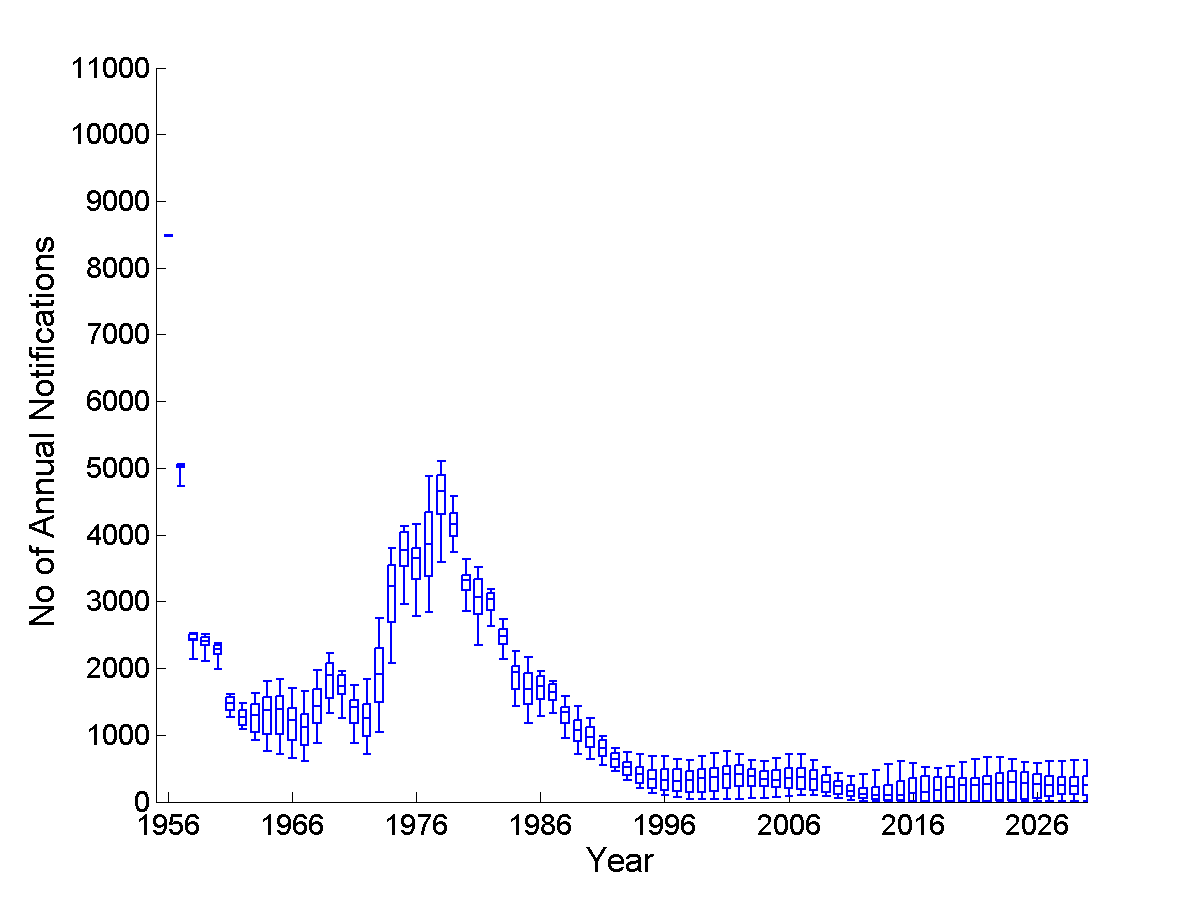

Supplement: Additional file 3: — Graphical User Interface porgramme to present pertussis simulation model results. (ZIP 8235 kb) [file 12916_2016_665_MOESM3_ESM.zip › WAIFW_GUI_0_1.tif]

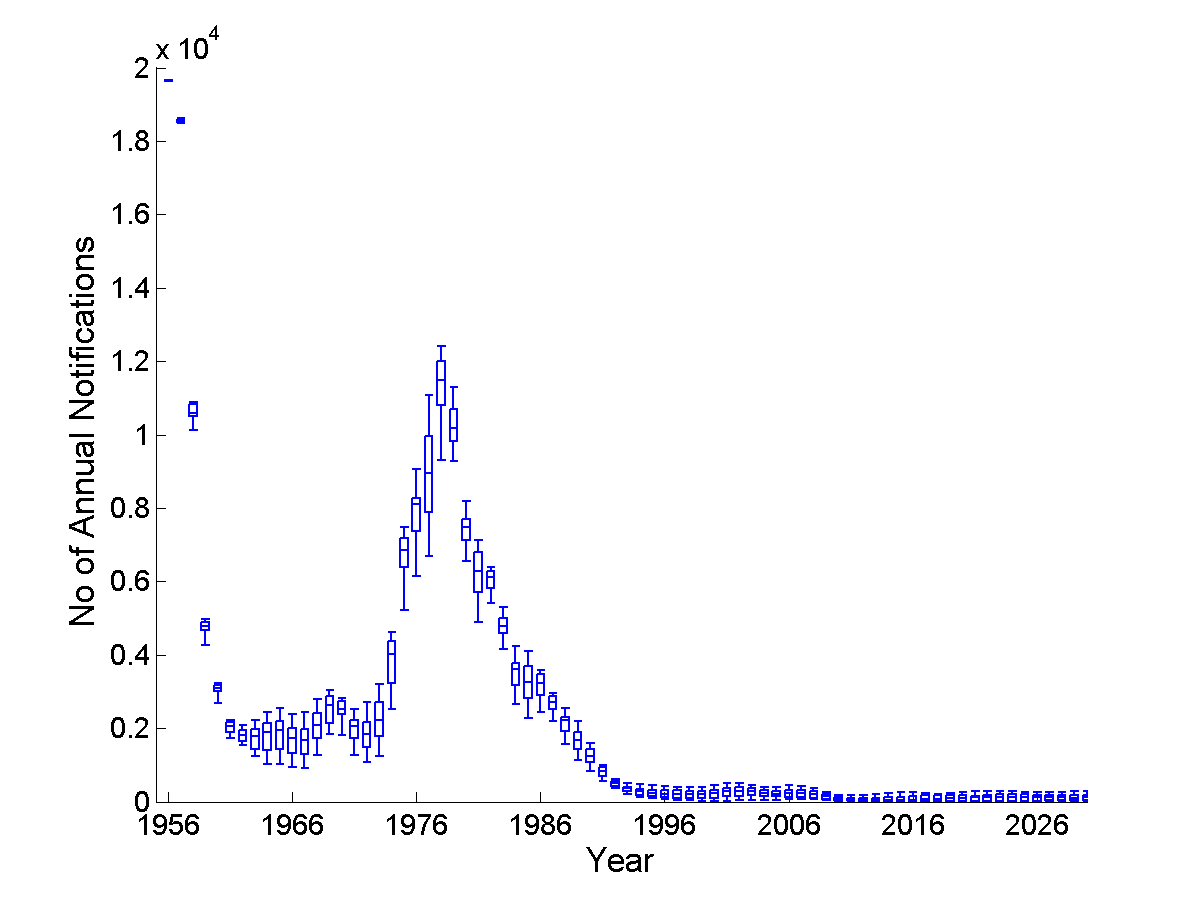

Supplement: Additional file 3: — Graphical User Interface porgramme to present pertussis simulation model results. (ZIP 8235 kb) [file 12916_2016_665_MOESM3_ESM.zip › WAIFW_GUI_0_2.tif]

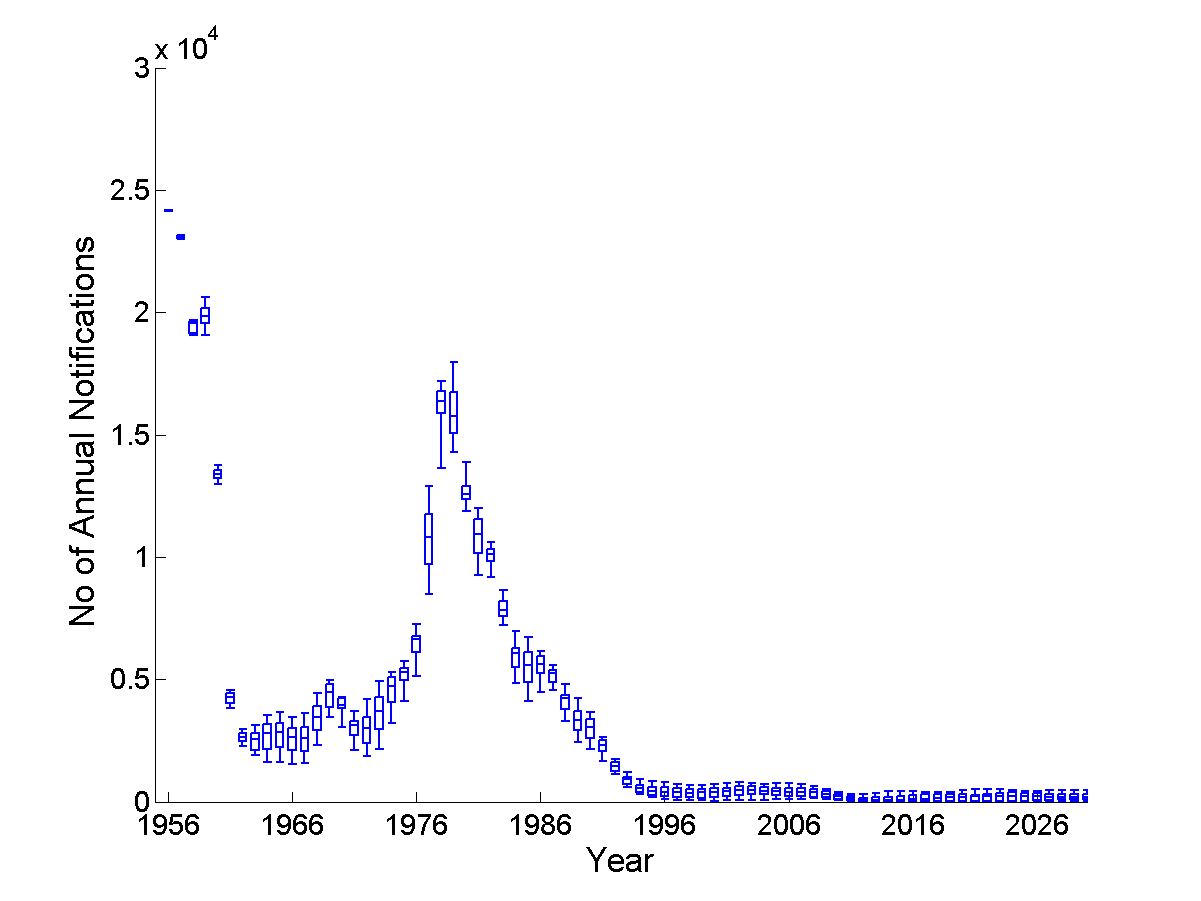

Supplement: Additional file 3: — Graphical User Interface porgramme to present pertussis simulation model results. (ZIP 8235 kb) [file 12916_2016_665_MOESM3_ESM.zip › WAIFW_GUI_0_3.tif]
